# Author Correction: Extant and extinct bilby genomes combined with Indigenous knowledge improve conservation of a unique Australian marsupial

**DOI:** 10.1038/s41559-024-02519-0

**Published:** 2024-07-29

**Authors:** Carolyn J. Hogg, Richard J. Edwards, Katherine A. Farquharson, Luke W. Silver, Parice Brandies, Emma Peel, Merly Escalona, Frederick R. Jaya, Rujiporn Thavornkanlapachai, Kimberley Batley, Tessa M. Bradford, J. King Chang, Zhiliang Chen, Nandan Deshpande, Martin Dziminski, Kyle M. Ewart, Oliver W. Griffith, Laia Marin Gual, Katherine L. Moon, Kenny J. Travouillon, Paul Waters, Camilla M. Whittington, Marc R. Wilkins, Kristofer M. Helgen, Nathan Lo, Simon Y. W. Ho, Aurora Ruiz Herrera, Rachel Paltridge, Jennifer A. Marshall Graves, Marilyn Renfree, Beth Shapiro, Kym Ottewell, Kiwirrkurra Rangers, Conway Gibson, Raymond Maxwell, Zecharia Spencer, Yalti Napangati, Mary Butler, Janine West, John West, Mantua James, Nolia Napangati, Loretta Gibson, Payu West, Angus Gibson, Scott West, Kim West, Walimpirri Japaltjari, Ed Blackwood, Rachel Paltridge, Katherine Belov

**Affiliations:** 1 School of Life and Environmental Sciences, The University of Sydney, Sydney, New South Wales Australia; 2 ARC Centre of Excellence for Innovations in Peptide and Protein Science, The University of Sydney, Sydney, New South Wales Australia; 3 Minderoo OceanOmics Centre at UWA, Oceans Institute, The University of Western Australia, Perth, Western Australia Australia; 4 School of Biotechnology and Biomolecular Sciences, UNSW Sydney, Sydney, New South Wales Australia; 5 Department of Ecology and Evolutionary Biology, University of California Santa Cruz, Santa Cruz, CA USA; 6 Biodiversity and Conservation Science, Department of Biodiversity, Conservation and Attractions, Kensington, Western Australia Australia; 7 Evolutionary Biology Unit, South Australian Museum, Adelaide, South Australia Australia; 8 School of Biological Sciences, The University of Adelaide, Adelaide, South Australia Australia; 9 Illumina, Melbourne, Victoria Australia; 10 Ramaciotti Centre for Genomics and School of Biotechnology and Biomolecular Science, UNSW, Sydney, New South Wales Australia; 11 School of Natural Sciences, Macquarie University, Sydney, New South Wales Australia; 12 Departament de Biologia Cel·lular, Fisiologia i Immunologia, Universitat Autònoma de Barcelona, Cerdanyola del Vallès, Spain; 13 Genome Integrity and Instability Group, Institut de Biotecnologia i Biomedicina, Universitat Autònoma de Barcelona, Cerdanyola del Vallès, Spain; 14 Howard Hughes Medical Institute, University of California Santa Cruz, Santa Cruz, CA USA; 15 Collections and Research, Western Australian Museum, Welshpool, Western Australia Australia; 16 Australian Museum Research Institute, Australian Museum, Sydney, New South Wales Australia; 17 Indigenous Desert Alliance, Alice Springs, Northern Territory Australia; 18 Department of Environment and Genetics, La Trobe University, Melbourne, Victoria Australia; 19 School of BioSciences, University of Melbourne, Melbourne, Victoria Australia; 20 Kiwirrkura Community, Gibson Desert, Western Australia Australia

**Keywords:** Conservation genomics, Population dynamics, Evolutionary genetics

Correction to: *Nature Ecology & Evolution* 10.1038/s41559-024-02436-2, published online 1 July 2024.

In the version of the article initially published, in the “Bilby chromosomes” section, the sentence “The Ninu genome provides insights into chromosome evolution showing the Ninu XY_1_Y_2_ system was generated by fusion of the X with a telocentric autosome” has now been corrected to “…fusion of the X with the long arm of an autosome” in the HTML and PDF versions of the article.

