# Supplementary material for: Extant and extinct bilby genomes combined with Indigenous knowledge improve conservation of a unique Australian marsupial
Source: Nat Ecol Evol. 2024 Jul 1;8(7):1311–26. doi: 10.1038/s41559-024-02436-2 (PMC11239497; doi:10.1038/s41559-024-02436-2)
Supplement: Supplementary file 1 — Supplementary Figs. 1–12, Tables 1–19, Notes and References [file 41559_2024_2436_MOESM1_ESM.pdf]

nature ecology & evolution

Supplementary information

https://doi.org/10.1038/s41559-024-02436-2

Extant and extinct bilby genomes combined with Indigenous knowledge improve conservation of a unique Australian marsupial

In the format provided by the authors and unedited

Extant and extinct bilby genomes combined with Indigenous knowledge improve conservation of a unique Australian marsupial

Carolyn J. Hogg[1,2*#], Richard J. Edwards[3,4*], Katherine A. Farquharson[1,2*], Luke W. Silver[1], Parice Brandies[1], Emma Peel[1,2], Merly Escalona[5], Frederick R. Jaya[1], Rujiporn Thavornkanlapachai[6], Kimberley Batley[1], Tessa M. Bradford[7,8], J King Chang[4], Zhiliang Chen[9], Nandan Deshpande[4,10], Martin Dziminski[6], Kyle M. Ewart[1], Oliver W. Griffith[11], Laia Marin-Gual[12,13] Katherine L. Moon[5,14], Kenny J. Travouillon[15], Paul Waters[4], Camilla M. Whittington[1], Marc R. Wilkins[4,10], Kristofer M. Helgen[16], Nathan Lo[1], Simon Y. W. Ho[1], Aurora Ruiz Herrera[12,13], Rachel Paltridge[17], Jennifer A. Marshall Graves[18], Marilyn Renfree[19], Beth Shapiro[5,14], Kym Ottewell[6], Kiwirrkurra Rangers[20], Katherine Belov[1,2]

[1] School of Life and Environmental Sciences, The University of Sydney, NSW, Australia

[2] ARC Centre of Excellence for Innovations in Peptide and Protein Science, The University of Sydney, NSW, Australia

[3] Minderoo OceanOmics Centre at UWA Oceans Institute, The University of Western Australia, Perth, WA, Australia

[4] School of Biotechnology and Biomolecular Sciences, UNSW Sydney, Sydney, NSW, Australia

[5] Department of Ecology and Evolutionary Biology, University of California Santa Cruz, Santa Cruz, CA, USA

[6] Biodiversity and Conservation Science, Department of Biodiversity, Conservation and Attractions, Kensington, WA 6151, Australia

[7] Evolutionary Biology Unit, South Australian Museum, Adelaide, SA, Australia

[8] School of Biological Sciences, The University of Adelaide, Adelaide, SA, Australia

[9] Illumina, Melbourne, VIC, Australia

[10] Ramaciotti Centre for Genomics and School of Biotechnology and Biomolecular Science, UNSW Sydney

[11] School of Natural Sciences, Macquarie University, Sydney, NSW, Australia

[12] Departament de Biologia Cel·lular, Fisiologia i Immunologia, Universitat Autònoma de Barcelona, Cerdanyola del Vallès, Spain.

[13] Genome Integrity and Instability Group, Institut de Biotecnologia i Biomedicina, Universitat Autònoma de Barcelona, Cerdanyola del Vallès, Spain.

[14] Howard Hughes Medical Institute, University of California Santa Cruz, Santa Cruz, CA, USA

[15] Collections and Research, Western Australian Museum, Welshpool, WA, Australia

[16] Australian Museum Research Institute, Australian Museum, Sydney, NSW, Australia

[17] Indigenous Desert Alliance, Alice Springs, NT, Australia

[18] Department of Environment and Genetics, La Trobe University, Melbourne, Victoria, Australia

[19] School of BioSciences, University of Melbourne, Melbourne, Victoria, Australia
[20] Kiwirrkurra Community, Gibson Desert, WA, Australia

* contributed equally; # corresponding author
Corresponding author: Carolyn Hogg (carolyn.hogg@sydney.edu.au)

Table of Contents

Supplementary Tables 4
Supplementary Figures 24
Supplementary Notes 28
1 Ninu Genome Sequencing, Assembly and Analysis 28
1.1 Sample Collection and DNA extraction 28
1.2 Genome library preparation and sequencing 28
1.3 Genome assembly 28
1.4 Mitochondrial genome assembly 30
1.5 Manual curation of nuclear genome assembly 30
1.6 Assembly quality assessment 30
1.7 Resequenced genomes 31
1.8 Genome-wide association study 34
2 Bilby population genomics 35
2.1 Historical population size 35
2.2 Runs of Homozygosity (ROH) analysis 37
2.3 Managed Metapopulation History and Study Sites 40
2.4 Metapopulation Sample Collection and Analyses 42
2.5 MassARRAY SNP Panel Design 46
2.6 Management Recommendations 52
3 Ninu Genome Annotation and Gene Family Investigation 53
3.1 RNA extractions and transcriptome sequencing 53
3.2 Transcriptome assembly 53
3.3 Genome annotation 54
3.4 Chromosome synteny plots 56
3.5 X-chromosome read depth and meiotic immunofluorescence 56
3.6 Gene family investigation 56
3.7 Olfactory receptor genes 59
3.8 Characterisation of Ninu immune genes 60
4 Supplementary References 62

Supplementary Tables

Table S1: Individuals used for the Ninu and Yallara resequenced genomes including Sample ID, sex, collection location and date, sample type, tissue preservation and subspecies. Coverage in brackets is the coverage per sample when combined with the Yallara data for the Yallara phylogenetic analyses.

Species	Location sampled	Sample ID	Sex	Tissue Type	Collection Year	Coverage	No. Variants
Macrotis lagotis	Temperate	956000004913012	F	ear biopsy	1/10/2019	23.7	16313234
Macrotis lagotis	Temperate	0007C7B153	F	ear biopsy	1/10/2019	21.4	15751276
Macrotis lagotis	Temperate	0007C7B720	F	ear biopsy	1/10/2019	22.9	16648688
*Macrotis lagotis**	Temperate	0007C7C1A6	M	ear biopsy	30/09/2019	13.8 (10.39)	16156427
*Macrotis lagotis**	Temperate	0007C7AFE8	M	ear biopsy	1/10/2019	26.4 (20.99)	15989712
*Macrotis lagotis**	Temperate	7C7B6DB	M	ear biopsy	30/09/2019	28 (22.25)	16251843
Macrotis lagotis	Semi-arid	000786C0A7	F	ear biopsy	11/10/2019	22.6	17815109
Macrotis lagotis	Semi-arid	0007DE091B	F	ear biopsy	9/12/2019	22.8	17719028
Macrotis lagotis	Semi-arid	2530351	F	ear biopsy	15/01/2020	24	17449262
*Macrotis lagotis**	Semi-arid	10002530249	M	ear biopsy	15/01/2020	26.2 (19.88)	17554347
*Macrotis lagotis**	Semi-arid	0007B116F9	M	ear biopsy	10/10/2019	29.6 (23.20)	17525424
*Macrotis lagotis**	Semi-arid	0007B11508	M	ear biopsy	2019	25.4 (19.18)	17405821
*Macrotis leucura**	Unknown (British Museum of Natural History)	NHMU1883.10.19.17 (Sequence ID: A1)	M	skin	Unknown	2.2 (1st run) 9.28 (2nd run)	
*Macrotis leucura minor**,[#]	Northern Territory (Museums Victoria)	NMVC7087 (Sequence ID: A2)	M	skull scraping	1898	7.9 (1st run) 12.82 (2nd run)	
Macrotis leucura minor	Northern Territory (Museums Victoria)	NMVC7091 (Sequence ID: A3)	M	bone preserved in spirit	1895	Failed QC	

Species	Location sampled	Sample ID	Sex	Tissue Type	Collection Year	Coverage	No. Variants
Macrotis leucura	Northern Territory (Museums Victoria)	NMVC7295 (Sequence ID: A7)	F	liver sample in spirit	1895	0.73	
Macrotis leucura miselius	South Australia (South Australian Museum)	SAMAM3465 (Sequence ID: A9)	U	skin sample	1931	0.99	

* Ninu and Yallara used in Yallara phylogenetic analyses
[#] Individual used to generate the Yallara reference genome

Table S2: Runs of homozygosity (ROH) statistics for the 12 re-sequenced Ninu, based on 29,266,950 SNPs (all missing data were removed). F_{ROH} WGSwas based on the total ROH length divided by the total possible length of ROH (i.e. >98% of the genome length). Observed heterozygosity (H_O) and inbreeding coefficients were colour-coded from most diverse (blue) to least diverse (red).

Animal ID	Source pop	H_O	# of ROH	Total length of ROHs (kb)	Average ROH length (kb)	Length class (in Mb)					F_{ROH} for ROHs >100 kb	F_{ROH} for ROHs >500 kb	F_{ROH} for ROHs >1 Mb
						0-0.2	0.2-0.4	0.4-0.8	0.8-1.6	>1.6			
000786C0A7	Semi-arid	0.3095	45	5897.07	131.046	43	2	0	0	0	0.0017	0	0
0007B11508	Semi-arid	0.2646	1806	381389	211.179	1110	547	140	9	0	0.1072	0.0113	0.0007
0007B116F9	Semi-arid	0.2673	1708	389476	228.03	996	534	157	20	1	0.1095	0.0184	0.0027
0007DE091B	Semi-arid	0.3084	52	6619.33	127.295	50	2	0	0	0	0.0019	0	0
10002530249	Semi-arid	0.3016	329	54829	166.653	251	72	6	0	0	0.0154	0.0005	0
2530351	Semi-arid	0.2704	1567	331214	211.368	974	459	131	3	0	0.0931	0.0114	0
0007C7AFE8	Temperate	0.2306	2858	650189	227.498	1643	933	253	29	0	0.1828	0.0296	0.0020
0007C7B153	Temperate	0.2305	3358	756725	225.35	1853	1179	320	6	0	0.2128	0.0249	0
0007C7B720	Temperate	0.2658	1506	332785	220.972	905	459	132	10	0	0.0936	0.0137	0
0007C7C1A6	Temperate	0.2475	2381	533696	224.148	1333	820	223	5	0	0.1501	0.0188	0
7C7B6DB	Temperate	0.2442	2329	532387	228.59	1327	753	231	18	0	0.1497	0.0240	0.0014
956000004913012	Temperate	0.2449	2740	605724	221.067	1529	968	238	5	0	0.1703	0.0172	0

Table S3: Ninu individuals identified by faecal DNA analysis in 2021 and 2022 and the numbers of scats successfully genotyped for each individual in each year. Feral cat management is implemented in areas south of the Kiwirrkurra community but not in the north-eastern Ninu colonies. * indicates Ninu that were identified in both 2021 and 2022 (i.e. 'recaptures').

Animal ID	Sex	# scats 2021	# scats 2022	Sub-population ID
Southern colonies				
Ninu1	Male	3		NAM
Ninu2 *	Female	2	2	NAM
Ninu5	Female	13		NAM
Ninu11	Male	4		NAM
Ninu12	Undetermined	1		NAM
Ninu18	Undetermined		1	NAM
Ninu19	Female		1	NAM
Ninu20	Female		1	NAM
Ninu21	Female		3	NAM/NG
Ninu3	Male	5		NG
Ninu4 *	Male	4	8	NG
Ninu6 *	Female	12	2	NG
Ninu22	Male		2	NG
Ninu23	Female		2	WR
Ninu24	Female		1	WW
Ninu25	Male		8	WW
North-eastern colonies				
Ninu7	Female	20		MU
Ninu8	Male	3		MU
Ninu9	Male	9		MU
Ninu10 *	Male	1	6	MU
Ninu13	Undetermined		5	MU
Ninu14	Male		1	MU
Ninu15	Undetermined		2	MU
Ninu16	Female		3	MU
Ninu17	Female		1	MU

Table S4: GO terms and associated genes from association analysis of arid vs. temperate Ninu.

GO term ID	GO term definition	No. of genes	Genes
GO:0048856	anatomical structure development	71	*ABLIM1|ACSBG1|ADGRL3|ARHGEF26|ARID1B|ARNT2|ATP2C2|ATRNL1|BCHE|BNIP3|BTRC|CDH11|CHD2|CNTN5|COL11A1|CSMD3|DIXDC1|DYNC2H1|ECE1|EHD1|EHF|EMX1|ETV1|EYS|FARP1|FMR1|FRAS1|FZR1|GFRA1|GNAI3|GPRC5B|HMGB1|IDE|IDH2|INA|IQCG|KAZN|KITLG|LAMA3|LDB3|LPIN1|MAP2|MBNL3|MCTP2|MECOM|MITF|MYT1L|NLGN1|NME8|NRAP|OSTN|PCSK5|PEAK1|PLD4|PRKN|PRPS1|PTPN2|RBM20|RECK|RXFP2|SLC1A2|ST8SIA2|SYNE1|SYNJ2|TCF7L2|TEX11|TIPARP|TWF1|UTRN|VAX2|ZMYM3*
GO:0006810	transport	51	*ABCG8|AGAP1|ARL14|ARMC8|ATP10B|ATP2C2|ATP8A1|BNIP3|CACNA2D3|CDC42SE1|CP|DENND2A|DLG2|DYNC2H1|EHD1|ELMO1|ERC2|FMR1|FRAS1|GNAI3|GRID1|HMGB1|HTR3B|IDE|IPCEF1|KCNB1|KIF15|LDLRAD3|LRP1B|NHLRC2|NLGN1|OSBPL10|PCSK5|PLD4|PLIN3|PPFIA2|PRKN|RANBP1|RPH3AL|RPSA|SCIN|SLC15A2|SLC1A2|SLC28A3|SLC9A9|SLCO3A1|SV2B|TBC1D21|TMC5|TMEM266|ZDHHC14*
GO:0030154	cell differentiation	48	*ADGRL3|ARHGEF26|ARID1B|BCHE|BNIP3|CDH11|CHD2|COL11A1|CSMD3|DIXDC1|DYNC2H1|EHD1|EHF|EMX1|ETV1|FARP1|FMR1|FZR1|GFRA1|GPRC5B|HMGB1|IDH2|INA|IQCG|KAZN|KITLG|LAMA3|LDB3|MAP2|MECOM|MITF|MYT1L|NLGN1|NME8|NRAP|OSTN|PEAK1|PLD4|PRKN|PTPN2|RXFP2|SPEF2|SYNE1|TBC1D21|TCF7L2|TIPARP|TWF1|VAX2*
GO:0007165	signal transduction	46	*ADGRL3|ARHGEF26|ATRNL1|BNIP3|BTRC|CDC42SE1|COPS8|CRHR2|DIXDC1|DLG2|ELMO1|FHIT|FMR1|FZR1|GFRA1|GNA14|GNAI3|GPRC5B|GRID1|GRM7|HMGB1|HTR3B|IDE|INSYN1|KCNB1|KITLG|LAMA3|LILRA2|LRRFIP2|MAML3|MCTP2|MITF|MUC19|NLGN1|OR4C12|OSTN|PRKAR1B|PTPN2|RANBP1|RECK|RXFP2|SORCS3|TCF7L2|TIAM2|TIPARP|VAX2*
GO:0034641	cellular nitrogen compound metabolic process	29	*ACSBG1|AGPAT4|BBOX1|BTRC|COPS8|DLG2|ECE1|EHF|ETV1|FHIT|FMR1|FZR1|GNAI3|HMGB1|IDE|IDH2|MAML3|MBNL3|MITF|NEU4|PCSK5|PRCC|PRPS1|RBM20|RPSA|ST8SIA2|TCF7L2|TERF2IP|TEX11*

GO term ID	GO term definition	No. of genes	Genes
GO:0022607	cellular component assembly	27	*ABLIM1|ADGRL3|ARHGEF26|ARID1B|CDH11|CNTN5|COPS8|DYNC2H1|EHD1|ERC2|FARP1|IDE|IQCG|KCNB1|LAMA3|LDB3|MITF|NLGN1|NME8|NRAP|PEAK1|PRKN|RPSA|SLC1A2|SYNPO2|TCF7L2|TEX11*
GO:0006464	cellular protein modification process	27	*AGBL1|AGBL4|B3GALNT1|B3GAT1|BTRC|COPS8|CP|DLG2|FZR1|GALNT18|GCSH|GFRA1|KITLG|MECOM|MINDY4|MUC19|PEAK1|PRKAR1B|PRKN|PTPN2|RFFL|SIRT3|ST8SIA2|TIPARP|TWF1|VRK3|ZDHHC14*
GO:0006950	response to stress	23	*AGBL4|ARID1B|ARNT2|ARPP21|B3GAT1|BNIP3|BTRC|CHD2|COPS8|EHD1|EYS|FMR1|FZR1|GNA14|GRM7|HMGB1|IPCEF1|LILRA2|NME8|PRKAR1B|PRKN|SLC1A2|TERF2IP*
GO:0016192	vesicle-mediated transport	21	*ARL14|ARMC8|ATP8A1|CDC42SE1|DENND2A|EHD1|ELMO1|ERC2|GNAI3|HMGB1|KCNB1|KIF15|LDLRAD3|LRP1B|NHLRC2|NLGN1|PLD4|PLIN3|PRKN|RPH3AL|SCIN*
GO:0007267	cell-cell signaling	20	*ARID1B|BTRC|DIXDC1|DLG2|ERC2|GRID1|GRM7|HTR3B|INSYN1|LRRFIP2|MITF|NLGN1|PCSK5|PPFIA2|PRKN|RECK|SLC1A2|SV2B|TCF7L2|VAX2*
GO:0009058	biosynthetic process	20	*ACSBG1|AGPAT4|B3GALNT1|B3GAT1|BBOX1|EHF|ETV1|GALNT18|IDH2|LPIN1|MAML3|MITF|MUC19|PCSK5|PRPS1|RPSA|ST8SIA2|SYNJ2|TERF2IP|ZDHHC14*
GO:0042592	homeostatic process	19	*ABCG8|ATP2C2|BNIP3|CP|EHD1|EMX1|HMGB1|IDE|KCNB1|NHLRC2|NME8|OIT3|PRKAR1B|PRKN|PTPN2|RPH3AL|SLC9A9|TCF7L2|TERF2IP*
GO:0002376	immune system process	19	*AGBL4|ARMC8|ATP8A1|BANK1|BNIP3|BTRC|CHD2|ELMO1|HMGB1|IDE|KIF15|KITLG|LILRA2|MECOM|MITF|MUC19|PLD4|PTPN2|TIPARP*
GO:0050877	nervous system process	18	*ABLIM1|ATP8A1|BCHE|CNTN5|COL11A1|DLG2|EYS|GRM7|HTR3B|INSYN1|NLGN1|OR4C12|PNKD|PRKAR1B|PRKN|RLBP1|SORCS3|VAX2*
GO:0009056	catabolic process	18	*ADAMTS7|ARMC8|BNIP3|BTRC|ECE1|FZR1|GCSH|HMGB1|IDE|LPIN1|NEU4|PLD4|PNKD|PRKN|RFFL|RPSA|SYNJ2|SYNPO2*

GO term ID	GO term definition	No. of genes	Genes
GO:0015031	protein transport	17	*AGAP1|ARL14|DENND2A|DLG2|DYNC2H1|EHD1|FRAS1|HMGB1|IDE|LRP1B|NLGN1|PCSK5|RPH3AL|RPSA|SLC15A2|TBC1D21|ZDHHC14*
GO:0055085	transmembrane transport	15	*ABCG8|ATP2C2|ATP8A1|CACNA2D3|GRID1|HTR3B|KCNB1|SLC15A2|SLC1A2|SLC28A3|SLC9A9|SLCO3A1|SV2B|TMC5|TMEM266*
GO:0007155	cell adhesion	14	*ADGRL3|CDH11|CNTN5|CNTNAP5|DLG2|DPT|HABP2|KITLG|LAMA3|NLGN1|PCDH9|PEAK1|PPFIA2|RPSA*
GO:0007010	cytoskeleton organization	13	*ABLIM1|ELMO1|INA|IQCG|LDB3|MAP2|NLGN1|NRAP|SCIN|SPECC1|SYNE1|TWF1|ZMYM3*
GO:0006629	lipid metabolic process	13	*ACSBG1|AGPAT4|B3GALNT1|DGAT2L6|LPIN1|NEU4|OSBPL10|PLD4|PNLIPRP1|RLBP1|ST8SIA2|SYNJ2|TIPARP*
GO:0044281	small molecule metabolic process	13	*ACSBG1|AGPAT4|DLG2|FHIT|GCSH|GNAI3|IDH2|LPIN1|PLD4|PNKD|PRPS1|RLBP1|SYNJ2*
GO:0000003	reproduction	12	*FETUB|IQCG|KITLG|NME8|PCSK5|RECK|RXFP2|SPEF2|SYNE1|TBC1D21|TEX11|TIPARP*
GO:0040011	locomotion	11	*ADGRL3|DIXDC1|ELMO1|ETV1|GFRA1|HMGB1|IQCG|KITLG|LAMA3|NME8|PEAK1*
GO:0065003	protein-containing complex assembly	11	*COPS8|EHD1|ERC2|IDE|KCNB1|MITF|NLGN1|PRKN|RPSA|SLC1A2|TCF7L2*
GO:0009790	embryo development	11	*ARNT2|COL11A1|DYNC2H1|ECE1|EMX1|FRAS1|KITLG|LAMA3|PCSK5|RECK|VAX2*
GO:0000902	cell morphogenesis	10	*ARHGEF26|CDH11|EMX1|ETV1|FARP1|GFRA1|MAP2|NLGN1|PEAK1|VAX2*
GO:0007049	cell cycle	9	*BTRC|FZR1|GNAI3|KIF15|LPIN1|PRCC|SEPTIN8|TCF7L2|TEX11*
GO:0008219	cell death	9	*BNIP3|ELMO1|FHIT|HMGB1|KAZN|KITLG|MECOM|RELT|RFFL*

GO term ID	GO term definition	No. of genes	Genes
GO:0044403	symbiotic process	9	*BNIP3|BTRC|ELMO1|FMR1|IDE|PCSK5|PRKN|RANBP1|RPSA*
GO:0048870	cell motility	9	*ADGRL3|DIXDC1|ELMO1|HMGB1|IQCG|KITLG|LAMA3|NME8|PEAK1*
GO:0051276	chromosome organization	9	*ARID1B|CHD2|COPS8|HMGB1|MECOM|SIRT3|TCF7L2|TERF2IP|TEX11*
GO:0061024	membrane organization	8	*ATP10B|ATP8A1|BNIP3|ELMO1|GNAI3|LPIN1|NLGN1|SYNJ2*
GO:0005975	carbohydrate metabolic process	7	*B3GALNT1|B3GAT1|IDH2|NEU4|PGGHG|ST8SIA2|SYNJ2*
GO:0006259	DNA metabolic process	6	*COPS8|FZR1|HMGB1|TCF7L2|TERF2IP|TEX11*
GO:0048646	anatomical structure formation involved in morphogenesis	5	*COL11A1|LAMA3|LDB3|NRAP|RECK*
GO:0030198	extracellular matrix organization	5	*ABI3BP|COL11A1|DPT|LAMA3|RECK*
GO:0040007	growth	4	*EMX1|EYS|OSTN|SLC1A2*
GO:0008283	cell population proliferation	4	*BTRC|DIXDC1|EHF|MECOM*
GO:0006397	mRNA processing	4	*FMR1|MBNL3|PRCC|RBM20*
GO:0006605	protein targeting	4	*IDE|NLGN1|RPSA|ZDHHC14*
GO:0051301	cell division	4	*DIXDC1|FZR1|GNAI3|SEPTIN8*
GO:0051604	protein maturation	4	*DYNC2H1|ECE1|GCSH|PCSK5*
GO:0000278	mitotic cell cycle	4	*BTRC|KIF15|LPIN1|PRCC*
GO:0006914	autophagy	4	*BNIP3|HMGB1|PRKN|SYNPO2*

GO term ID	GO term definition	No. of genes	Genes
GO:0007005	mitochondrion organization	3	*BNIP3|PRKN|SIRT3*
GO:0030705	cytoskeleton-dependent intracellular transport	3	*DLG2|DYNC2H1|FMR1*
GO:0006091	generation of precursor metabolites and energy	3	*FAHD1|IDH2|SIRT3*
GO:0034330	cell junction organization	3	*CDH11|LAMA3|PEAK1*
GO:0007568	aging	3	*IDE|SIRT3|SLC1A2*
GO:0021700	developmental maturation	2	*RECK|RXFP2*
GO:0051186	cofactor metabolic process	2	*ACSBG1|IDH2*
GO:0034655	nucleobase-containing compound catabolic process	2	*HMGB1|RPSA*
GO:0003013	circulatory system process	2	*ECE1|PCSK5*
GO:0042254	ribosome biogenesis	2	*BTRC|RPSA*
GO:0006412	translation	1	*RPSA*
GO:0043473	pigmentation	1	*MITF*
GO:0007059	chromosome segregation	1	*TEX11*
GO:0006457	protein folding	1	*GNAI3*
GO:0022618	ribonucleoprotein complex assembly	1	*RPSA*
GO:0006520	cellular amino acid metabolic process	1	*GCSH*

GO term ID	GO term definition	No. of genes	Genes
GO:0006790	sulfur compound metabolic process	1	*ACSBG1*
GO:0007034	vacuolar transport	1	*PRKN*
GO:0006399	tRNA metabolic process	0	
GO:0032196	transposition	0	
GO:0019748	secondary metabolic process	0	
GO:0015979	photosynthesis	0	
GO:0006913	nucleocytoplasmic transport	0	
GO:0007009	plasma membrane organization	0	
GO:0071554	cell wall organization or biogenesis	0	
GO:0071941	nitrogen cycle metabolic process	0	
GO:0140014	mitotic nuclear division	0	

Table S5. Gene ontology (GO) term annotation of gene families significantly expanded, as determined by the CAFE analysis, in the Ninu.

GO term ID	GO term definition	Number of genes	Genes
GO:0006810	transport	155	*AAGAB|ABCG2|ACAP2|ADA|ALOX15|AP2S1|APOOL|ARF1|ATP5F1B|ATP5F1D|ATP5MC2|ATP5ME|ATP5MF|ATP5MG|ATP5PB|ATP6V0C|ATP6V1C1|ATP6V1E1|ATP6V1G2|B2M|CALM1|CAPZA1|CCDC91|CCT5|CD59|CDD|CES1|CHP1|CLNS1A|COX10|COX5B|COX6A1|COX6C|COX7A1|COX7B|COX7C|CRAT|CST3|DNAJA1|DNAJC19|DYNC1I2|EBP1|EIF2S2|EIF2S3|EIF4A3|ENDOU|FABP5|FAM3C|FFAR1|FKBP1A|FKBP4|FXN|GDI1|GLUL|GOT2|HMGA1|HMGB1|HNRNPA1|HSPA8|IL1RN|IMMP2L|LAMTOR1|LAMTOR3|LAPTM4B|LMBRD1|MAGED2|MPC2|MPV17L|MTX2|NDUFA4|NLRP3|NME2|NME4|NMT1|OSBP|P2RX2|PDXK|PHB2|PRDX6|PRELID3B|RAN|RNPS1|RPL12|RPL13|RPL14|RPL17|RPL18|RPL21|RPL22|RPL23|RPL23A|RPL27|RPL27A|RPL28|RPL29|RPL32|RPL35|RPL35A|RPL38|RPL4|RPL5|RPL6|RPL8|RPL9|RPLP0|RPLP1|RPS10|RPS11|RPS12|RPS14|RPS15A|RPS19|RPS2|RPS21|RPS23|RPS24|RPS27|RPS27A|RPS29|RPS4X|RPSA|S100A7|SELENOK|SFTPD|SLC11A1|SLC15A2|SLC19A3|SLC25A21|SLC25A40|SLC25A42|SLC25A6|SLC2A3|SLC3A2|SLPI|SNRPD1|SNRPE|SQSTM1|SRP14|SYP|TAF7|TIMM23|TMEM129|TMEM14A|TMEM14C|TMSB4|TOMM40|TPT1|UBE2D2|UBE2D3|UCP2|UPRT|VDAC1|VDAC3|WASHC3|YWHAQ*
GO:0034641	cellular nitrogen compound metabolic process	147	*ABCG2|ADA|ADAD1|AHCY|AK2|AK3|AK4|AK6|ATIC|ATP5F1B|ATP5F1D|ATP5MC2|ATP5ME|ATP5MF|ATP5MG|ATP5PB|C1QBP|CACTIN|CDD|CES1|CINP|CLNS1A|COX10|COX5B|CPSF6|CRAT|DBI|DIMT1|DPH3|DUT|EBP1|EEF1B|EIF1AX|EIF2S2|EIF2S3|EIF3F|EIF3M|EIF4A3|EIF4H|ENDOU|ENO1|FCF1|FRG1|FXN|GAPDH|GINS2|GNPDA1|HMGA1|HMGB1|HNRNPA1|HSPA8|IMMP2L|LSM4|LSM6|MDC1|ME1|MED7|METTL15|MPC2|MRPL22|MRPL30|MRPL35|MRPS18B|NAXD|NIFK|NME2|NME4|NPM3|NRBF2|ODC1|PARP1|PDXK|PLAAT3|POU5F1|PRORSD1P|PRPS2|PTS|RAMAC|RAN|RNASE1|RNPS1|RPA2|RPL12|RPL13|RPL14|RPL17|RPL18|RPL21|RPL22|RPL23|RPL23A|RPL27|RPL27A|RPL28|RPL29|RPL32|RPL35|RPL35A|RPL38|RPL4|RPL5|RPL6|RPL7L1|RPL8|RPL9|RPLP0|RPLP1|RPS10|RPS11|RPS12|RPS14|RPS15A|RPS19|RPS2|RPS21|RPS23|RPS24|RPS27|RPS27A|RPS29|RPS4X|RPSA|RSL1D1|SCD|SLC19A3|SMNDC1|SNRPA|SNRPD1|SNRPE|SPTLC1|SRD5A1|SULT1A1|TAF7|TARDBP|TFAM|TMEM14C|TPI1|TSR2|UBE2D3|UBE2N|UGDH|UNG|UPRT|WDR4|WDR77|YBX1|ZNF326*
GO:0009058	biosynthetic process	132	*ABHD17C|ADA|AK2|AK3|AK4|AK6|ALOX15|ARF1|ATIC|ATP5F1B|ATP5F1D|ATP5MC2|ATP5ME|ATP5MF|ATP5MG|ATP5PB|C1GALT1|CDD|CDK2AP1|CES1|CINP|CKB|CNBP|COX10|COX5B|DBF4|DBI|DPH3|DUT|EEF1B|EIF1AX|EIF2S2|EIF2S3|EIF3F|EIF3M|EIF4H|ENO1|ESD|FABP5|FUT9|FXN|GAPDH|GATM|GINS2|GLUL|GNPDA1|GOT2|HAS1|HMGA1|HSD11B2|MDH1|ME1|MED7|MPC2|MRPL22|MRPL30|MRPL35|MRPS18B|NAP1L1|NAXD|NDUFAB1|NIFK|NME2|NME4|NMT1|NPM3|NRBF2|ODC1|OSBP|PARP1|PDXK|PIGP|PLAAT3|POU5F1|PRPS2|PTS|RNPS1|RPA2|RPL12|RPL13|RPL14|RPL17|RPL18|RPL21|RPL22|RPL23|RPL23A|RPL27|RPL27A|RPL28|RPL29|RPL32|RPL35|RPL35A|RPL38|RPL4|RPL5|RP*

GO term ID	GO term definition	Number of genes	Genes
			L6|RPL8|RPL9|RPLP0|RPLP1|RPS10|RPS11|RPS12|RPS14|RPS15A|RPS19|RPS2|RPS21|RPS23|RPS24|RPS27|RPS27A|RPS29|RPS4X|RPSA|SCD|SELENOK|SLC11A1|SNRPE|SPTLC1|SRD5A1|TAF7|TARDBP|TFAM|TMEM14C|TPI1|UGDH|UNG|UPRT|YBX1
GO:0009056	catabolic process	89	*ABHD17C|ADA|AHCY|AP2S1|CALM1|CDD|CES1|CHP1|CRAT|CUL1|DUT|EIF4A3|ENO1|ESD|FABP5|FBXO27|FUNDC2|FUT9|GALM|GAPDH|GLUL|GNPDA1|GOT2|HMGB1|HSPA8|LSM4|LSM6|LYPLA1|NRBF2|PLAAT3|PRDX3|PRDX6|RNF13|RNF34|RNPS1|RPL12|RPL13|RPL14|RPL17|RPL18|RPL21|RPL22|RPL23|RPL23A|RPL27|RPL27A|RPL28|RPL29|RPL32|RPL35|RPL35A|RPL38|RPL4|RPL5|RPL6|RPL8|RPL9|RPLP0|RPLP1|RPS10|RPS11|RPS12|RPS14|RPS15A|RPS19|RPS2|RPS21|RPS23|RPS24|RPS27|RPS27A|RPS29|RPS4X|RPSA|SKP1|SLC25A21|SQSTM1|SRD5A1|SULT1A1|TMEM129|TOMM40|TPI1|UBE2C|UBE2D2|UBE2D3|UBE2N|UCHL3|UNG|VDAC1*
GO:0048856	anatomical structure development	84	*ABITRAM|ADA|ADAD1|AK4|ALOX15|ARF1|ATIC|ATP5F1B|ATP5PB|B2M|BMP2|BSG|C1GALT1|CACTIN|CACYBP|CALM1|CCNA2|CCR4|CES1|CKB|COX7B|CUL1|DAZL|DNAJB6|DNAJC19|E2F4|EIF2S2|EIF4A3|ELF5|FABP5|FAM3C|FKBP1A|FKBP4|FMN1|FOXH1|FRG1|FUT9|FXN|GATM|GDI1|GLUL|GOT2|HMGB1|IMMP2L|KCTD15|KRTAP11-1|LLPH|MAD1L1|MAPK6|MEA1|MOG|MYADM|NANOG|NAP1L1|NDRG2|NDUFV2|NME2|NMT1|ODC1|P2RX2|PARP1|PHB2|POU5F1|PRDX3|PTS|RPL22|RPL29|RPL38|RPL7L1|RPS14|RPS19|RPS4X|S100A7|SFTPD|SRD5A1|TMEM14C|TPI1|TWF1|UGDH|UNG|UPRT|VDAC1|WDR77|YWHAQ*
GO:0022607	cellular component assembly	81	*AIF1L|AP2S1|ARF1|ATP5F1D|B2M|BIRC5|C1QBP|CAPZA1|CD59|CDD|CENPA|CHP1|CLNS1A|COX10|COX7A1|CPSF6|CUL1|DYNC1I2|E2F4|EIF2S2|EIF2S3|EIF3F|EIF3M|EIF4H|EPPIN|FKBP1A|FKBP4|FXN|GATAD1|GBP1|GLUL|HAS1|HMGA1|IMMP2L|KCTD15|LSM4|ME1|MRPL22|MT-ND6|NAP1L1|NDUFA11|NDUFA12|NDUFA3|NDUFAB1|NDUFAF4|NDUFB4|NDUFB5|NDUFV2|NIFK|NLRP3|NME2|OCLN|P2RX2|PARP1|POMP|RAMAC|RPA2|RPL12|RPL23A|RPL38|RPL5|RPL6|RPLP0|RPS10|RPS14|RPS19|RPS23|RPS27|RPS27A|RPSA|SKP1|SNRPD1|SNRPE|SQSTM1|TAF7|TFAM|UBE2C|UGDH|UQCRB|WASHC3|WDR77*
GO:0006950	response to stress	80	*ADA|ADGRE2|AHCY|AK3|AK4|ALOX15|B2M|BMP2|C1QBP|CACTIN|CAPZA1|CCL4|CCNA2|CCR4|CD59|CINP|CST3|CUL1|CYCS|DNAJA1|E2F4|EPPIN|FBXO27|FXN|GAPDH|GBP1|GINS2|GLUL|HIGD1A|HLA-A|HLA-DQB1|HMGA1|HMGB1|HNRNPA1|HSD11B2|HSPA8|IL1RN|IMMP2L|MDC1|MT-ND6|MYL12A|NDUFA12|NDUFB4|NLRP3|NME2|NRBF2|OAS1|OSER1|P2RX2|PARP1|PDK4|PHB2|POU5F1|PRDX3|PRDX6|RNASE1|RNF34|RPA2|RPS19|RPS27A|S100A7|SD|SELENOK|SFTPD|SHISA5|SKP1|SLC11A1|SLPI|SQSTM1|SRD5A1|SUV39H2|TARDBP|TIPRL|TMEM129|UBE2D3|UBE2N|UCP2|UNG|VDAC1|VDAC3*

GO term ID	GO term definition	Number of genes	Genes
GO:0007165	signal transduction	79	*ADGRE2|ALOX15|AP2S1|APH1B|ARF1|ARHGAP32|ARL2BP|ATP6V0C|ATP6V1C1|ATP6V1E1|ATP6V1G2|B2M|BIRC5|BMP2|BSG|C1QBP|CALM1|CAPZA1|CCL4|CCNA2|CCR4|CD59|CDD|CHP1|CUL1|DNAJA1|E2F4|ENDOU|FAM3C|FFAR1|FKBP1A|FKBP4|FOXH1|GBP1|GDI1|GNG5|HLA-A|HLA-DQB1|HMGB1|HNRNPA1|HSPA8|IL1RN|IRAK1BP1|LAMTOR3|MAPK6|MOG|NANOG|NDRG2|NLRP3|NME2|OAS1|OR1D2|OR1D5|P2RX2|PARP1|PDK4|PEBP1|PKIG|RAN|RNF34|RPLP0|RPS19|RPS27A|SD|SELENOK|SFTPD|SHISA5|SKP1|SQSTM1|SUB1|TAF7|TIPRL|TMSB4|UBE2D2|UBE2D3|UBE2N|VN1R4|YBX1|YWHAQ*
GO:0065003	protein-containing complex assembly	74	*AP2S1|ARF1|ATP5F1D|B2M|C1QBP|CAPZA1|CD59|CDD|CENPA|CHP1|CLNS1A|COX10|COX7A1|CPSF6|CUL1|EIF2S2|EIF2S3|EIF3F|EIF3M|EIF4H|EPPIN|FKBP1A|FKBP4|GBP1|GLUL|HMGA1|IMMP2L|KCTD15|LSM4|ME1|MRPL22|MT-ND6|NAP1L1|NDUFA11|NDUFA12|NDUFA3|NDUFAB1|NDUFAF4|NDUFB4|NDUFB5|NDUFV2|NIFK|NLRP3|NME2|OCLN|P2RX2|PARP1|POMP|RAMAC|RPA2|RPL12|RPL23A|RPL38|RPL5|RPL6|RPLP0|RPS10|RPS14|RPS19|RPS23|RPS27|RPS27A|RPSA|SKP1|SNRPD1|SNRPE|SQSTM1|TAF7|TFAM|UBE2C|UGDH|UQCRB|WASHC3|WDR77*
GO:0044281	small molecule metabolic process	73	*ABCG2|ADA|AHCY|AK2|AK3|AK4|AK6|ALOX15|ATIC|ATP5F1B|ATP5F1D|ATP5MC2|ATP5ME|ATP5MF|ATP5MG|ATP5PB|BSG|CALM1|CDD|CES1|CHP1|CKB|CNBP|COX5B|CRAT|DBI|DUT|ENO1|ESD|FABP5|FUT9|FXN|GALM|GAPDH|GATM|GLUL|GNPDA1|GOT2|HAS1|IDH3A|LMBRD1|LYPLA1|MDH1|ME1|MPC2|NAXD|NDUFAB1|NME2|NME4|NMT1|OAS1|ODC1|OSBP|PDK4|PDXK|PLAAT3|PRPS2|PTS|RAN|RPIA|SCD|SLC19A3|SLC25A21|SLC2A3|SPTLC1|SRD5A1|SULT1A1|TPI1|TXN|UGDH|UNG|UPRT|VDAC1*
GO:0015031	protein transport	72	*AAGAB|AP2S1|ARF1|ATP6V0C|ATP6V1C1|ATP6V1E1|ATP6V1G2|CCDC91|CHP1|CRAT|DNAJC19|EIF4A3|FFAR1|GDI1|HMGB1|HSPA8|IL1RN|IMMP2L|MTX2|NLRP3|PHB2|RAN|RNPS1|RPL12|RPL13|RPL14|RPL17|RPL18|RPL21|RPL22|RPL23|RPL23A|RPL27|RPL27A|RPL28|RPL29|RPL32|RPL35|RPL35A|RPL38|RPL4|RPL5|RPL6|RPL8|RPL9|RPLP0|RPLP1|RPS10|RPS11|RPS12|RPS14|RPS15A|RPS19|RPS2|RPS21|RPS23|RPS24|RPS27|RPS27A|RPS29|RPS4X|RPSA|SLC15A2|SLC25A6|SRP14|TIMM23|TMEM129|TOMM40|UBE2D2|UBE2D3|WASHC3|YWHAQ*
GO:0044403	symbiotic process	67	*AP2S1|ARF1|ATP6V0C|B2M|C1QBP|CALM1|CCL4|CCNA2|CENPA|CUL1|DYNC1I2|EIF3F|EIF4H|GAPDH|HAVCR1|HMGA1|HNRNPA1|HSPA8|LAMTOR5|LMBRD1|MOG|RAN|RPL12|RPL13|RPL14|RPL17|RPL18|RPL21|RPL22|RPL23|RPL23A|RPL27|RPL27A|RPL28|RPL29|RPL32|RPL35|RPL35A|RPL38|RPL4|RPL5|RPL6|RPL8|RPL9|RPLP0|RPLP1|RPS10|RPS11|RPS12|RPS14|RPS15A|RPS19|RPS2|RPS21|RPS23|RPS24|RPS27|RPS27A|RPS29|RPS4X|RPSA|SFTPD|SKP1|SLC25A6|TARDBP|UNG|VDAC1*

GO term ID	GO term definition	Number of genes	Genes
GO:0002376	immune system process	64	*ADA|ADGRE2|AHCY|AP2S1|ARF1|ATP6V0C|B2M|BSG|C1QBP|CACTIN|CAPZA1|CCL4|CCR4|CD59|CDD|CST3|CUL1|DYNC1I2|EBP1|ENDOU|EPPIN|FABP5|FKBP1A|GAPDH|GBP1|HLA-A|HLA-DQB1|HMGB1|HMGN2|HSPA8|IL1RN|IRAK1BP1|LAMTOR1|LAMTOR3|MAD1L1|MOG|NLRP3|NME2|OAS1|PARP1|PDXK|PRDX3|PRDX6|RNASE1|RPL22|RPS14|RPS19|RPS24|RPS27A|S100A7|SELENOK|SFTPD|SKP1|SLC11A1|SLC2A3|SLC3A2|SLPI|SQSTM1|SRP14|TMEM14C|UBE2D2|UBE2D3|UBE2N|UNG*
GO:0006464	cellular protein modification process	54	*ABHD17C|ACP1|BIRC5|BMP2|C1GALT1|CALM1|CCNA2|CCNG1|CST3|CUL1|CYCS|DBI|DPH3|EIF3F|FBXO27|FKBP1A|FKBP4|FUT9|FXN|GAPDH|GLUL|HECTD2|LAMTOR3|LYPLA1|MAPK6|MED7|NDUFAB1|NLRP3|NME2|NMT1|PARP1|PDK4|PEBP1|PIGP|PIN4|PRDX3|RNF13|RNF34|RNFT1|RPS27A|SD|SELENOK|SHISA5|SKP1|SQSTM1|SUMO2|SUV39H2|TMEM129|TWF1|UBE2C|UBE2D2|UBE2D3|UBE2N|UCHL3*
GO:0006605	protein targeting	50	*CRAT|DNAJC19|HSPA8|IMMP2L|RPL12|RPL13|RPL14|RPL17|RPL18|RPL21|RPL22|RPL23|RPL23A|RPL27|RPL27A|RPL28|RPL29|RPL32|RPL35|RPL35A|RPL38|RPL4|RPL5|RPL6|RPL8|RPL9|RPLP0|RPLP1|RPS10|RPS11|RPS12|RPS14|RPS15A|RPS19|RPS2|RPS21|RPS23|RPS24|RPS27|RPS27A|RPS29|RPS4X|RPSA|SLC25A6|SRP14|TIMM23|TOMM40|UBE2D2|UBE2D3|YWHAQ*
GO:0006412	translation	50	*EEF1B|EIF1AX|EIF2S2|EIF2S3|EIF3F|EIF3M|EIF4H|MRPL22|MRPL30|MRPL35|MRPS18B|RPL12|RPL13|RPL14|RPL17|RPL18|RPL21|RPL22|RPL23|RPL23A|RPL27|RPL27A|RPL28|RPL29|RPL32|RPL35|RPL35A|RPL38|RPL4|RPL5|RPL6|RPL8|RPL9|RPLP0|RPLP1|RPS10|RPS11|RPS12|RPS14|RPS15A|RPS19|RPS2|RPS21|RPS23|RPS24|RPS27|RPS27A|RPS29|RPS4X|RPSA*
GO:0034655	nucleobase-containing compound catabolic process	49	*ADA|AHCY|CDD|DUT|EIF4A3|HMGB1|LSM4|LSM6|RNPS1|RPL12|RPL13|RPL14|RPL17|RPL18|RPL21|RPL22|RPL23|RPL23A|RPL27|RPL27A|RPL28|RPL29|RPL32|RPL35|RPL35A|RPL38|RPL4|RPL5|RPL6|RPL8|RPL9|RPLP0|RPLP1|RPS10|RPS11|RPS12|RPS14|RPS15A|RPS19|RPS2|RPS21|RPS23|RPS24|RPS27|RPS27A|RPS29|RPS4X|RPSA|UNG*
GO:0030154	cell differentiation	46	*ABITRAM|ADA|ADAD1|ARF1|ATP5F1B|B2M|BMP2|BSG|C1GALT1|CACYBP|CCR4|CES1|DAZL|DNAJB6|E2F4|ELF5|FKBP4|GDI1|HMGB1|KRTAP11-1|LLPH|MAPK6|MEA1|MYADM|NANOG|NAP1L1|NDRG2|NME2|P2RX2|PARP1|POU5F1|PRDX3|RPL22|RPS14|RPS19|RSL1D1|S100A7|SELENOK|SQSTM1|SRD5A1|SUV39H2|TMEM14C|TPM4|TWF1|VDAC1|WDR77*
GO:0055085	transmembrane	43	*ABCG2|ATP5F1B|ATP5F1D|ATP5MC2|ATP5ME|ATP5MF|ATP5MG|ATP5PB|ATP6V0C|ATP6V1C1|ATP*

GO term ID	GO term definition	Number of genes	Genes
	transport		*6V1E1|ATP6V1G2|COX10|COX5B|COX6A1|COX6C|COX7A1|COX7B|COX7C|DNAJC19|EIF2S2|EIF2S3|FKBP1A|HSPA8|MPC2|NDUFA4|P2RX2|PHB2|RPS27A|SLC11A1|SLC15A2|SLC19A3|SLC25A21|SLC25A40|SLC25A42|SLC25A6|SLC2A3|SLC3A2|TIMM23|TOMM40|UCP2|VDAC1|VDAC3*
GO:0016192	vesicle-mediated transport	41	*ACAP2|ALOX15|AP2S1|APOOL|ARF1|ATP6V0C|B2M|CALM1|CAPZA1|CCDC91|CD59|CDD|CES1|CHP1|CST3|DYNC1I2|EBP1|ENDOU|FABP5|FAM3C|GDI1|HMGB1|HSPA8|LAMTOR1|LAMTOR3|LAPTM4B|MAGED2|NME2|PDXK|PRDX6|RPS27A|S100A7|SFTPD|SLC11A1|SLC2A3|SLPI|SQSTM1|SRP14|SYP|TMSB4|WASHC3*
GO:0042592	homeostatic process	39	*ABCG2|ARF1|ATP5F1B|ATP6V0C|ATP6V1C1|ATP6V1E1|ATP6V1G2|B2M|CALM1|CCR4|CHP1|CKB|CLNS1A|CUL1|E2F4|FABP5|FFAR1|FXN|HMGB1|LAMTOR1|NME2|OAS1|P2RX2|PARP1|PDK4|PRDX3|PRDX6|RPA2|RPS14|RPS19|RPS24|SELENOK|SFTPD|SKP1|SLC11A1|TMEM14C|TPT1|TXN|UBE2C*
GO:0006091	generation of precursor metabolites and energy	38	*ATP5F1B|ATP5F1D|ATP5MC2|ATP5ME|ATP5MF|ATP5MG|ATP5PB|CALM1|COX10|COX5B|COX6A1|COX6C|COX7A1|COX7B|COX7C|CYCS|ENO1|FXN|GAPDH|GNPDA1|IDH3A|IMMP2L|MDH1|ME1|MT-ND6|NDUFA11|NDUFA12|NDUFA3|NDUFA4|NDUFAB1|NDUFB4|NDUFB5|NDUFV2|RPIA|SDHD|SRD5A1|TPI1|UQCRB*
GO:0007005	mitochondrion organization	37	*APOOL|ATP5F1B|ATP5F1D|ATP5MC2|ATP5ME|ATP5MF|ATP5MG|ATP5PB|COX10|COX7A1|CYCS|DNAJC19|FUNDC2|FXN|IMMP2L|MPV17L|MT-ND6|NDUFA11|NDUFA12|NDUFA3|NDUFAB1|NDUFAF4|NDUFB4|NDUFB5|NDUFV2|NMT1|PARP1|PHB2|PRDX3|SLC25A6|SQSTM1|TFAM|TIMM23|TMEM14A|TOMM40|UQCRB|YWHAQ*
GO:0042254	ribosome biogenesis	34	*C1QBP|DIMT1|EBP1|EIF4A3|FCF1|FRG1|GLUL|LSM6|METTL15|MRPL22|NPM3|RAN|RIOX2|RPL12|RPL14|RPL23A|RPL27|RPL35|RPL35A|RPL38|RPL5|RPL6|RPL7L1|RPLP0|RPS10|RPS14|RPS19|RPS2|RPS21|RPS24|RPS27|RPSA|RSL1D1|TSR2*
GO:0007049	cell cycle	28	*ARF1|BIRC5|CCNA2|CCNG1|CDK2AP1|CENPA|CINP|CKS2|CUL1|DAZL|DBF4|DYNC1I2|DYNLT3|E2F4|LAMTOR1|LAMTOR3|LAMTOR5|MAD1L1|MAPK6|MDC1|PHB2|RAN|RPA2|SKA2|SKP1|SPC24|SUV39H2|UBE2C*
GO:0000003	reproduction	27	*ADA|ADAD1|BSG|CCT5|CKS2|DAZL|DNAJA1|DNAJB6|DNAJC19|EIF2S2|EIF4H|ENDOU|FKBP4|GNPDA1|GOT2|HAS1|HSD11B2|IMMP2L|MAGED2|MEA1|OR1D2|PHB2|PRDX3|RPL29|SRD5A1|UPRT|WDR77*
GO:0022618	ribonucleoprotein complex	26	*C1QBP|CLNS1A|CPSF6|EIF2S2|EIF2S3|EIF3F|EIF3M|EIF4H|LSM4|MRPL22|RAMAC|RPL12|RPL23A|RPL38|RPL5|RPL6|RPLP0|RPS10|RPS14|RPS19|RPS23|RPS27|RPSA|SNRPD1|SNRPE|WDR77*

GO term ID	GO term definition	Number of genes	Genes
	assembly		
GO:0061024	membrane organization	22	*AP2S1|APOOL|ARF1|ATP5F1B|ATP5F1D|ATP5MC2|ATP5ME|ATP5MF|ATP5MG|ATP5PB|CD59|CHP1|HSPA8|LAPTM4B|MPV17L|MYADM|NMT1|RPS27A|SYP|TARDBP|TMEM14A|YWHAQ*
GO:0008219	cell death	21	*API5|BIRC5|C1QBP|CASP7|CUL1|CYCS|GAPDH|HMGB1|NLRP3|PARP1|PDCD5|PRDX3|RNF34|SELENOK|SHISA5|SLC25A6|SMNDC1|SQSTM1|TMEM14A|UBE2D3|VDAC1*
GO:0006629	lipid metabolic process	20	*ALOX15|ARF1|ATP5F1B|CES1|CNBP|CRAT|DBI|FABP5|HSD11B2|IL1RN|LYPLA1|NDUFAB1|OSBP|PIGP|PLAAT3|PRDX6|SCD|SPTLC1|SRD5A1|SULT1A1*
GO:0006397	mRNA processing	20	*C1QBP|CACTIN|CLNS1A|CPSF6|EIF4A3|FRG1|HNRNPA1|HSPA8|LSM4|LSM6|RAMAC|RNPS1|SMNDC1|SNRPA|SNRPD1|SNRPE|TARDBP|WDR77|YBX1|ZNF326*
GO:0005975	carbohydrate metabolic process	20	*CALM1|ENO1|FABP5|FUT9|GALM|GAPDH|GNPDA1|GOT2|HAS1|IDH3A|MDH1|ME1|OAS1|PDK4|RPIA|SI|SLC2A3|SLC3A2|TPI1|UGDH*
GO:0051186	cofactor metabolic process	17	*AHCY|ATIC|CALM1|COX10|DBI|FXN|GOT2|LMBRD1|MPC2|NAXD|PDXK|PRDX3|PRDX6|PTS|SCD|SLC2A3|TMEM14C*
GO:0051276	chromosome organization	17	*CCNA2|CENPA|GATAD1|GINS2|HMGA1|HMGB1|MAD1L1|NAP1L1|NPM3|PARP1|PHB2|RAN|RPA2|RPS27A|SKP1|SUV39H2|UBE2N*
GO:0000278	mitotic cell cycle	17	*ARF1|BIRC5|CCNA2|CCNG1|CENPA|CKS2|CUL1|DBF4|DYNC1I2|E2F4|MAD1L1|MDC1|RAN|RPA2|SKA2|SKP1|UBE2C*
GO:0007010	cytoskeleton organization	15	*AIF1L|ARF1|BIRC5|CAPZA1|CENPA|CHP1|DNAJB6|E2F4|FMN1|GAPDH|RAN|TMSB4|TPM4|TWF1|WASHC3*
GO:0009790	embryo development	14	*ADA|BMP2|DNAJB6|EIF2S2|EIF4A3|ELF5|FOXH1|FXN|NANOG|NMT1|POU5F1|RPL38|RPL7L1|UGDH*
GO:0007267	cell-cell signalling	14	*AP2S1|BMP2|CALM1|CCL4|CUL1|FFAR1|HSPA8|IL1RN|NDRG2|P2RX2|RPS27A|SKP1|VDAC1|VDAC3*

GO term ID	GO term definition	Number of genes	Genes
GO:0051301	cell division	13	*ARF1|BIRC5|CCNA2|CCNG1|CENPA|CINP|CKS2|DYNLT3|MAD1L1|RAN|SKA2|SPC24|UBE2C*
GO:0040011	locomotion	12	*ADGRE2|ATP5F1B|BSG|CCL4|CCR4|DNAJA1|HMGB1|IL1RN|OR1D2|RPS19|SFTPD|SLC3A2*
GO:0050877	nervous system process	12	*B2M|BIRC5|DNAJC19|EIF4A3|FXN|GATM|OR1D2|OR1D5|P2RX2|RPL38|VDAC1|VDAC3*
GO:0048646	anatomical structure formation involved in morphogenesis	12	*ADA|ATP5F1B|C1GALT1|ELF5|FKBP1A|FMN1|FOXH1|GLUL|NANOG|POU5F1|RPL7L1|S100A7*
GO:0006259	DNA metabolic process	12	*CINP|GINS2|HMGA1|HMGB1|MDC1|PARP1|RAN|RPA2|RPS27A|UBE2D3|UBE2N|UNG*
GO:0007155	cell adhesion	11	*ADA|ADGRE2|BSG|CCL4|DNAJB6|HAS1|MOG|MYL12A|NME2|PCDH11X|RPSA*
GO:0048870	cell motility	11	*ADGRE2|ATP5F1B|BSG|CCL4|CCR4|DNAJA1|HMGB1|IL1RN|RPS19|SFTPD|SLC3A2*
GO:0006913	nucleocytoplasmic transport	9	*CHP1|EIF4A3|HNRNPA1|PHB2|RAN|RNPS1|RPL23|SNRPD1|SNRPE*
GO:0006457	protein folding	9	*B2M|CCT5|CHORDC1|DNAJA1|DNAJB6|DNAJC19|FKBP1A|FKBP4|HSPA8*
GO:0006790	sulfur compound metabolic process	9	*AHCY|DBI|ESD|FXN|MPC2|SCD|SLC19A3|SULT1A1|UGDH*
GO:0008283	cell population proliferation	9	*BMP2|CKS2|CUL1|EIF2S2|GLUL|PDXK|RPL23A|RPS27|SLC11A1*
GO:0051604	protein maturation	7	*APH1B|CES1|FKBP1A|FXN|IMMP2L|NDUFAB1|PARP1*

GO term ID	GO term definition	Number of genes	Genes
GO:0006914	autophagy	7	*FUNDC2|HMGB1|HSPA8|NRBF2|SQSTM1|TOMM40|VDAC1*
GO:0006520	cellular amino acid metabolic process	6	*AHCY|GLUL|GOT2|ODC1|PTS|SLC25A21*
GO:0003013	circulatory system process	5	*CES1|E2F4|HSD11B2|IMMP2L|P2RX2*
GO:0007059	chromosome segregation	5	*BIRC5|MAD1L1|PHB2|RAN|SKA2*
GO:0007568	aging	4	*ADA|B2M|CACYBP|HMGA1*
GO:0006399	tRNA metabolic process	4	*DPH3|LSM6|PRORSD1P|WDR4*
GO:0140014	mitotic nuclear division	4	*BIRC5|MAD1L1|RAN|UBE2C*
GO:0040007	growth	3	*EIF4H|FMN1|LLPH*
GO:0034330	cell junction organization	3	*CAPZA1|MYADM|OCLN*
GO:0021700	developmental maturation	3	*BMP2|DAZL|SYP*
GO:0030198	extracellular matrix organization	3	*BSG|DNAJB6|HAS1*
GO:0000902	cell morphogenesis	3	*ABITRAM|BSG|LLPH*
GO:0007034	vacuolar transport	2	*CCDC91|HSPA8*

GO term ID	GO term definition	Number of genes	Genes
GO:0030705	cytoskeleton-dependent intracellular transport	1	*HSPA8*

Table S6. Pair-wise sequence similarity (%) between the seven Ninu MHC-I coding sequences.

Seq	Mala-*UM*	Mala-*UD*	Mala-*UB*	Mala-*UE*	Mala-*UC*	Mala-*UA*
Mala-*UM*		45.2	48.7	43.6	45.1	48.5
Mala-*UD*	61.8		63.9	76	98.3	63.3
Mala-*UB*	64	79.1		61	62.6	98.8
Mala-*UE*	60.1	85.5	75.3		76.8	60.7
Mala-*UC*	61.4	99	78.7	85.9		62.1
Mala-*UA*	63.8	78.9	99.3	75.1	78.4	

*Above diagonal: amino acid sequence identity; below diagonal: nucleotide sequence identity

Supplementary Figures

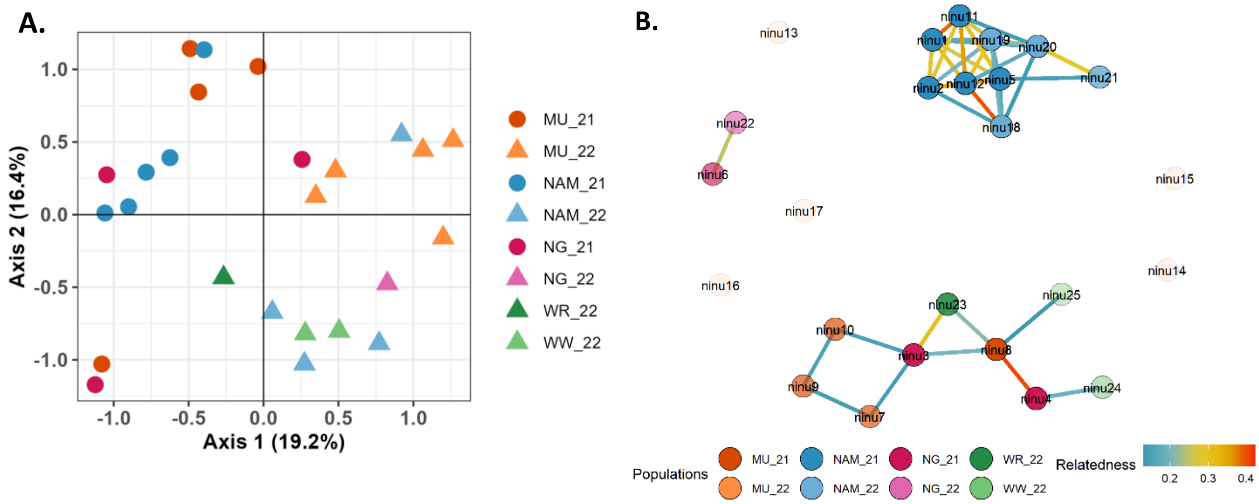

Fig. S1. (A) Principal Coordinates Analysis (PCoA) of all Ninu individuals detected at Kiwirrkurra (2021 – 2022) coloured by sub-population and year of first detection. Circles and triangles indicate Ninu identified in 2021 and 2022, respectively. Note that the MU sub-population is located northeast of the Kiwirrkurra community and remaining subpopulations (NAM, NG, WR and WW) are located south. (B) genetic relatedness matrix amongst all Ninu individuals. Nodes indicate individuals coloured by sub-population and year of first detection. Linkages indicate related individuals (half-sibs and above) coloured by relatedness value.

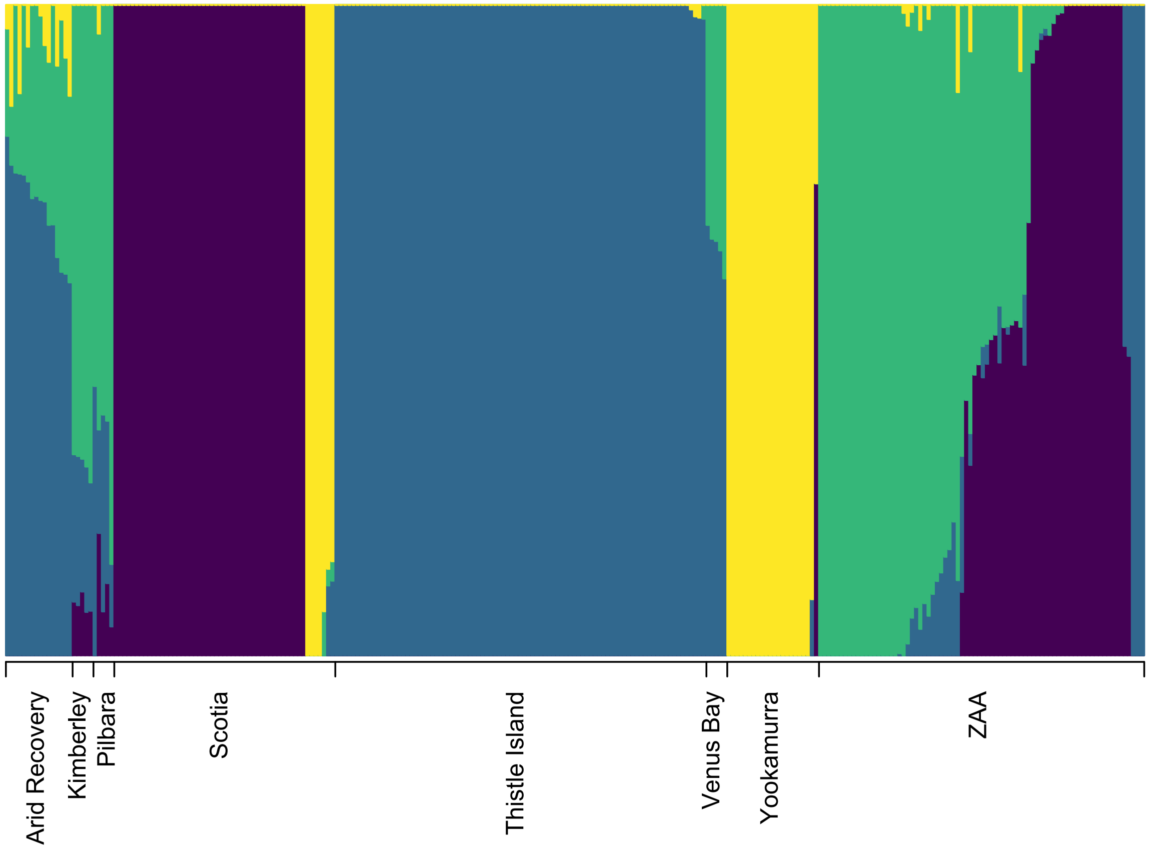

Fig. S2. *fast*STRUCTURE analysis of the Group 1 source populations resulted in an optimum of k = 4 clusters.

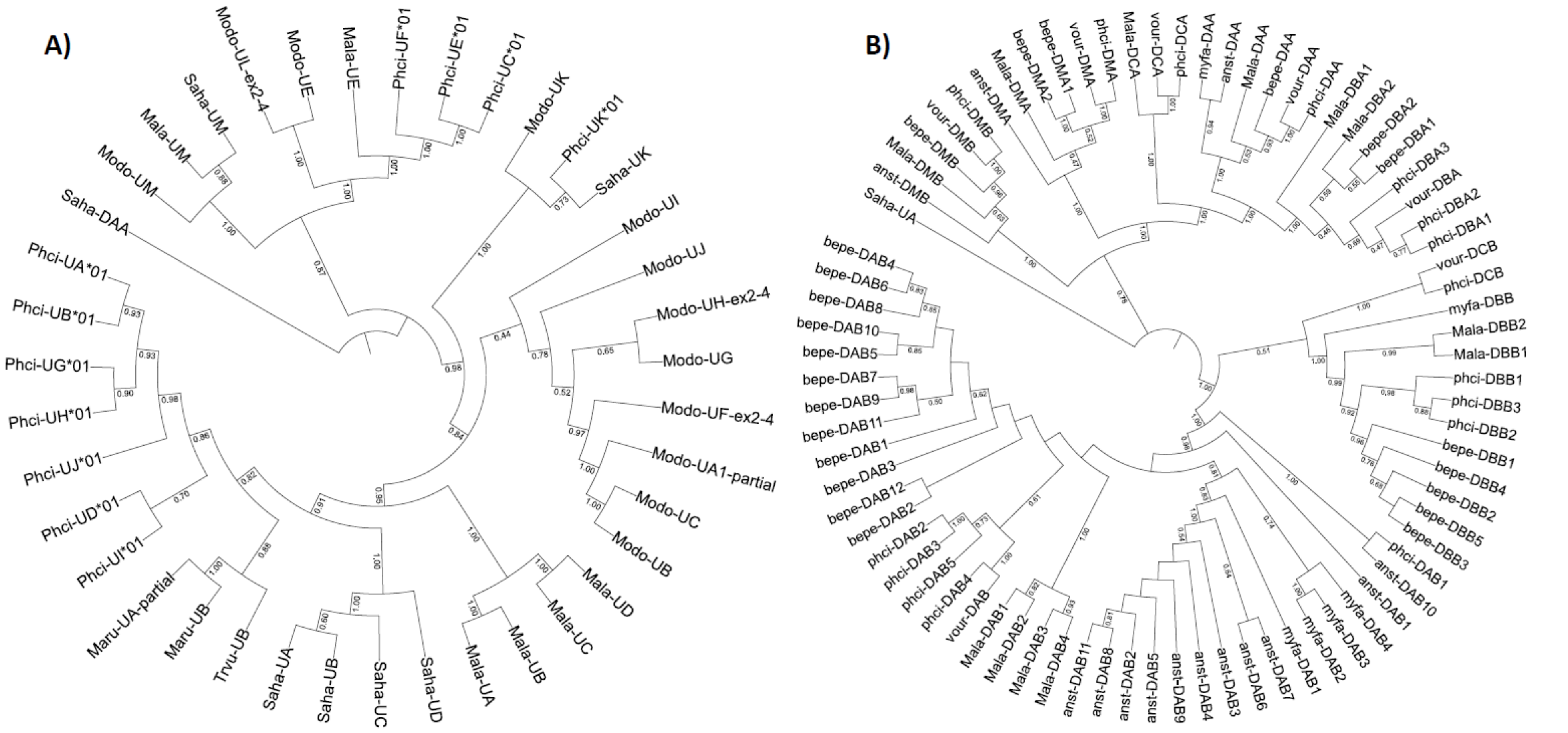

Fig. S3: Phylogenomic analysis of marsupial MHC class I and II genes. Genes were classified into (A) classical (DA, DB and DC) and non-classical (DM) MHC class II and (B) classical (-UA, -UB) and non-classical (-UC, -UE, -UF) MHC-I genes using coding sequences. Gene name prefix denotes species: *Anst* antechinus, *Bepe* Woylie, *Mala* Ninu, *Saha* Tasmanian devil, *Phci* koala, *Vour* wombat.

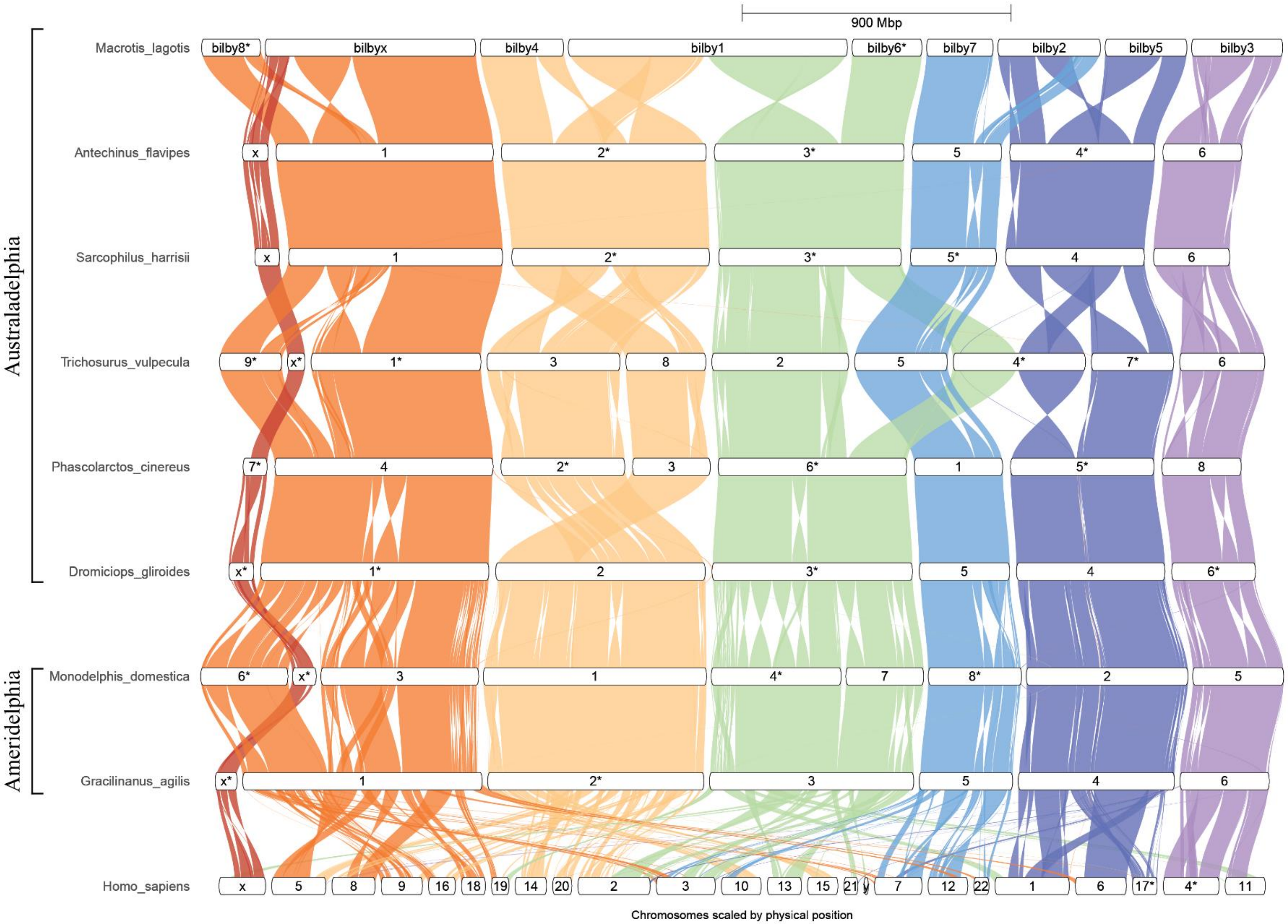

Fig. S4. Genespace synteny plots of the Australadelphia and Ameridelphia marsupials and *Homo sapiens*. Coloured blocks represent chromosome scaffolds for each species. Chromosomes are ordered to maximise visual synteny relative to neighbouring genomes, with the Ninu chromosomes defining the starting order and orientation. Each chromosome is labelled with the species and chromosome number (or X), with an "*" suffix when reversed to minimise inversions.

Supplementary Notes

1 Ninu Genome Sequencing, Assembly and Analysis

1.1 Sample Collection and DNA extraction

Tissue samples (spleen, liver, lymph node, kidney, heart, tongue, ovary, uterus, pouch skin, mammary gland and salivary gland) were harvested from a female captive Ninu at Perth Zoo that was euthanised due to medical reasons in 2018. This individual was 7 years and 11 months of age at death. Tissue samples were collected during post-mortem examination and stored both with and without RNAlater (ThermoFisher, Catalogue AM7020) at -80°C for DNA and RNA extraction respectively. Testis tissue from a single male Ninu at Taronga Zoo that was euthanised for medical reasons in 2021, was flash frozen and stored at -80°C. This individual was 6 years and 7 months of age at death. Additionally, 500uL of peripheral blood from a single male Ninu housed at Dreamworld QLD was collected into RNAprotect animal blood tube (Qiagen, Catalogue 76544) during routine veterinary health checks and stored at -80°C. All samples were collected under opportunistic sampling permits from Perth Zoo and Taronga Zoo, and held under NSW Scientific Licence (SL101204) at the University of Sydney.

1.2 Genome library preparation and sequencing

A high-quality reference genome was produced using a hybrid approach of 10x Genomics linked-read sequencing[1], Pacific Biosciences (PacBio) HiFi sequencing[2] and Dovetail Omni-C™. For the 10x Genomics linked-read sequencing, sample quality control (QC) was assessed by Nanodrop 1000 Spectrophotometer (Thermo Fisher Scientific, USA), Qubit 2.0 Fluorimeter (Thermo Fisher Scientific, USA) and analysed on an 0.75% KBB agarose gel using the Pippin Pulse™, pulse field electrophoresis gel system (Sage Science) at the Ramaciotti Centre for Genomics (RCG; University of New South Wales, Australia). Library preparation was performed by RCG using the 10x Genomics v2 library preparation kit, prior to the 10x Genomics linked-read sequencing on a NovaSeq 6000 S1 flowcell (Illumina) using 150 bp PE reads and obtaining ~57× coverage. Long read sequencing was performed using PacBio HiFi Sequencing with sample QC assessed by confirming DNA fragment size was greater than 40kb with a Femtopulse (Agilent Technologies) and sample purity assessed by absorbance spectrophotometry with a Nanodrop 1000 (Thermo Fisher Scientific) at the Australian Genome Research Facility (AGRF; University of Queensland, Australia). AGRF performed the PacBio HiFi library preparation using the SMRTbell™ Express Template Prep Kit 2.0 (PacBio Catalogue 101-853-100). Sequencing was performed on the Pacific Biosciences Sequel II system across two SMRT Cells in circular consensus sequencing (CCS) mode obtaining ~10× coverage. For Dovetail Omni-C™, the RCG undertook the library preparation and sequencing; 20mg of flash-frozen spleen tissue was ground to a fine powder using a mortar and pestle in liquid nitrogen. This ground tissue was input into the Dovetail Genomics Omni-C™ proximity ligation assay (version 1.3), with a modified 1:10 dilution to the digestion enzyme. The proximity ligated DNA was split at the end of stage 3 into two 150ng aliquots and taken through the final library prep stages. The library size distribution and concentration were assessed using TapeStation™ (Agilent Technologies, California, USA) and Qubit™ (Thermo Fisher Scientific, Massachusetts, USA). The two libraries were pooled and sequenced on the NovaSeq 6000™ (Illumina, California, USA), SP 150bp PE format (RCG).

1.3 Genome assembly

HiFi reads were generated using the CCS algorithm in SMRT Link v9.0.0.92188 and assembled using PacBio's Improved Phased Assembler (IPA) v1.1.2. Purge_dups v1.2.3[3] was used to remove haplotigs and contig overlaps from both the primary and alternate assemblies (Fig. S5). An interleaved linked reads file was created from the raw 10x Genomics reads using

Long Ranger v2.2.2[4] and aligned to the draft assembly with Burrows–Wheeler Aligner (BWA) mem v0.7.17-r1188[5]. The output was sorted using samtools v1.9[6] and scaffolding was performed using ARCS v1.1.1[7] and LINKS v1.8.7[8] with the *-D* option to estimate gap sizes. PBJelly v15.8.24[9] was used for gap filling the scaffolded assembly with default parameters and Pilon v1.20[10] was used to polish the final assembly using the 10x reverse reads that were quality trimmed (trimming parameters: ftl=10 trimq=20 qtrim=rl) using BBDuk v37.98[11]. Vector contamination, low quality scaffolds and remaining false duplications were removed using Diploidocus "dipcycle"[12] with the HiFi reads used for depth analysis and the trimmed 10x PE reads used for kmer analysis. Scaffolds flagged as repeats were put aside and the core genome prepared for HiC scaffolding. First, GapSpanner v0.1.1[13] (https://github.com/slimsuite/gapspanner) was used to reassemble (Flye v2.8.2,[14]) and gap-fill core scaffolds using HiFi reads spanning assembly gaps. Gap-filled scaffolds were then subject to error correction with HyPo v1.0.3[15] (KMC v3.1.1,[16]). For this, HiFi long reads were mapped using minimap v2.17[17] and trimmed 10x short reads with (BWA) mem v0.7.17-r1188[5], before running HyPo with 39X coverage and a 3.6 Gb genome size. Scaffolding based on OmniC data was carried out with HiRise [Version 2.1.6][18]. The assembly was manually curated by iteratively generating and analysing the OmniC contact map. Contact maps were generated by aligning the OmniC data against the corresponding reference with bwa mem [v 0.7.17-r1188, options -5SP][19]. Ligation junctions were identified and OmniC pairs generated using pairtools [v 0.3.0][20]. Subsequently, we generated a multi-resolution OmniC matrix in binary form with cooler [v 0.8.10][21] and balanced it with hicExplorer [v 3.6][22]. We used HiGlass [v 2.1.11][23] and PretextSuite to visualize the contact maps.

The two largest chromosome scaffolds were too big for some tools, so a version of the genome was also created with each of these scaffolds split into two sub-scaffolds. These splits were made at arbitrary assembly gaps approx. half-way along the chromosome. The repeat scaffolds previously isolated by Diploidocus were added back to the split assembly as additional "debris", and subject to another round of gap-filling and cleanup. First, LR_Gapcloser v20180904[24] was run using the HiFi reads, followed by another round of HyPo error-correction as described, above. Following error correction, all non-chromosome scaffolds were descaffolded into contigs to minimise the risk of misassemblies, and another round of Diploidocus "dipcycle" tidy was performed.

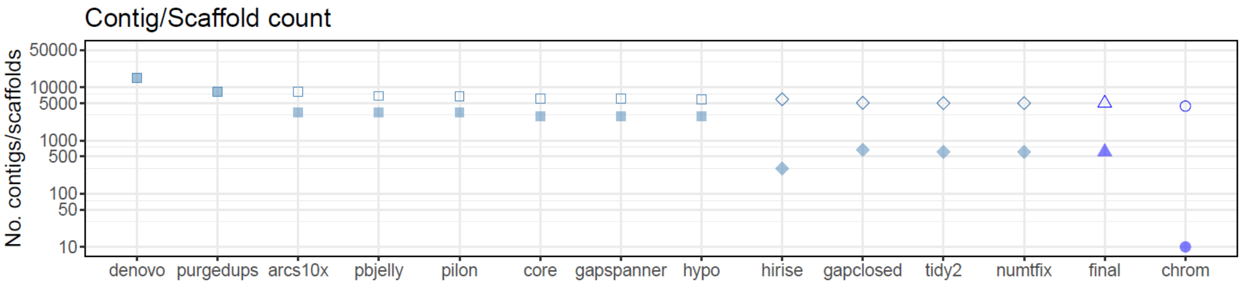

Fig. S5: The total number of scaffolds/contigs (filled symbols) and total number of contigs (outline symbols) per each assembly stage. See Supplementary Excel for full summary statistics on genome assembly versions.

1.4 Mitochondrial genome assembly

NUMTFinder v0.5.2[25] was run against the split HiC-scaffolded assembly using the published Ninu mitochondrial genome, NC_006520.1. No mtDNA contig was identified, but a cluster of putative nuclear mitochondrial fragments (NUMTs) on SCAF_002A was identified consisting of four NUMT fragments. An attempt to assemble the mtDNA was made by extracting mitochondrial reads from the raw HiFi data. GABLAM v2.30.5[26] was used to map NC_006520.1 against the HiFi reads and extract reads that aligned across at least 95% of their length to the Ninu mtDNA. The 17 putative mitochondrial reads extracted were then assembled with Hifiasm v0.16.1[27]. Hifiasm failed to assemble any contigs, so the reads were assembled with Flye v2.9[14], producing a 15,577 linear contig. This was longer than the 15,289 bp RefSeq genome but did not circularise. GABLAM analysis revealed that neither mtDNA sequence wholly contained the other, suggesting that both were partial sequences. The mtDNA Flye assembly was searched against the tidied assembly using NUMTFinder v0.5.2[25] to further characterise the SCAF_002A NUMT region. This included a 15,573 bp NUMT fragment, flanked by simple sequence repeats. Read depth across the region was higher than expected and no mtDNA contig had been assembled, indicating that this was the misincorporation of the mtDNA into the nuclear assembly, as previously reported with long read assemblies[25]. Closer inspection of reads mapped to the NUMT region revealed a probable misassembly upstream of the NUMT, identified by clipping by the vast majority of reads within a few base pairs of the NUMT start and only a single HiFi read supporting the assembly. The remainder of the NUMT regions consisted of NUMT fragments, simple repeats, and assembly gaps with higher or lower than expected sequencing depth. The contig beyond the downstream assembly gap had normal (mean 10.5X) HiFi coverage. HiFi reads mapping to the full NUMT region from the upstream misassembly to the downstream assembly gap (position 61137977-61167704) were extracted with samtools v1.15[6] and reassembled using Hifiasm v0.16.1[27]. A 28,422 bp contig was selected, corresponding to 17,367 bp mtDNA sequence with the first 10,929 bp imperfectly repeated. This included the two simple repeats from the NUMT region (AATACTAT)n and (TAAATTATTA)n. The contig was reverse-complemented to match the RefSeq mtDNA orientation and HiFi reads mapped with minimap v2.22. Average coverage was 65.4X, and so the HiFi mapping was used for both long reads and high-accuracy reads for HyPo v1.0.3 correction. GABLAM v2.30.5 was used to identify overlapping ends and circularise the genome at a position corresponding to the first base of the RefSeq mtDNA. The mitochondrial scaffold, BILBYCHRMT, was annotated using the MitoHiFi pipeline v2 with MitoFinder[28,29] and visualised with MitoZ v2.3[30].

1.5 Manual curation of nuclear genome assembly

Following mtDNA assembly, the NUMT cluster was removed from SCAF_002. Analysis with NUMTFinder v0.5.2 and the assembled mtDNA genome, identified that mtDNA contigs had been misassembled into this scaffold. The 32,110 bp region from the misassembly point to the downstream assembly gap (positions 61137977 to 61170086) was manually excised. Finally, the two split chromosomes scaffolds were rejoined into single scaffolds and the main chromosome scaffolds renamed, producing the final assembly (v1.9).

1.6 Assembly quality assessment

At each step of the assembly process, genome quality was assessed using BUSCO v3.0.2b (mammalia_odb9; n=4104) or BUSCO v5.0.0/v5.2.2/v5.3.0/v5.4.4 (mammalia_odb10; n=9226)[31] BUSCOMP v0.13.0[32], DepthKopy v1.1.0[12] and Merqury v20200313[33] using the trimmed 10x PE reads. BUSCOMP and BBTools v38.73 (RRID:SCR_016968)[11] were used to generate additional assembly statistics. The final 3.656 Gb assembly is comprised of 5,028 contigs (N50 = 1.22 Mb) in 609 scaffolds (N50 = 343.9 Mb), including 9 nuclear chromosomes and a complete mitogenome (Extended Data Table 3). The assembly showed high

completeness with 0.34% gaps, 92.2% Merqury kmer completeness and 93.5% complete mammalian BUSCOs (Extended Data Table 3) (5.1% missing). The BUSCO duplication rate remains quite high at 4.9%, possibly as a consequence of the low HiFi sequencing depth reducing the power of depth-based removal of false duplications. However, the DepthKopy copy number profile of Duplicated BUSCO genes is quite similar to single-copy BUSCO genes (Fig. S6), with a mean copy number of 1.13, suggesting that the majority of the Duplicated BUSCO genes are genuine gene duplications rather than false duplications. 95.6% of the assembly (3.495 Mb) has been assigned to chromosomes, including 8,623 of the 8,625 complete BUSCO genes. Overall sequence accuracy is good, with a Merqury QV score of 40.42 (less than one error per 10 kb across the genome).

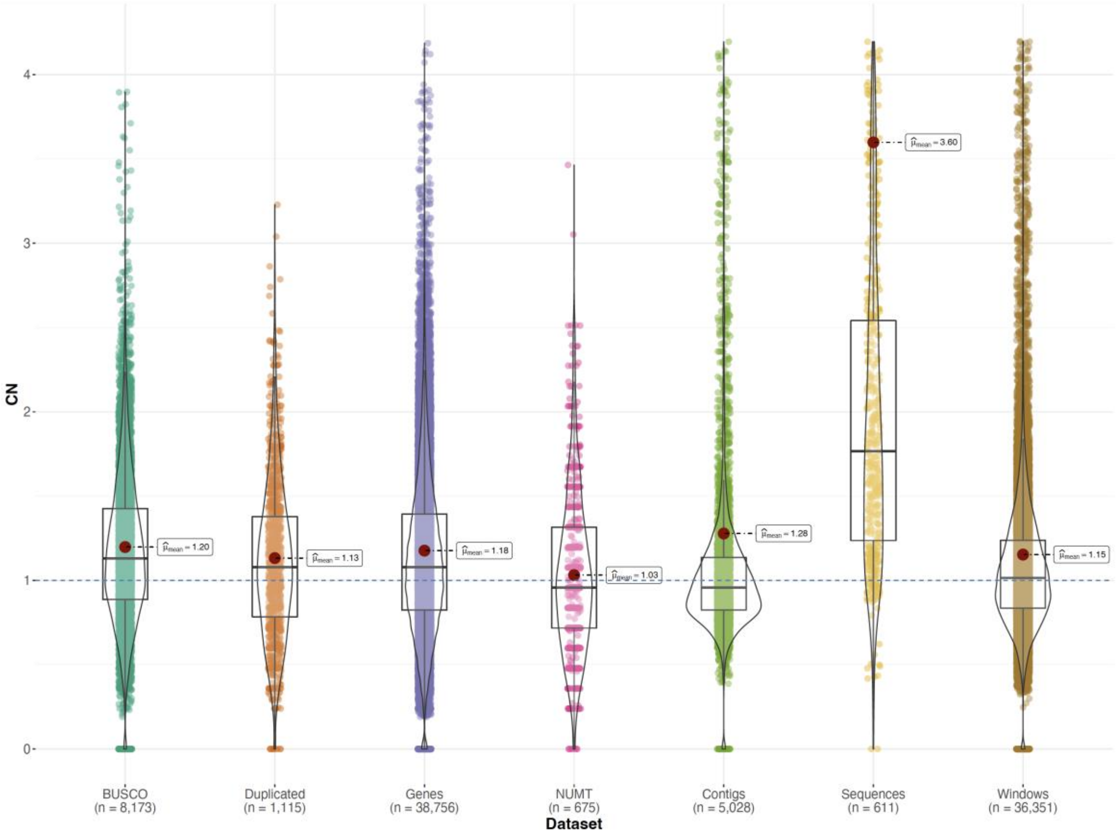

Fig. S6. DepthKopy predicted copy number distribution. Mapped Hi-Fi read depths have been converted into copy number distributions using the modal single-copy read depth from "Complete" BUSCO genes of 8.31X. Plots were generated with ggstatsplot[34] with box plots marking the median and quartiles. Whiskers extend to the most extreme values no further than 1.5 times the inter-quartile range. BUSCO: single-copy complete BUSCO genes; Duplicated: duplicated complete BUSCO genes; Genes: GeMoMa gene models; NUMT: Predicted NUMT fragments; Contigs: individual contigs; Sequences: individual scaffolds (two longest chromosomes split); Windows: 100 kb tiled windows.

1.7 Resequenced genomes

Ninu. Twelve Ninu genomes were resequenced, six individuals (3 males, 3 females; Table S1) were classified as coming from a temperate ancestry (35°S 136°E; island population off the coast of South Australia), and six individuals (3 males, 3 females) were classified as coming from a semi-arid ancestry (26°S 146°E; captive free-range population in south-central Queensland). DNA was extracted from ear biopsy samples that had been stored in 70% ethanol using MagAttract HMW DNA Kit (Qiagen, Catalogue 67563). Sample QC including flurometric assays with Quant-iT (Thermo Fisher Scientific), Qubit (Thermo FisherScientific) and Picogreen (Thermo Fisher Scientific) as well as spectrophotometric analysis with a

Nanodrop (Thermo Fisher Scientific), a TruSeq DNA PCR free library prep (Illumina, San Diego, CA, USA) and WGR was undertaken by the RCG. Samples were sequenced as 150 bp PE reads across a single S2 flowcell on the Illumina NovaSeq 6000 obtaining ~30× coverage per sample.

Yallara. Five Yallara collected between 1895-1931 were sampled (Table S1). All five samples were extracted with a modified protocol from Fulton, Wagner [35] and the DNeasy Blood and Tissue kit (Qiagen Catalogue 69504). DNA extraction was done in a Trace DNA laboratory, taking appropriate precautions to reduce the risk of contamination. One sample (NMVC7091) failed QC and so the remaining four had ThruPLEX DNA (Takara Bio, San Jose, CA) library prep and were sequenced on an Illumina NovaSeq 6000 S1 as 2 x 150 bp paired end reads. Due to low coverage in many of the Yallara samples (Table S1), the two best coverage samples (NHMU1883.10.19.17 and NMVC7087) underwent a Meyer Kircher library prep[36] without sonication due to degraded DNA and Illumina adapter ligation with a dual 8bp index to obtain higher coverage (Table S1).

WGR alignment and variant calling

Resequencing data from the 12 Ninu and 4 Yallara genomes were aligned to version 1.5 (see Supplementary Excel) of the reference genome and variants were called using the DRAGEN Germline platform v3.8.4 (Illumina)[37]. Joint genotyping across all Ninu samples was also performed with DRAGEN Joint Genotyping v3.8.4. Bcftools v1.11[38,39] was used to split multi-allelic variant calls and to left-normalise the variants prior to variant annotation with ANNOVAR v20180416[40]. Genotyping rates were calculated using PLINK v1.90[41]. MultiQC reports for the WGR alignment and variant calling revealed that an average of 1% of reads for the Ninu genomes were unmapped, and 7% were duplicated. Coverage depth ranged from 13.8x to 29.6x (mean = 23.9x, SD = 3.99; Table S1). Joint genotyping of the 12 Ninu WGR samples resulted in 50,177,808 variants with an average genotyping rate of 93.4% (Table S1). Variant annotation identified 114,741 (0.23%) nonsynonymous SNPs across 19,376 genes. Further alignment and variant calling metrics are available in Table S1. The first sequencing run of Yallara genomes had an average of 23% of reads were unmapped and 58% of reads duplicated, with coverage ranging from 0.4x to 7.9x (mean = 2.75x, SD = 3.53; Table S1).

Using SeqPrep2 (https://github.com/jeizenga/SeqPrep2), raw reads from the four Yallara individuals (8 lanes of data in total) were trimmed of their adapters, filtered if they were shorter than 30 bp, and remaining paired-end reads were merged with a minimum overlap of 15 bp (and quality score cutoff for mismatches to be counted in the overlap of 15). The bwa aln algorithm from the Burrows-Wheeler Aligner (BWA) v.0.7.17-r1188[5] was then used to map merged and unmerged reads from the Yallara samples to version 1.9 of the Ninu reference genome. Six male Ninu samples (see Table S1) were also mapped to the Ninu reference genome (v1.9) using bwa aln with default parameters. Resulting BAM files from the same sample but separate runs were merged into one file using samtools merge, and average depth was calculated for each sample using a custom script. BAM files for each sample were separated by chromosome, and HaplotypeCaller from GATK4 v. 4.1.8.1[42] was used to call variants separately. The resulting variant files were filtered individually to remove variants less than a third of the average depth, and more than 3 times the average depth of coverage for that sample. GenomicsDBImport from GATK4 v.4.1.8.1 was used to create a GenomicsDB datastore for each chromosome, before GenotypeGVCFs was used to joint genotype each chromosome separately. Variants were removed in each sample individually if they had less than 1/3 or over 3 times the average depth of coverage for that sample. The samples were merged into a single variant file (containing 1,544,266,668 variants), which was filtered to remove any indels, any sites with map quality of less than 20, all transitions (to account for any DNA damage from the

historic Yallara samples), and any variants called in less than 90% of samples, leaving 511,723 variants remaining.

Given two of the Yallara samples had low mean coverage (see Table S1), two separate PCA analyses were performed. The first PCA was performed on the high coverage samples only which included the two high coverage Yallara samples (9.28× and 12.82× mean coverage) and all six Ninu samples (all over 10× mean coverage). The second PCA was performed on the full dataset which included all Yallara and Ninu samples but with the two low coverage Yallara samples (0.73× and 0.99× mean coverage) projected. Analyses were performed in plink2 v.2.00a2.3LM following linkage pruning and removal of any variants with a minor allele frequency below 0.005 or above 0.99 (leaving 2787 variants) and confirmed the Yallara and Ninu are two separate species. In both PCAs, PC1 splits the Yallara and Ninu explaining 61.6% of the variance in the high coverage dataset and 74.4% in the full dataset with low coverage samples projected (Fig. 1D; Fig, S7). In both analyses PC2 splits the Ninu samples into semi-arid and temperate samples, explaining a further 11% of the variance for the high coverage dataset and 6.92% for the full dataset.

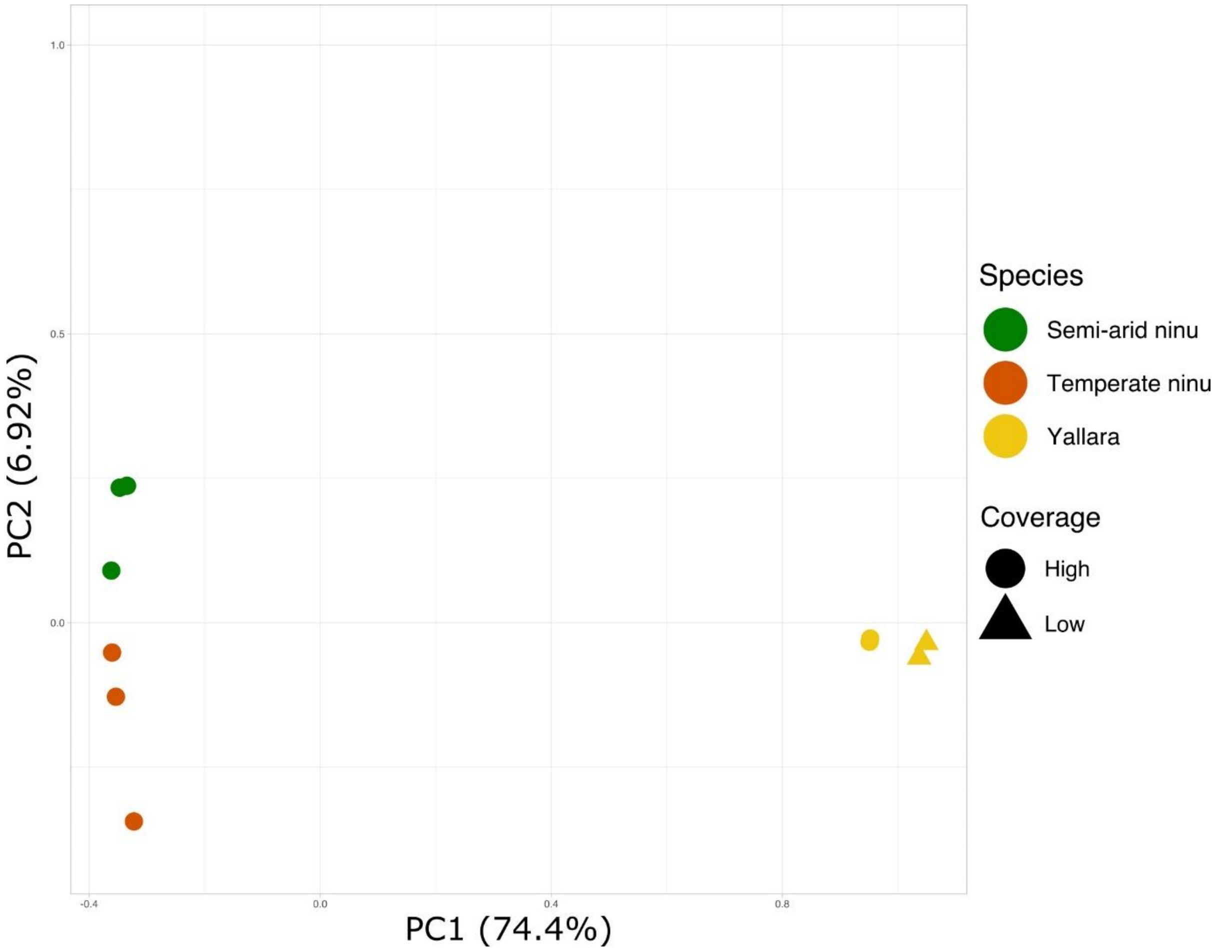

Fig S7. PCA where the low coverage Yallara are projected on to the high coverage individuals.

To confirm the phylogenetic relationships between the Ninu and Yallara, all reads mapping to the mitogenome were extracted from the final BAM files of the high coverage individuals, and a consensus sequence was generated for each sample using a minimum depth of coverage of 3. An alignment was made including all Ninu and Yallara samples, Ninu reference mitogenome (v1.9), and the northern brown bandicoot (*Isoodon macrourus* - NCBI Accession #NC_002746.1) that was used as an outgroup. Maximum Likelihood (ML) phylogenetic analyses were conducted in RAxML v.8.2.12[43], using the GTR+I+G nucleotide substitution model as selected by the Akaike information criteria (AIC) in JModelTest v.2.1.6

(https://github.com/ddarriba/jmodeltest2). Three different ML trees are firstly generated, before branch supports were evaluated on the best scoring tree based on 500 bootstraps of the data. Bayesian analyses were conducted in MrBayes v.3.2.6[44] based on 10 million generations of the data, sampling every 1000 and with the first 10,000 discarded as burn-in. Branch support values from the ML analyses are given in black on major branches as bootstrap support values (as a percentage), while Bayesian posterior probabilities are given in blue (see Fig. 1).

Yallara Reference Genome

We used data from the Yallara with the highest sequencing coverage (NMVC7087) to generate a reference genome. We used the bwa aln algorithm[5] as described above to align reads to the Ninu genome (version 1.9) before obtaining a consensus sequence by using the mpileup function of samtools v.0.1.9 with parameters -I, -B and -u, piping the output to bcftools view v.0.1.19 and finally using the vcf2fq function in vcfutils[6,45]. Following this we used the raw reads to polish the genome assembly using pilon v.1.24[10], we obtained the assembly metrics using stats.sh script within bbmap v.37.98[46]. We then checked the completeness of the Yallara assembly by running BUSCO v.5.4.6 with the vertebrata lineage[31].

1.8 Genome-wide association study

A genome-wide association analysis (GWAS) was performed on the 12 Ninu resequenced genomes to identify allele frequency differences between the six semi-arid and six temperate samples. The reference genome was indexed with Picard v.2.21.9[47] and SAMtools v.1.6[6,38]. The joint-genotyping VCF containing genotype calls for all 12 Ninu was filtered using GATK v.4.2.0.0[48] and VCFtools v.0.1.14[49] to retain only bi-allelic SNPs, with no missing data and a minor allele frequency > 0.05. To mitigate for the small sample size GWAS, we performed three association tests and only retained SNPs that were significant across all three tests, as per Batley *et al.*[50]. Association tests were performed in PLINK v.1.90[41] with either the Chi-square test or Fisher's exact test with a significance level $\alpha = 0.0001$. Weir and Cockerham's F_{ST} outlier test was performed in VCFtools, and SNPs that were > 5 SD away from the mean were retained as candidate SNPs. BEDtools v.2.29.2[51] was used to identify genes containing candidate SNPs using the annotated genome. Unique genes were identified and run through GONet[52] to obtain a network of biological processes with GO term annotation using generic GO slim. GO terms were summarised and visualised with Revigo[53].

Joint genotyping of the 12 Ninu WGR samples resulted in 50,177,808 variants with an average genotyping rate of 93.4%. Variant annotation identified 114,741 (0.23%) nonsynonymous SNPs across 19,376 genes. After filtering we retained a total of 22,299,136 biallelic SNPs for use in our association tests. The Chi-square association test returned 63,868 SNPs, the Fisher's test returned 52,003 SNPs and the F_{ST} outlier test returned 3,858 SNPs. A total of 3,858 SNPs that met our criteria were common across all three association analyses. We identified 339 unique genes. The full list of GO terms and associated genes are provided in Table S4. A summary of GO terms, ranked by their uniqueness is shown in Extended Data Fig. 2.

2 Bilby population genomics

2.1 Historical population size

The mean sequencing coverage for all 296 scaffolds of was estimated for each Ninu and Yallara (first run) resequenced genomes using Mosdepth v0.2.9 [54] with `-n --fast-mode`. We compared the average depth of each scaffold between resequenced Ninu males and females to identify scaffolds putatively belonging to the X chromosome. X chromosome scaffolds are expected to have approximately 50% lower coverage in males than in females when mapping to a female reference genome, as females possess two X chromosomes and males possess one. To ensure sex chromosomes were not included in downstream analyses, we considered scaffolds as belonging to the X chromosome when the average sequencing coverage was 75% lower in males compared to females (Table S7).

Table S7. Scaffolds putatively belonging to the X chromosome (v1.5) determined by the average male scaffold depth being 75% less than the average female scaffold.

Scaffold	Average Depth (Male) / Average Depth (Female)
SCAF_284	0.58
SCAF_180	0.66
SCAF_276	0.68
SCAF_283	0.70
SCAF_139	0.72
SCAF_265	0.73
SCAF_273	0.74
SCAF_255	0.74

Due to the low coverage of the Yallara genomes, we only inferred the historical effective population size of Ninu (n = 12) and Yallara (n = 2) using MSMC and PSMC′ models in MSMC2[55]. Input files were prepared according to the authors' guidelines (https://github.com/stschiff/msmc/blob/master/guide.md) for 14 unphased, diploid genomes. Variant and mask files were generated for each scaffold, per individual, using bcftools v.1.8[39], msmc-tools (https://github.com/stschiff/msmc-tools), and the mean scaffold coverage estimated with Mosdepth.

Five separate analyses were conducted to observe differences across bilby species and populations. These include the combined MSMC analyses for (1) Ninu, (2) Yallara, and subsets of Ninu sampled from (3) temperate and (4) semi-arid regions (Table S8). Additionally, (5) PSMC′ analyses were performed on all 14 individual bilbies. Due to the computational limitations of MSMC, four individuals (eight haplotypes) with the highest mean sequencing coverage were selected for all three Ninu MSMC analyses (Table S8). Two Yallara individuals (four haplotypes) were used as they had sufficient sequencing coverage. Twenty bootstrap replicates were run for each of the two species-level MSMC analyses to provide confidence intervals around the combined estimate.

All demographic analyses used a time interval of `-p 1*3+10*1+1*3` to prevent overfitting of most recent and ancestral estimations. Additionally, the composite likelihood approach was used to run on all pairs of haplotypes, but not across them (`-I`), as the genomes were unphased [55]. We scaled the demographic plots using the estimated mutation rate of the Tasmanian devil (1.17×10^{-9} mutations per site per generation)[45] given the lack of an estimated rate for *Macrotis* spp. We assumed a generation time of two years, based on an average of reported generation times for captive and wild populations of Ninu[56-59]. A parameter sweep was conducted using different mammalian mutation rates and Ninu generation times to evaluate the impact of scaling on demographic estimations.

Table S8. The four individuals with the highest mean sequencing coverage selected for combined MSMC analyses.

Combined MSMC analyses	Individuals used
All Ninu	0007B116F9, 7C7B6DB, 0007C7AFE8, 10002530249
Semi-arid Ninu	0007B11508, 0007B116F9, 10002530249, 2530351
Temperate Ninu	0007C7AFE8, 0007C7B153, 0007C7B720, 0007C7C1A6
Yallara	BMNH1883101917, NMVC7087

Scaling the MSMC plots with the thylacine mutation rate (1.88×10^{-9} mutations per site per generation) estimated the most recent population expansion to have occurred after the onset of the last glacial period (Fig. S8), whereas the same expansion occurred prior to the last glacial period when scaled with the Tasmanian devil mutation rate (1.17×10^{-9} mutations per site per generation; Fig. S6). A direct estimate of the per-generation mutation rate of Ninu would improve the accuracy of demographic inference.

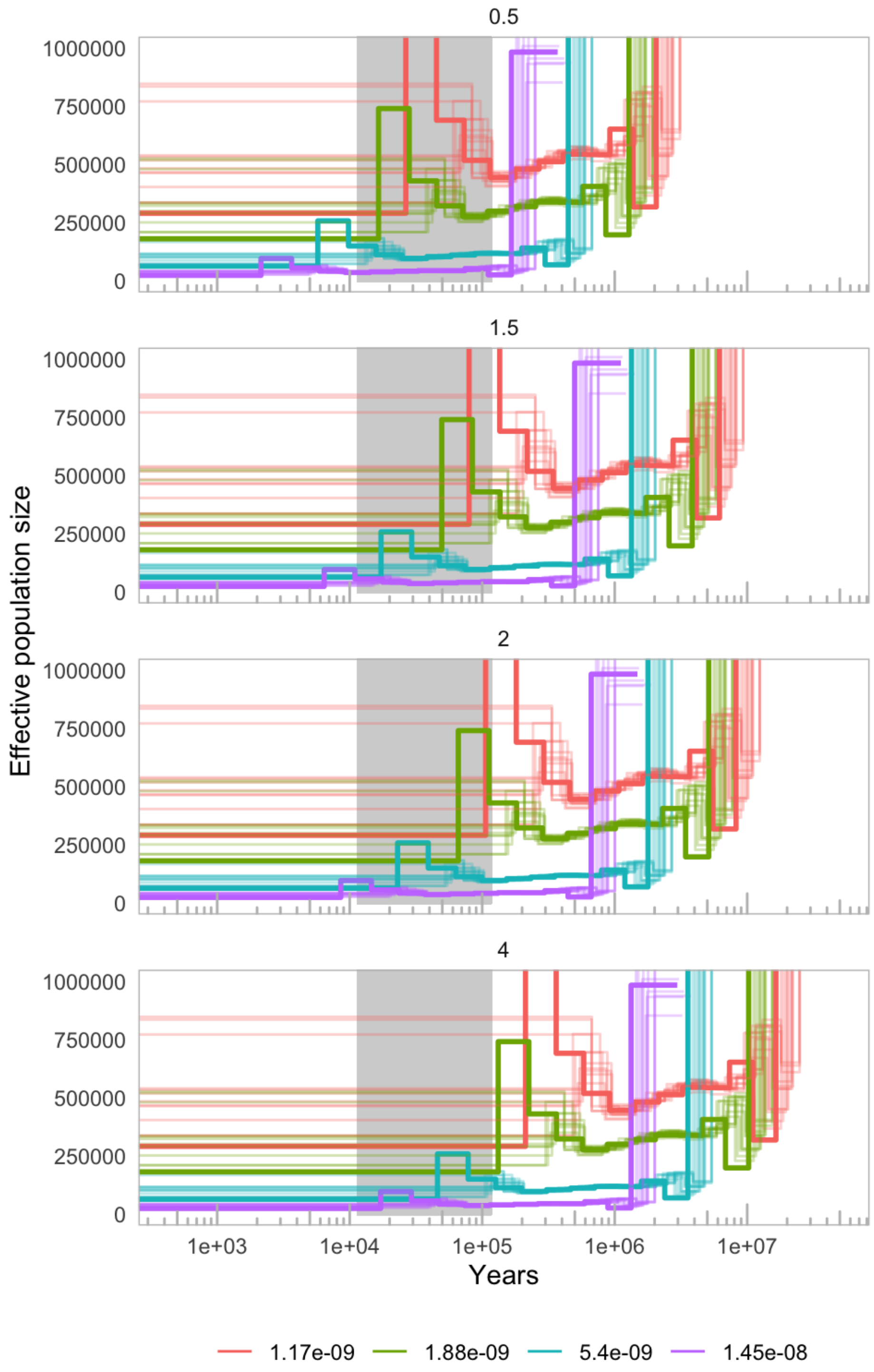

Fig. S8. MSMC of Ninu over mutation rates (coloured lines) and generation times (panels). Bold lines indicate a combined analysis of four Ninu with the highest mean sequencing coverage. Thin lines indicate individual Ninu. Mutation rates are the estimated mutations per site per generation for humans (1.45×10^{-8}) [60], mice (5.4×10^{-9}) [61], Tasmanian devil (1.17×10^{-9}) [45], and thylacine (1.88×10^{-9}) [45].

2.2 Runs of Homozygosity (ROH) analysis

To investigate levels of inbreeding in the 12 resequenced Ninu we characterized runs of homozygosity (ROH) using PLINK v.1.9[41]. Putatively sex-linked scaffolds were removed as

the male heterosomes will affect ROH results. Further, as missing data can have large effects on ROH analyses[62], all missing data were removed from the SNP dataset. After filtering, 29,266,950 SNP remained for the ROH analysis. We ran PLINK using a sliding window size set to 50 SNPs (i.e. *homozyg-window-snp*). Homozygous regions were considered a ROH if they comprised at least 100 kb (i.e. *homozyg-kb*) and comprise at least 100 SNPs (i.e. *homozyg-snp*). To account for genotyping errors, one heterozygous SNP was allowed per window (i.e. *homozyg-window-het*), and five per ROH (i.e. *homozyg-het*; though this is already limited with the previous parameter). To ensure variable SNP coverage across the genome did not bias the detection of ROH, a minimum of one SNP per 50 kb was required to call a ROH (i.e. *homozyg-density*), and the maximum gap allowed between two SNPs (to be considered adjacent) was 200 kb (i.e. *homozyg-gap*). At least 5% of windows were required to contain a given homozygous SNP for it to be considered within a ROH (i.e. *homozyg-window-threshold*).

To check the robustness of our results, we increased the sliding window size and the number of heterozygous SNPs allowed per window to 100 and 2 respectively in separate analyses (i.e. 4 parameter combinations altogether; Table S9). Altering many of the other PLINK parameters (e.g. SNP density; *homozyg-density*) are unlikely to affect the results due to our high SNP-density[62].

ROH were characterized into size classes, whereby longer ROH putatively represent relatively recent instances of inbreeding, while smaller ROH putatively represent inbreeding in the distant past, reflective of the demographic history of the individuals examined[63]. ROH-based inbreeding coefficients (F_{ROH}) were calculated based on the proportion of the genome present in ROH. As not all of the genome is considered in the ROH analysis (e.g. scaffolds shorter than the minimum ROH length are not considered), we calculated the maximal ROH length that the analysis is able to discover (i.e. the genome coverage) using a simulated individual with a completely homozygous genotype following Meyermans, Gorssen [64]. F_{ROH} was calculated for all ROH > 100 kb ($F_{\mathrm{ROH>100kb}}$), 500 kb ($F_{\mathrm{ROH>500kb}}$), and 1 Mb ($F_{\mathrm{ROH>1Mb}}$) to assist comparisons of inbreeding coefficients across studies. Observed heterozygosity was calculated for each individual based on the same SNP dataset using VCFtools.

The six Ninu from the temperate population had lower heterozygosity and generally higher F_{ROH} than those from the semi-arid population (Table S2; Fig. 2C). Three temperate individuals had the highest F_{ROH} (except for $F_{\mathrm{ROH>1Mb}}$) and the lowest heterozygosity. Results were similar when altering the parameters to identify ROH. These results are likely a consequence of the population's relatively small founder size and limited gene flow with other populations (see 2.3 Managed population history). Twenty bilbies were first introduced to the temperate site (Thistle Island) in 1997, with subsequent introductions of 20 bilbies in 1998 and 2000. Since 2000, there has been no gene flow to the island for 35-40 generations.

F_{ROH} values in bilbies from the semi-arid population were relatively diverse, with generally fewer short ROH (e.g. <0.8 Mb) than most temperate individuals (Table S2; Fig. 2C). However, three individuals from the semi-arid population had similar numbers of short ROH to some individuals from the temperate one, as well as numerous long ROH (Fig. S9).

Table S9. ROH values using different PLINK parameter combinations. Changes to the PLINK parameters are specified in the header. FROH values were colour-coded from highest (red) to lowest (blue).

		homozyg-window-het = 2				*homozyg-window-snp = 100*				*homozyg-window-het = 2* *homozyg-window-snp = 100*			
Bilby ID	Source pop	# of ROH	F_{ROH} > 100 kb	F_{ROH} > 500 kb	F_{ROH} > 1 Mb	# of ROH	F_{ROH} > 100 kb	F_{ROH} > 500 kb	F_{ROH} > 1 Mb	# of ROH	F_{ROH} > 100 kb	F_{ROH} > 500 kb	F_{ROH} > 1 Mb
000786C0A7	Semi-arid	54	0.0019	0	0	37	0.0013	0	0	50	0.0018	0	0
0007B11508	Semi-arid	1847	0.1130	0.0130	0.0007	1753	0.1046	0.0113	0.0007	1843	0.1123	0.0127	0.0007
0007B116F9	Semi-arid	1722	0.1144	0.0210	0.0034	1682	0.1075	0.0180	0.0027	1705	0.1136	0.0214	0.0031
0007DE091B	Semi-arid	63	0.0022	0	0	37	0.0014	0	0	58	0.0021	0	0
10002530249	Semi-arid	351	0.0167	0.0005	0	293	0.0138	0.0005	0	340	0.0163	0.0005	0
2530351	Semi-arid	1609	0.0967	0.0109	0	1527	0.0908	0.0113	0	1591	0.0960	0.0120	0
0007C7AFE8	Temperate	2888	0.1910	0.0338	0.0028	2825	0.1804	0.0286	0.0020	2863	0.1894	0.0334	0.0028
0007C7B153	Temperate	3442	0.2210	0.0262	0	3302	0.2090	0.0244	0.0003	3396	0.2197	0.0268	0.0003
0007C7B720	Temperate	1537	0.0978	0.0146	0	1458	0.0914	0.0134	0	1537	0.0974	0.0151	0
0007C7C1A6	Temperate	2467	0.1580	0.0190	0	2327	0.1469	0.0184	0	2436	0.1571	0.0190	0
7C7B6DB	Temperate	2360	0.1566	0.0287	0.0024	2262	0.1463	0.0233	0.0014	2324	0.1550	0.0292	0.0023
956000004913012	Temperate	2797	0.1768	0.0162	0	2686	0.1668	0.0173	0	2770	0.1762	0.0173	0

A)

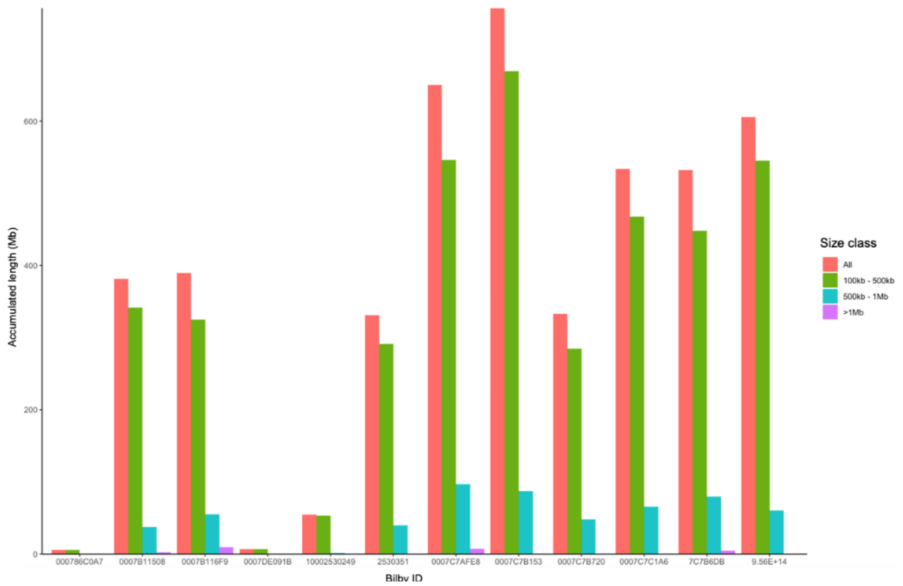

B)

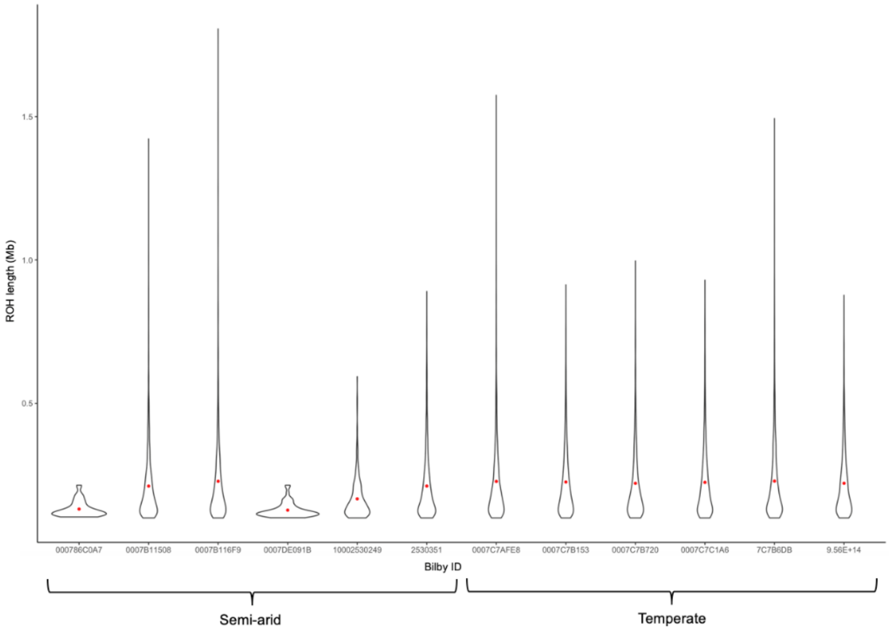

Fig. S9. A) ROH cumulative lengths for different ROH size classes for each of the 12 resequenced Ninu. B) Kernel density (violin) plots of the length of individual ROH in each genome. The red dot represents the mean ROH length.

2.3 Managed Metapopulation History and Study Sites

Ninu were periodically held in zoos for display purposes through the early part of the 20th century. In 1979, to help conserve the species a captive breeding colony was established from 19 founders at the Conservation Commission of the Northern Territory (CCNT), this colony grew to 294 individuals (114M; 111F; 69 unknowns) by 1991[65]. In 1985, Taronga Western Plains Zoo (TWPZ) acquired two pairs of bilbies from CCNT, however one pair escaped so another pair was acquired from CCNT in 1987[65]. Pairs were permitted to breed freely until mid-1989 when space constraints became an issue. Ninu were transferred from TWPZ to other institutions and in 1991 there were six zoos holding 62 captive Ninu representing 5 founders[65]. The Ninu studbook, documenting all records of captive Ninu since 1969, shows 110 Ninu have been sourced from the wild with only 43 individuals thought to be contributing to the population. As of August 2023, the zoo-based population consisted of 142 individuals representing 40 founder animals housed in 26 zoos and wildlife parks. Prior to 2016, captive

Ninu were managed as two separate management units, the WA/NT unit and the QLD unit. These were amalgamated in 2016 after it was agreed that the conservation needs for the species could be best met by ignoring previous taxonomic subdivisions and conserving them as a single taxonomic unit[66]. Ninu from the zoo-based population were intermittently released to fenced reserves, and non-fenced reserves, between 1998 and 2010 (Table S10). Individuals for these populations predominantly came from the WA/NT captive management unit, with 33% coming from Monarto and 47% from Kanyana (Table S10). After the 2015 Greater Bilby Summit[67], a managed metapopulation strategy was developed with new fenced reserves established across the historical Ninu range using the zoo-population (ZAA) and the current reserves as source populations (Table S10).

Table S10. Release location, year, source population and number of individual bilbies released to fenced and non-fenced reserves between 1996 and 2021.

Release location	Fenced	Year	Source population	Individuals released	Status of release location
Perup (WA)		2010	Peron CBC via Kanyana	11	Extinct
Lorna Glen		2007	Peron CBC via Kanyana	21	Extinct
Lorna Glen		2009	Peron CBC via Kanyana	11	
Lorna Glen		2010	Peron CBC via Kanyana	14	
Dryandra Woodlands		1998	Kanyana	5	Extinct
		1999	Kanyana	11	
		2000	Kanyana	17	
		2001	Kanyana	8	
		2002	Kanyana	13	
		2003	Kanyana	13	
		2008	Monarto	8	
		2009	Monarto	2	
		2013	Peron CBC via Kanyana	4	
Arid Recovery	Y	2000	Monarto/Yooka	9	Extant
		2001	Monarto	2	
		2002	Monarto	3	
		2003	Monarto	8	
		2005	Monarto	10	
Venus Bay	N	2001	Monarto	5	Extant

Release location	Fenced	Year	Source population	Individuals released	Status of release location
		2002	Monarto	8	
		2003	Monarto	6	
		2005	Monarto	4	
Thistle Island	N	1997	Monarto	5	Extant
		1998	Monarto	6	
		2000	Monarto	10	
Yookamurra Sanctuary	Y	1996	Winnellie	3	Extant
		1997	Monarto	2	
		1998	Monarto	2	
		1999	Kanyana	2	
		2001	Kanyana	2	
Scotia Sanctuary	Y	1997	Yookamurra	2	Extant
		1998	Yookamurra	5	
		2001	Charleville	9	
Mt Gibson Sanctuary	Y	2016	Scotia	21	Extant
		2017	ZAA /Yookamurra	8 / 7	
		2018	Yookamurra / Thistle Is	5 / 20	
Pilliga Sanctuary	Y	2018	Thistle Is / Scotia	30 / 30	Extant
Mallee Cliffs Sanctuary	Y	2019	Thistle Is / ZAA / Scotia	30 / 10 / 10	Extant
Dubbo Sanctuary	Y	2019	ZAA	16	Extant
		2020	ZAA	2	
Currawinya Sanctuary	Y	2019	ZAA / Thistle Is	17 / 3	Extant
		2020	ZAA	6	
		2021	ZAA	10	

2.4 Metapopulation Sample Collection and Analyses

A total of 363 Ninu sampled between 2011 and 2022 were included in the population genetics analysis. We utilised pre-existing reduced representation sequencing (RRS) (DArTseq, Diversity Arrays Technology, Canberra, Australia) data for 130 individuals[56,68], in addition to

235 ear biopsy samples collected from pre-existing and newly established fenced reserves between 2016 and 2022 (Fig. 3A), including Arid Recovery Reserve (N = 16), Currawinya Sanctuary (N = 70; 35 founders + 35 subsequent generations), Dubbo Sanctuary (N = 64; 18 founders + 46 subsequent generations), Mallee Cliffs (N = 50; founders only), Mt Gibson (N = 26; founders only), Pilliga (N = 36; founders only), Scotia (N = 53), Thistle Island (N = 89), Venus Bay (N = 5), Yookamurra Wildlife Sanctuary breeding enclosure (Yooka1; N = 19) and main enclosure (Yooka2; N = 3). Wild ear biopsy samples were collected from Western Australia (Pilbara, N=5; Kimberley, N = 5)[69], and from deceased individuals in Queensland (Birdsville, N = 1; Currawinya, N = 1).

Variants from the RRS data were called and filtered using previously published methods[68,70]. Briefly, reads were cleaned and demultiplexed using process_radtags in Stacks v.2.61[71,72] and truncated to 68-bp. Trimommatic v.0.39[73] was used to remove adapter contamination and trim low quality reads with the following parameters: ILLUMINACLIP:Truseq3-SE.fa:2:30:10 SLIDINGWINDOW:4:5 LEADING:5. Resulting reads were aligned to the Ninu reference genome (v1.9.fa) using bowtie2 v.2.4.5[74] with output converted and sorted using samtools v.1.6[6] view and samtools sort. Variants were called using Stacks gstacks and populations, outputting one random SNP per locus with a minimum minor allele frequency (MAF) of 0.01 and a minimum call rate across samples of 30%. Additional filtering for minimum average allelic depth (2.5x per allele), allelic coverage difference (≤80%), call rate (≥70%), locus heterozygosity (≤90%) and reproducibility (≥90% matching genotypes between technical replicates) was performed in R v.4.1.2 [75] following Wright *et al.*[70]. Putatively sex-linked SNPs were identified as loci with a heterozygous genotype in at least one female but homozygous in all males and removed. Process radtags and Trimommatic analyses were undertaken on a Pawsey Nimbus Supercomputer (64 vCPUs, 256GB RAM, 3TB attached storage) and all other analysis was on an Amazon Web Services r5.16xlarge cloud machine (65 vCPUs, 512GB RAM, 1TB attached storage, Ubuntu 20.04 LTS). Variant calling and filtering of the RRS data resulted in a total of 9,906 high-confidence SNPs retained for downstream analyses. Reproducibility was high, with an error rate between technical replicates of 1.16%.

To investigate the establishment of the current metapopulation, we separated the population genetic analyses into three groupings (Table 1). Group 1, individuals from the source populations in existence at the start of the 2016 metapopulation expansion (includes Arid Recovery, Scotia, Thistle Island, Venus Bay, Yookamurra1 and 2, ZAA and the wild Pilbara and Kimberley samples); Group 2, translocated individuals that founded the new metapopulation sites (includes Currawinya, Dubbo, Mallee Cliffs, Mt Gibson, Pilliga); and Group 3, offspring of the translocated individuals (includes Currawinya and Dubbo). Note that samples are included in both Group 1 and Group 2 analyses if they were translocated to a new site.

Observed heterozygosity, expected heterozygosity, and allelic richness were calculated using the hierfstat package v.0.5-10[76] in R for all three Groups. Genetic differentiation was visualised using principal coordinate analysis (PCoA) with the dartR package v.1.9.9.1[77]. The inbreeding coefficient F_{IS} was calculated for each population in Group 1 and Group 3 with the diveRsity package v1.9.90 and 1,000 bootstraps to estimate 95% confidence intervals[78]. We did not estimate F_{IS} for Group 2 translocated individuals as the Wahlund effect would likely influence results due to the mixing of diverse source populations at translocated sites[79]. For Groups 1-3, a subset of 5,000 randomly selected SNPs was used to estimate within population mean kinship (MK) by averaging pairwise comparisons estimated with COANCESTRY v.1.0[80] using the triodic maximum likelihood estimator (TrioML) to account for inbreeding.

For the Group 1 source populations, pairwise F_{ST} values (Table S11) were calculated with the StAMPP package v.1.6.3 and 2,000 bootstraps used to estimate the 95% confidence intervals[81].

A subset of 5,000 randomly selected SNPs was used to estimate effective population size (N_E) in NeEstimator v.2.1[82], using the linkage disequilibrium method excluding singleton alleles with corrections[83,84] for bias and missing data. The 95% confidence intervals were taken from the jack-knifed estimate[85]. A fastSTRUCTURE analysis[86] was performed to estimate the number of genetic clusters, K, in the Group 1 source populations, testing K=1-10 clusters with 10,000 iterations for each K. The "chooseK.py" script was used to select the optimum K.

Table S11. Genetic differentiation (F_{ST}) and the 95% CIs between Group 1 source populations.

	Arid Recovery	Kimberley	Pilbara	Scotia	Thistle Island	Venus Bay	Yookamurra 1	Yookamurra 2
Kimberley	0.1361 (0.1286; 0.1441)	NA						
Pilbara	0.1167 (0.1087; 0.1253)	0.0964 (0.0855; 0.1075)	NA					
Scotia	0.1737 (0.1673; 0.1803)	0.1774 (0.1693; 0.1857)	0.1606 (0.1516; 0.1696)	NA				
Thistle Island	0.0760 (0.0720; 0.0801)	0.1155 (0.1078; 0.1236)	0.1155 (0.1078; 0.1236)	0.1712 (0.1653; 0.1770)	NA			
Venus Bay	0.2008 (0.1926; 0.2096)	0.2901 (0.2783; 0.3012)	0.2537 (0.2410; 0.2662)	0.2735 (0.2637; 0.2830)	0.1836 (0.1756; 0.1915)	NA		
Yookamurra 1	0.2249 (0.2157; 0.2335)	0.3067 (0.2946; 0.3189)	0.3023 (0.2894; 0.3168)	0.2390 (0.2302; 0.2477)	0.1863 (0.1784; 0.1940)	0.3943 (0.3818; 0.4064)	NA	
Yookamurra 2	0.1424 (0.1339; 0.1514)	0.2204 (0.2089; 0.2319)	0.1767 (0.1636; 0.1896)	0.1916 (0.1828; 0.2002)	0.1430 (0.1350; 0.1515)	0.3252 (0.3129; 0.3373)	0.1502 (0.1377; 0.1621)	NA
ZAA	0.0891 (0.0849; 0.0932)	0.0863 (0.0805; 0.0922)	0.0699 (0.0632; 0.0768)	0.0791 (0.0756; 0.0825)	0.0904 (0.0868; 0.0937)	0.1881 (0.1800; 0.1961)	0.1681 (0.1610; 0.1754)	0.1319 (0.1240; 0.1400)

The PCoAs showed evidence of population stratification in line with previous findings[56] and what would be expected based on the demographic and translocation history of the Ninu populations (Fig. S10). For example, two of the earliest established sites Yookamurra and Scotia are genetically distinct as shown in previous research, likely due to majority of the founding individuals of Scotia originally being of QLD descent[56]. fastSTRUCTURE analysis of the source populations resulted in four clusters, with all clusters represented to varying degrees in the ZAA metapopulation (Fig. S2). The ZAA metapopulation shows high within-population genetic variation due to the NT/WA and QLD population amalgamation in 2016 and the long-term management of multiple sites across Australia as a single metapopulation (Fig. 3A). ZAA also showed genetic similarity to most other populations due to ZAA being one of the main source populations for other populations nation-wide, and also due to the NT/WA and QLD population amalgamation in 2016 (Table S10 & S11). The wild Pilbara samples show lower within population genetic diversity than other sites and the Pilbara and Kimberley are most genetically similar to the ZAA population (Table S11). The Group 2 translocated sites show genetic structuring reflective of their respective source sites (Fig. S10B). The offspring (Group 3) show evidence of mixing of the translocated founders (Group 2; Fig. S10C).

2.5 MassARRAY SNP Panel Design

We used SNP loci identified from re-mapping raw DArTseq reads (63 – 72 bp) obtained from 12 Ninu populations (Table S12) to the Ninu reference genome (v.1.4.3). A total of 35,039 SNP loci were identified. Filtering of this SNP dataset used custom scripts (see Code Availability), and functions from dartR v.1.9.6[77] and SNPRelate v.0.9.19[87] to obtain high quality, informative SNP loci for the MassARRAY panel design. Data was cleaned by removing genotypes with a total read depth ≤ 10 to ensure high confidence in SNP calls and only selected reads containing a single SNP to ensure that DNA sequence flanking the SNP was stable for primer design. Reproducibility per SNP locus was calculated by comparing SNP genotypes obtained for replicate samples, after which technical replicates were removed from the dataset. A total of 283 tissue samples were used to identify potential SNP markers (Table S12).

Additional filters were applied to select SNP markers for individual identification: a minimum sequence length of ≥ 50 bp, SNP position between 25 and 45 bp, genotype quality score ≥ 20, average read depth between 10 and 200, average genotyping rate per locus ≥ 90%, average genotyping rate per individual ≥ 80%, read coverage difference between reference and alternate SNP between 0.2 and 0.8, reproducibility scores ≥ 95%, read sequence similarity (hamming distance) < 25%, SNP heterozygosity between 0.2 and 0.6 and minimum minor allele frequency ≥ 0.3. Loci not in Hardy-Weinberg Equilibrium and those in Linkage Disequilibrium were removed. Where filtering rendered SNP loci monomorphic across the dataset, these were removed at the conclusion of each filtering step. A total of 74 SNP loci remained available for primer design following filtering.

We calculated the combined Probability of Identity (P_{ID}) across the 74 loci using GENALEX v.6.5 [88] to determine the minimum number of loci required to distinguish unique Ninu. We chose a P_{ID} threshold of 0.0001 equivalent to an exclusion probability > 99.9% as suggested by Waits *et al.*[89]. This analysis indicated 10 markers were required to discriminate related (full-sibling) individuals (P_{IDsibs} = 19).

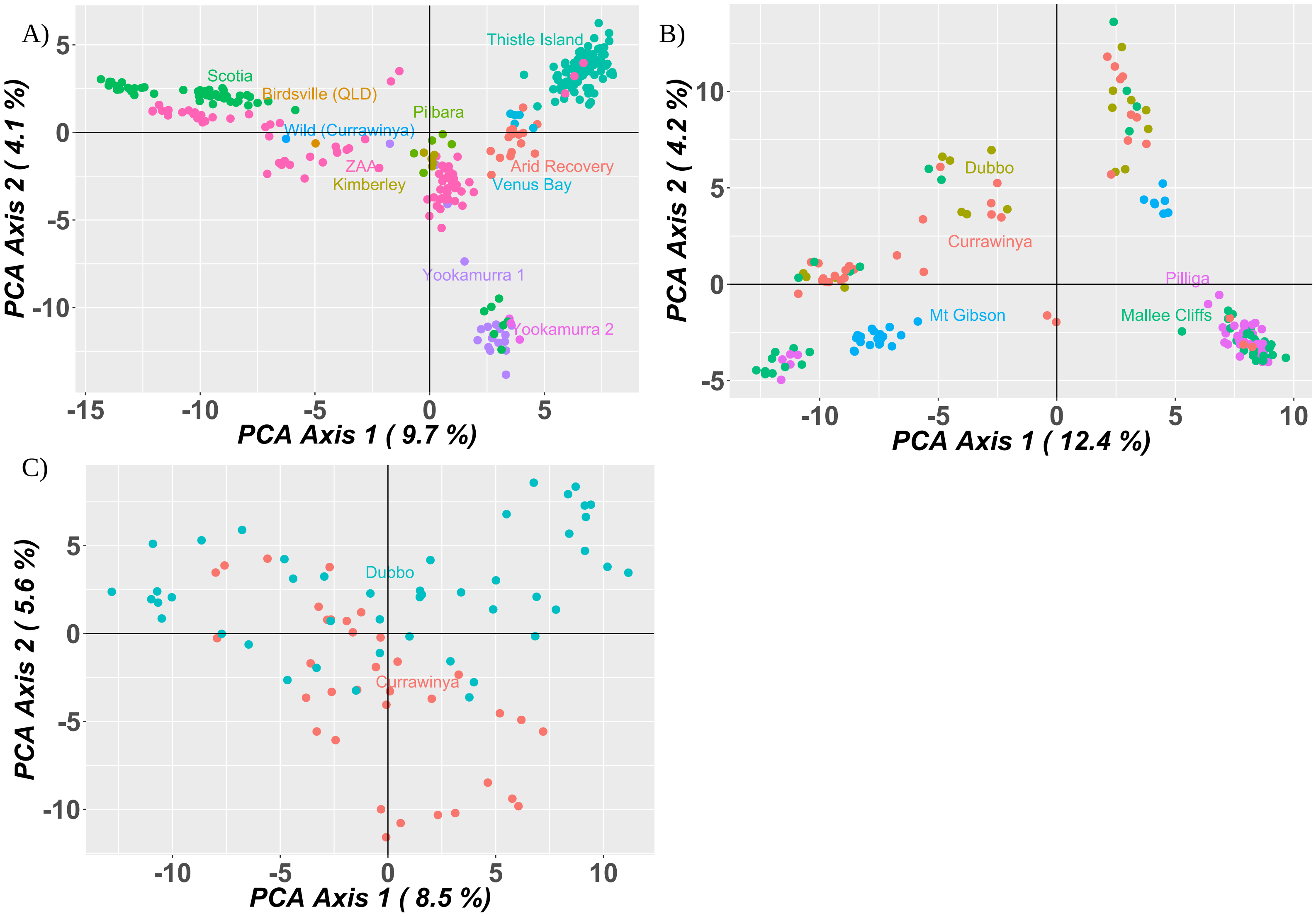

Fig. S10: PCoA from RRS data showing genetic variation of 363 Ninu across 13 populations of the managed metapopulation, separated into A) source populations, B) translocation population founders, and C) translocated population offspring (Table 1).

Table S12. Sample sizes of twelve populations included in the DArTseq dataset used for MassARRAY SNP panel design.

Population	No. of samples
Arid Recovery	16
Currawinya	35
Mallee Cliffs	49
Mt Gibson	26
Pilbara	6
Kimberley	3
Pilliga	36
Scotia	11
Thistle Island	29
Venus Bay	5
Yookamurra 1	20
Yookamurra 2	3
ZAA (captive populations)	44

Using the male Ninu WGR data aligned to the reference genome (version 1.4.3), unmapped Y-chromosome contigs associated with seven marsupial Y genes (*HUWE1*, *KDM5D*, *MECP2*, *RBM10*, *UBE1Y*, *HCFC1* and *HSFY*) were identified through BLAST searches, totalling ~20 kb worth of Y-chromosome sequence. Contigs associated with three genes (*MECP2*, *RBM10* and *UBE1Y*) showed potential for some non-specific binding when BLASTed against the reference genome so were excluded from further analysis. Six primer sets were designed within intronic regions of the remaining four genes (*HUWE1*, *KDM5D*, *HCFC1* and *HSFY*; Table S13). The six markers were first tested on DNA extracted from tissue samples of eight known-sex individuals. PCRs were carried out using the Multiplex PCR Plus Kit (Qiagen, Catalogue 206152). Thermocycling conditions followed a protocol of 15 min at 95°C, then 35 cycles of 30 s at 94°C, 90 s at 60˚C and 60 s at 72°C, with a final extension of 60°C for 30 min. Resultant fragments were run on a 3% agarose gel at 80V and stained with GelRed (Biotium) to be visualised under UV light. Presence of a distinct band indicated amplification of Y-chromosome sequence and therefore assigned males, while absence of a distinct band assigned females (Fig. S11a). The two best markers (KDM5D.2 and HCFC1.2) were then tested on DNA extracted from scat samples of 10 known sex individuals and were found to successfully identify sex from these non-invasive samples (Fig. S11b).

Table S13. Primer information for the 6 Y-linked markers.

Y Marker	Forward Primer (5'-3')	Reverse Primer (5'-3')	Product Size (bp)	Optimal Annealing Temperature (˚C)
HUWE1	ACATGGGCTAAGG GTGAATG	TACTTCCTCGCCTA AATAACAG	170	49.9
KDM5D.1	AGTTGGGATATGG AAACATTG	ATCTCCTGGATTG GCTTCTG	226	49.6
KDM5D.2	TTGTCCCAAATGTT CTAAGC	GTTGGCAATACAG AAAGAGG	155	47.2
HCFC1.1	TTGTTTGTGGAGC AGGAGAG	TTACCCTTCCCTAT TCTTCC	152	48.5
HCFC1.2	ATCCTGCAATTATT GTTTATG	TATGGTTATAAAC TAGCATGTG	132	46
HSFY	TAGGCAATAACAG AGCTGTC	ACTAACATAATGA AAGGTATTC	224	46.2

a) b)

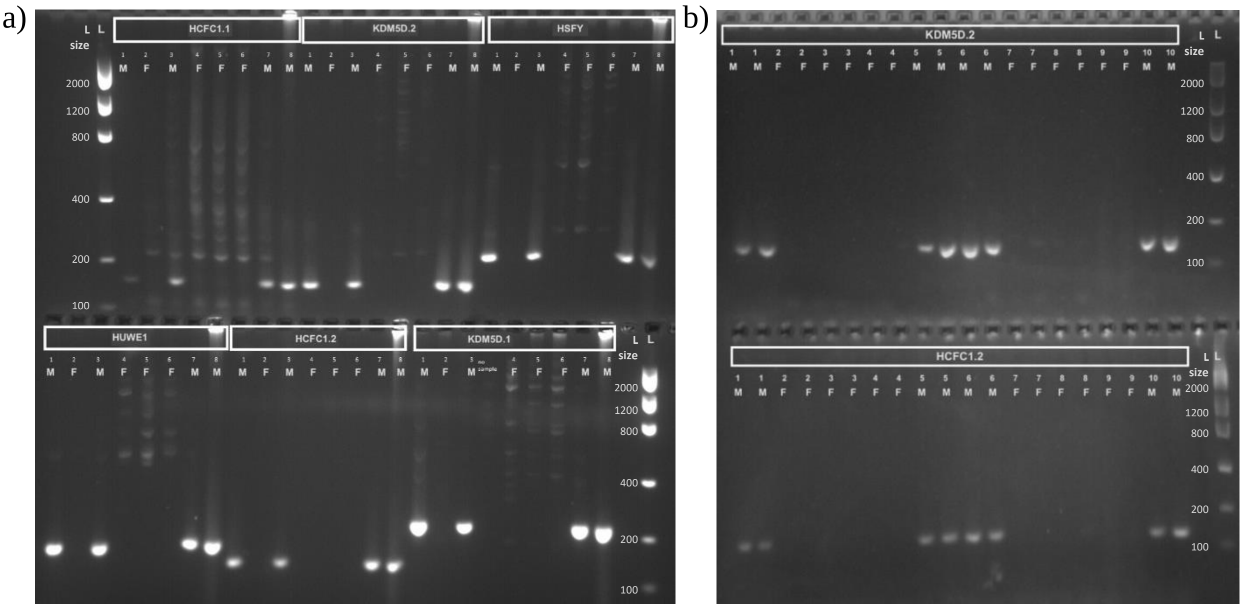

Fig. S11. Gel electrophoresis of the Y-linked markers (*HUWE1*, *KDM5D*.1, *KDM5D*.2, *HCFC1*.1, *HCFC1*.2, *HSFY*) tested in a replicate experiment on DNA extracted from a) tissue samples from Western Australia, and b) scat samples of known sex individuals from Queensland in a single experiment (M = male, F = female). Numbers indicate different individuals. L: Low DNA mass ladder (Invitrogen). Refer to Table S13 for primer information.

We obtained the sequence information for the 74 identified SNP loci from the DArTseq output and combined this with the identified sex-linked markers. Sequences for loci of interest were sent to the Australian Genome Research Facility, Brisbane (AGRF) to undertake *in silico* multiplex assay design using the Assay Design Suite (v2.2, Agena Bioscience, San Diego, CA, USA). Agena MassARRAY accommodates highly multiplexed assay designs of up to 50 markers per panel. Following optimisation of assay design, 42 autosomal SNP and 5 sexing markers were included on the final panel (n = 47 markers total).

The Ninu SNP panel was tested first on a subset of high-quality tissue samples (Mt Gibson Sanctuary founders, N = 17) and faecal samples (scats) obtained from Mt Gibson Sanctuary (N = 26), the Pilbara (N = 10) and the Kimberley (N=10). Genomic DNA was extracted from tissue samples using a standard 'salting out protocol[90] with the addition of 3 µL 10 mg/mL RNase A (Omega Biotek, Catalogue AC118) to the TNES buffer (50 mM Tris, pH 7.5, 400 mM NaCl, 20 mM EDTA, 0.5% SDS) to remove RNA contamination and re-suspension of DNA at the final step in low TE Buffer. Genomic DNA was extracted from scat samples using the Omega Biotek Mag-Bind Stool DNA 96 kit (Omega, USA, Cat No: M4016-01). Starting material for DNA extraction was obtained by briefly soaking and then gently washing scat samples in ~400 µL of SLP buffer[91] to remove sloughed cells from the surface of the scat. Approximately 300 µL of starting material was transferred to extraction tubes and DNA extraction carried out following the manufacturer's instructions. At the final step, DNA was eluted using a 50% dilution of the elution buffer to reduce EDTA carryover and inhibition of downstream analyses. Samples were eluted in 100 µL elution buffer. We subsampled 60 µL of each DNA extract and further concentrated to 30 µL for MassARRAY analysis.

SNP genotyping was carried out on the MassARRAY system (Agena Biosciences, San Diego, CA, USA). Amplification and extension reactions were performed using the iPLEX Gold Reagent Kit (Agena Bioscience, San Diego, CA, USA) according to the manufacturer's protocols using 1 µL of tissue or faecal DNA. Resultant SNP genotypes were identified by mass spectrometry and called using MassARRAY TyperAnalyzer 4.1 software (Agena Bioscience, San Diego, CA, USA) by AGRF. The genotypes identified from MassARRAY analysis were cross-checked with the expected genotypes from DArT data. We included ~10% repeats to ensure consistency across runs and to calculate the genotyping error rate.

Due to the poor performance of seven autosomal and one sex-linked marker in the initial trial, these loci were removed from the SNP panel. The remaining data was quality filtered and genotypes clustered to identify individuals from scats by the R package 'ScatMatch' [92]. We only used samples and loci with $\leqslant$ 30% missing data. The clustering threshold (h) used to group samples by genotypes was set to six that is any scats with less than six SNP differences were assigned to the same group or putative individual. From the trial results, we identified 3 Pilbara Ninu, 5 Kimberley Ninu, and 7 Mt Gibson Ninu from scats. All 17 Mt Gibson founders from tissue samples were identified as unique individuals.

Subsequently, we genotyped faecal samples from the Kiwirrkurra Ninu population case study using 35 autosomal and four sex-linked markers (note preliminary results indicated 19 markers were required to discriminate between related individuals with 99.9% certainty). Data quality filtering and genotype clustering were carried out in the R package 'ScatMatch' [92]. Genotyping success rates at the sample level varied between years (76% in 2021, 49% in 2022) with locus amplification and genotyping error rates following QC also variable (amplification rate: 90% in 2021, 54% in 2022; allele error rate: 0.001 ± 0.000 in 2021, 0.014 ± 0.004 in 2022). Due to the variation in genotyping quality between years we used different QC thresholds retaining only samples and loci with ≤ 20% missing data in 2021 and retaining samples and loci with ≤ 30% missing data in 2022. Clustering thresholds used to group faecal samples by genotype to identify individuals varied also (h = 4 in 2021, h = 6 in 2022). Allowing more allelic mismatches for lower quality genotyping data reduces the impact of over-splitting genotypes, and therefore overestimating numbers of individuals, due to genotyping error.

Once scat samples were clustered (Fig. S12), we identified 12 unique Ninu across two colonies in 2021 (Table S3). In 2022, there appeared to be high turnover of individuals with only four of the 12 individuals from 2021 remaining in the population and detected again by the Kiwirrkurra Rangers. These included three females and one male (Table S3). A further 13 new

individuals were identified, including three individuals detected at two new sites where Ninu activity was not detected in 2021 (Ninu 23, 24, 25). All Ninu activity had shifted away from centres of activity in 2021 despite the collection locations being the same (Fig. 3B). In the case of the four 'recaptured' individuals, each was detected within the same general area they were detected in 2021, albeit with some small-scale movement between years. In 2021, one male individual was detected across two sites (MN & MU) with scats collected 6 weeks and 68km apart, and in 2022, a female was detected across two sites (NG & NAM) with scats collected two days and 37 km apart. The sex ratio appears to vary between collection years with more males represented in scat samples than females in 2021 (4F:7M:1U) and vice versa in 2022 (9F:5M:3U).

In order to undertake comparisons between the Kiwirrkurra Ninu and other locations in the managed metapopulation, we extracted the MassARRAY SNPs from the DArTseq dataset and unique genotypes from the trial, retained samples and loci with ⩽ 30% missing data, and calculated genetic diversity statistics in the hierfstat R package v.0.5-11[76]. Cumulatively, the genetic diversity of the wild Ninu population at Kiwirrkurra is comparable to other wild populations (Extended Data Table 4).

The north-eastern and southern Ninu colonies are located approximately 70 km apart. To assess gene flow amongst populations, we visualised genetic relationships amongst individual Ninu detected in 2021 and 2022 using PCoA in GenAlEx and by calculating pairwise genetic relationships using the gl.grm.network function in dartR[93]. There was no obvious genetic structuring amongst Ninu sub-populations (colonies) identified in PCoA (Fig. S1A), although sample sizes are small, and our genetic markers are not overly sensitive to fine-scale structure. The pairwise genetic relatedness network indicates high genetic relatedness amongst individuals within the NAM sub-population and across collection years and that there are several close relationships (half-sibs and above) between individuals located in the northeastern MU sub-population and the southern NG, WW and WR sub-populations. Taken together, the lack of genetic structure and the movement of related individuals between northeastern and southern sub-populations indicates bilbies appear to be moving freely over the landscape.

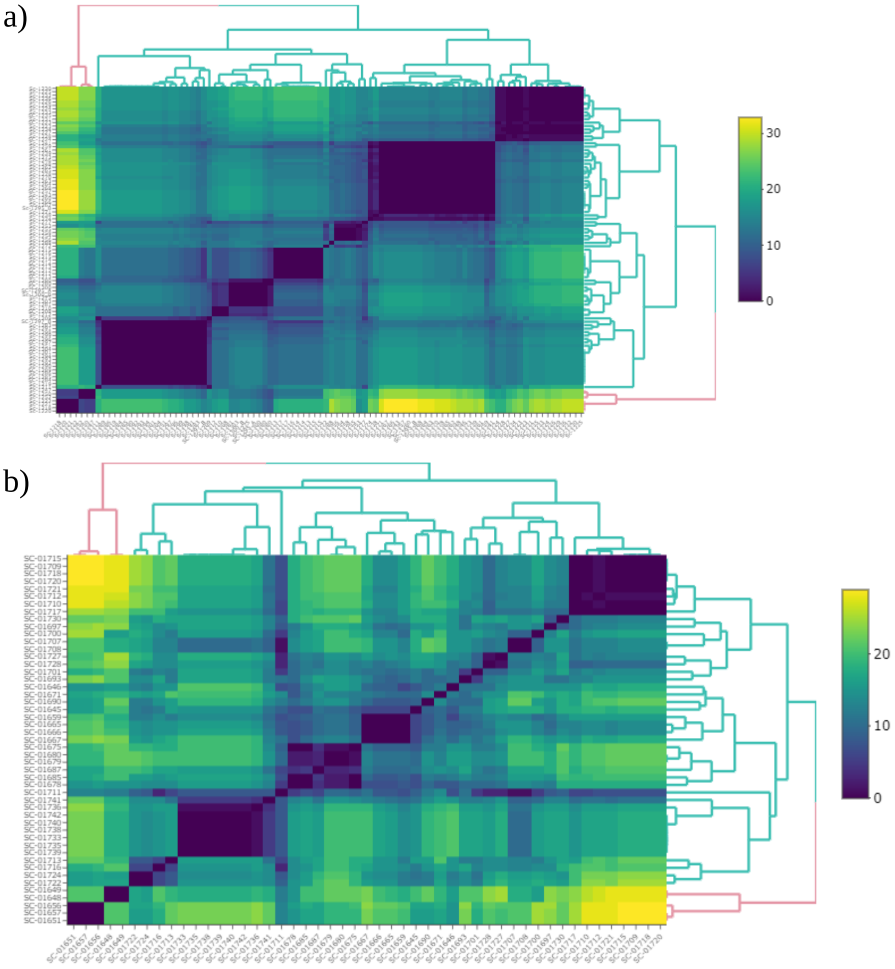

Fig. S12. Heat maps showing the number of allelic differences amongst scat samples collected in (a) 2021 and (b) 2022. Samples with similar genotypes are clustered on the diagonal. Sample IDs are shown on the X and Y axes.

2.6 Management Recommendations

For management of the metapopulation, the fastSTRUCTURE results show the optimum of k = 4 clusters across the source Ninu populations whereby groups can be broadly separated into 1) Yookamurra group, 2) Scotia group, 3) ZAA/Arid Recovery/Pilbara group and 4) Thistle Island group (Fig. S2). It is anticipated as the newly established and existing populations continue to breed, further population differentiation within the metapopulation may occur. Based on the analysis of 363 Ninu across the metapopulation, the following recommendations have been provided to and implemented by the Bilby National Recovery Team Metapopulation Management Committee, since 2018, following the 'research into management' approach outlined in Hogg *et al.*[94].

- Maximise genetic diversity across the metapopulation by sourcing individuals from the least similar population (as inferred from the F_{ST} and MK values) to the receiving population (Table 1; Table S10). That is, the more differentiated the populations the better in terms of long-term evolutionary potential and minimising effects of inbreeding.
- Maximise the value of the metapopulation by ensuring ongoing translocations are not using the same composition of individuals from the same source sites. That is, selection of individuals in different proportions from source sites will ensure that each location is somewhat genetically different from others in the metapopulation (Year 1 harvest: site A = 30, site B = 30; Year 2 harvest: site A = 20, site B = 40 – these two harvest events will

result in a genetic differentiation at the receiving sites due to the different proportions removed from the source sites). This will maintain, and potentially create genetic differentiation across the metapopulation providing for long-term maintenance of genetic diversity. It will also provide for future options when establishing new sites or augmenting existing sites. Due to the nature of the metapopulation, the natural processes of genetic drift will further facilitate genetic differentiation over time. See Table S10 for examples of this mixing the same source populations in different proportions in the releases undertaken after 2017.

- Populations that are genetically different but highly inbred (i.e. the mean inbreeding coefficient is higher than the founding groups/wild populations) should be mixed to reduce inbreeding effects (Table 2). Inbred populations that are genetically similar (show low F_{ST} values and high between population mean kinship) should not be mixed as this will potentially exacerbate inbreeding effects.
- Sourcing bilbies from wild sites to improve the current stock should be a priority. If possible, individuals from QLD should be sourced (either from QLD captive or wild) before individuals from WA/NT as the historic QLD management unit is under-represented in the current metapopulation.
- Collection and storage of genetic samples (ear biopsy in >80% ethanol stored in -20°C freezer) during translocation events and subsequent monitoring is essential to understanding the value of the translocations long-term, and our ability to monitor temporal changes.
- Another genetic survey from samples collected in 2024/25 (5-7 bilby generations) should be undertaken to assess the current translocations that are occurring. This will inform future translocation events and genetic management.
- A nationwide bilby survey that engages with Indigenous communities and uses scat sampling should be undertaken in 2024/25 to undertake a genetic assessment of wild populations and ascertain if the managed metapopulation is representative of the wild.

3 Ninu Genome Annotation and Gene Family Investigation

3.1 RNA extractions and transcriptome sequencing

Total RNA was extracted from 25mg of each tissue (spleen, liver, lymph node, kidney, heart, tongue, ovary, uterus, pouch skin, mammary gland, salivary gland, and testes) using the RNeasy Mini Kit (Qiagen, Catalogue 74004) and from blood using the RNEasy Protect animal blood kit (Qiagen, Catalogue 73224). Contaminating genomic DNA was removed through an on-column digestion using the RNase-free DNase I set (Qiagen, Catalogue 79254). RNA was quantified using the Bioanalyzer RNA 6000 Nano Kit (Agilent Technologies, Catalogue 5067-1511) prior to TruSeq stranded total RNA library preparation, with ribosomal RNA depletion using the Illumina Ribo-zero gold kit at the Ramaciotti Centre for Genomics (RCG; UNSW). Tissue libraries (excluding blood) were sequenced as 150 bp PE reads across a single S1 flowcell on the Illumina NovaSeq 6000. The blood library was sequenced as 75 bp PE reads across four lanes of a HO flowcell on the Illumina NextSeq 500. The testis library was sequenced as 100 bp PE reads across a single S1 flowcell on the Illumina NovaSeq 6000.

3.2 Transcriptome assembly

Raw RNA-seq reads (~100M reads per sample) underwent quality and length trimming using Trimmomatic v.0.38[73] in paired-end mode, with the parameters ILLUMINACLIP:2:30:10, SLIDINGWINDOW:4:5, LEADING:5, TRAILING:5 and MINLEN:25. FastQC v.0.11.8[95] was used to assess sequence quality of both raw and trimmed reads. To generate the global transcriptome of 12 tissues (spleen, liver, lymph node, kidney, heart, tongue, ovary, uterus,

pouch skin, mammary gland, salivary gland and blood), trimmed reads from were then aligned to the genome v.1.5 using HISAT2 v.2.1.0[96] with default parameters and alignments were converted and sorted using samtools. Transcripts were assembled using StringTie v.2.1.3 [97] and the resulting transcript models across the tissues were merged into a single global transcriptome using TAMA merge v.0.0 [98] with a splice junction threshold of 3, a 3-prime threshold of 500 and the option to merge duplicate transcript groups. The resultant transcripts were then filtered by removing transcripts with weak evidence (only found in one tissue and FPKM < 0.1) or single-exon transcripts with either low read support or low coding potential. Coding potentials of transcripts were determined using CPC2 v.2.0 [99]. Transcriptome completeness was assessed using BUSCO v.5.4.6 (mammalia_odb10; n=9226) as above. TransDecoder v.2.0.1 [100] was used to determine coding regions and open reading frames within transcripts. Following genome annotation, transcripts were assembled using StringTie v.2.1.3 with the GeMoMa genome annotation as a guide (see section 2.3) to generate FPKM counts for each transcript within the global transcriptome. The testis transcriptome was generated using the same methods as above.

In total 528 GB of raw sequencing data was produced across the 13 tissues, comprising 78 to 132 million read pairs per tissue. Over 99.36% of read pairs were retained post-trimming amongst the 13 tissues. The global transcriptome of 12 tissues was generated as above and aligned to v.1.5. The transcriptome (including non-coding transcripts) was composed of 39,106 genes and 303,420 isoforms with an average transcript length of 6,833 bp and an N50 of 13.4 kb (Extended Data Table 3). For all protein-coding transcripts, the longest open reading frame had an average transcript length of 1,010bp and N50 of 1,620bp. The global transcriptome contained 86.1% complete BUSCOs, with 3.0% fragmented and 10.9% missing mammalia_odb10 genes (Extended Data Table 3). TransDecoder predicted 285,984 coding regions, of which 70.06% were complete (contained a start and stop codon) and 27,001 had unique hits to the Swiss-Prot non-redundant database. The testis transcriptome was generated as above and aligned to the reference genome v.1.5. The transcriptome was comprised on 37,034 genes and 82,964 isoforms with an average transcript length of 2,363 bp and an N50 of 3.93 kb (Extended Data Table 3). The testis transcriptome contained 75.5% complete BUSCOs, with 4.0% fragmented and 20.5% missing mammalia odb10 genes (Extended Data Table 3). TransDecoder predicted 75,224 coding regions, of which 64.58% were complete and 19,203 had unique hits to Swiss-Prot non-redundant database.

3.3 Genome annotation

Transcripts from the global transcriptome which represented isoforms which contained the longest complete ORF (16,316) were used as mRNA-based evidence for genome annotation. 49.44% of the genome was masked as repetitive and a total of 28,488 protein-coding genes were annotated in the genome of which 15,916 were based on mRNA evidence, 3,096 were based on protein homology evidence and the remaining were made *ab initio*.

A homology-based annotation was created using GeMoMa v.1.8[101] using the annotation from nine mammalian genomes (cow, human, opossum, mouse, Tammar wallaby, platypus, koala, Tasmanian devil, wombat). GeMoMa annotated 63,480 isoforms for 38,756 genes, with a median Ninu:opossum protein length ratio of 0.986 versus the *Monodelphis domestica* reference proteome. This was similar to the 39,106 genes in the global transcriptome and was rated as 96.0% complete by BUSCO v.5 (proteome mode). The average GeMoMa gene prediction was 1,120 bp (lacking UTRs) with an average of 6.32 exons per gene.

For the final repeat annotation, RepeatModeler v.2.0.1[102] was used to create a custom repeat database using the HiC-scaffolded genome with the repeat contigs added back (see above). RepeatMasker v.4.1.0 was used to identify and mask repeats from this library (excluding low

complexity regions and simple repeats) on the final version of the assembly[103] (see Supplementary Excel). In total, 47.87% of the assembly was annotated as interspersed repeats, with L1 LINEs being the dominant repeat type (20.91% assembly), and a further 6.22% as low complexity and simple repeats (Table S14). Additional annotation of RNA genes was performed with barrnap v.0.9[104] (rRNA) and Infernal v.1.1.3[105] (tRNA). Telomeres were predicted using Diploidocus v.1.3.1[12] with additional telomere repeat sequences predicted using tidk v.0.1.5 [https://github.com/tolkit/telomeric-identifier]. Nuclear mitochondrial DNA fragments (NUMTs) identified using NUMTFinder v.0.5.3[25] using the assembled Ninu mtDNA genome (above).

Table S14: Ninu reference genome repeat statistics

Repeat Classes			
Total Sequences	609		
Total Length (bp)	3,655,732,724		
Class	Count	bp Masked	% masked
DNA	298	31,092	0.00
Ginger-1	518	182,063	0.00
TcMar-Mariner	2,024	313,297	0.01
TcMar-Tc1	10,759	936,058	0.03
TcMar-Tigger	22,012	5,637,191	0.15
hAT	1,931	379,419	0.01
hAT-Charlie	110,993	16,572,073	0.45
hAT-Tip100	43,540	7,675,332	0.21
LINE			
CR1	373,723	66,547,716	1.82
Dong-R4	9,918	31,56,447	0.09
L1	1,987,892	764,315,940	20.91
L2	836,612	173,028,625	4.73
RTE	11,138	1,986,365	0.05
RTE-BovB	87,021	42,912,194	1.17
LTR (no subclass)	315	48,905	0.00
ERV1	8,427	4,724,499	0.13
ERVK	30,098	15,296,680	0.42
ERVL	4,293	2,198,823	0.06
Gypsy	2,566	1,062,056	0.03
SINE			
Alu	10,384	2,105,586	0.06
MIR	2,657,873	401,388,147	10.98
tRNA-RTE	597	69,807	0.00
Unknown	1,222,700	239,347,740	6.55
Total Interspersed	*7,435,632*	*1,749,916,055*	*47.87*
Low complexity	129,569	968,757	0.27
Simple repeat	1,155,394	211,868,487	5.80
rRNA	8,718	2,131,914	0.06
snRNA	1,297	100,347	0.00
Total	8730,610	1,973,706,560	53.99

3.4 Chromosome synteny plots

To further validate the chromosome-level assembly and investigate chromosomal rearrangements in the Ninu, high-level synteny was plotted against six other marsupial species (Table S15). GENESPACE v.1.3.1[106] was used to plot genomic synteny.

Table S15. Marsupial reference genomes used for chromosome synteny plots.

Species	Genbank ID	Assembly ID	Reference
Mardo (*Antechinus flavipes*)	GCA_016432865.1	AdamAnt	[107]
Monito del Monito (*Dromiciops gliroides*)	GCF_019393635.1	mDroGli1.pri	VGP*
Short-tailed Opossum (*Monodelphis domestica*)	GCF_000002295.2	MonDom5	[108]
Devil (*Sarcophilus harrisii*)	GCF_902635505.1	mSarHar1.11	[109]
Brush-tailed possum (*Trichosurus vulpecula*)	GCF_011100635.1	mTriVul1.pri	[110]
Agile gracile mouse opossum (*Gracilinanus agilis)*	GCA_016433145.1	AgileGrace	[111]
Koala (*Phascolarctos cinereus*)	n/a	04Oct2019_BU56K	*Unpublished data*

* Provided by the Vertebrate Genome Project

3.5 X-chromosome read depth and meiotic immunofluorescence

X chromosome read depth

DeepTools bamCoverage was used to calculate read depth of male Illumina data in 20kb windows. A heatscatter of the read depth ratios (versus the PAR mean) was plotted with the R package LSD[112].

Meiotic spreads and immunofluorescence (IF)

Using the testis from the Ninu male, spermatocyte spreads were obtained as previously described[113]. Briefly, a piece of the testicular biopsy was carefully minced on a slide and treated with 1% Lipsol for 30 min at room temperature. Then, a fixative solution containing 4% paraformaldehyde was added, and slides were kept in a humid chamber for two hours. Slides were washed in 1% photo-flo solution and further processed for immunofluorescence, or frozen at −20°C until use. Immuno-staining of meiocytes was performed using rabbit antibody against SYCP3 (Abcam, Catalogue #ab15093, 1:100) and rabbit antibody against SYCP1 (Abcam, Catalogue #ab15087) incubated overnight at 4°C in a humid chamber. Fluorochrome-conjugated secondary antibodies were diluted in PBST (Tween 0.05% in PBS) and incubated for 1 h at 37°C in a humid chamber. DNA was counterstained with anti-fade solution containing 8 μg/ml DAPI (4′,6′-diamidino-2-phenylindole).

3.6 Gene family investigation

To investigate the evolution of gene family size in Ninu, we compared the proteome with those of other marsupials (opossum, Tasmanian devil, koala, brown antechinus, and Tammar wallaby), eutherians (human, mouse, and cow), and a monotreme (platypus) (Table S16). These taxa were selected to elucidate whether changes in gene family sizes occurred specifically in the Ninu lineage or is shared across other marsupials. Protein sequences were re-annotated using GeMoMa v.1.8[101] using the same nine mammalian reference genomes as for the Ninu (above, Table S16) to minimise the impact of incomplete annotations on analyses of gene family sizes, in particular, to avoid inflated estimates of gene family contraction. To control

for pseudogenes, we removed genes annotated as "Predicted protein" or "Reverse transcriptase homologs" from further analyses. Orthologous genes were identified with OrthoFinder v.2.4.01 using default settings with the 10 annotated proteomes as inputs. Orthogroups were putatively assigned a gene according to the most commonly occurring annotation within the orthogroup.

Table S16. Assemblies used for GeMoMa annotation and gene expansion analyses

Common name	Scientific name	Assembly ID	Database	Reference
Brown antechinus	*Antechinus stuartii*	antechinusM_pseudohap2.1.proteins.fa#	GigaScience Database[20]	[114]
Tasmanian devil	*Sarcophilus harrisii*	mSarHar1.11*#	Ensembl[24]	[109]
Koala	*Phascolarctos cinereus*	phaCin_unsw_v4.1*#	Ensembl[23]	[115]
Wombat	*Vombatus ursinus*	bare-nosed_wombat_genome_assembly*	Ensembl	
Tammar wallaby	*Notamacropus eugenii*	Meug_1.0*#	Ensembl	[116]
Opossum	*Monodelphis domestica*	ASM229v1*#	Ensembl[21]	[108]
Platypus	*Ornithorhynchus anatinus*	mOrnAna1.p.v.a*<br>mOrnAna1.pri.v4#	RefSeq[22]	[117]
Human	*Homo sapiens*	GRCh38.p13*#	Ensembl	
Mouse	*Mus musculus*	GRCm39*#	Ensembl	
Cow	*Bos taurus*	ARS-UCD1.2*#	Ensembl	

* Used for GeMoMa annotation; #Used in gene expansion analysis.

A dated species tree was constructed using MCMCTree in PAML v.4.9 [118] following Jeffares *et al*.[119]. We retrieved the species tree topology and prior distributions of the internal node ages, including the root[120,121] (Table S17). We aligned all single-copy orthogroups identified by OrthoFinder (*n* = 3,181) using MAFFT v.7.407[122] with default settings and we filtered out poorly aligned regions using TrimAl v.1.4.15 [123] with `--automated1`. Aligned and trimmed sequences were concatenated with AMAS v.1.0 [124] and used as input to MCMCTree. First, MCMCTree estimated branch lengths with baseml, followed by divergence time estimation with approximate likelihood calculation. We used the F81 substitution model (`model=2`) and accounted for among-lineage rate variation using an independent-rates relaxed clock (`clock=2`). The posterior distribution was estimated by drawing 20,000 MCMC samples, with a thinning interval of 10 steps, after 2000 discarded burn-in samples.

Table S17. Secondary node calibrations used for the species tree.

Node	Uniform calibration prior* (Myr)	Reference
Mammalia (root)	164.9–250.8	121
Theria (Placentalia, Marsupialia)	123.3–166.2	121
Placentalia (Euarchontoglires, cow)	73.2–77.4	121
Euarchontoglires (mouse, human)	69.6–73.8	121
Marsupialia (Eomarsupialia, opossum)	44.7–70.4	121
Eomarsupialia (Diprotodontia, Agreodontia)	58.8–67.8	120
Diprotodontia (koala, tammar wallaby)	49.9–58.3	120
Agreodontia (Ninu, Tasmanian devil, antechinus)	57.3–66.3	120
Antechinus stuartii - *Sarcophilus harrisii* (Tasmanian devil, antechinus)	18.1–22.7	120

We tested for expansions of gene families under a birth-death model using CAFE v.5.0[125]. The gene counts from OrthoFinder and dated species tree from MCMCTree were used as inputs for CAFE. To minimise the impact of gene families (orthogroups) with highly variable gene counts when estimating lambda, gene families with 100 or more genes in any one lineage were analysed separately. For both subsets of gene families, we estimated multiple lambdas for monotremes, marsupials, and eutherians (-y) using a Poisson root frequency distribution (-p) and a model to account for genome assembly and annotation errors (-e). First, we analysed gene families with fewer than 100 genes to estimate the optimal lambdas for each of the three lineages. The multiple estimated lambdas were then fixed per lineage (-m) for gene families with 100 or more genes in any lineage. Gene ontology (GO) term annotation was conducted on orthogroups deemed to be significantly expanded in Ninu. We used GOnet[52] with the biological process namespace and generic GO slim subset for humans.

Across all 10 species, 74,591 genes were annotated as a "Predicted protein" and 123,379 genes were annotated as a "Reverse transcriptase homolog". These were omitted from further analyses, leaving a total of 197,970 annotated genes (Table S18). In total, 185,847 annotated genes (93.9%; Table S18) were assigned into 17,082 orthogroups; 6350 orthogroups (37.2%) had all species present and 3181 were single-copy orthogroups (18.6%).

Table S18. Number of genes annotated and assigned to orthogroups. Genes annotated by GeMoMa as either "Predicted protein" or "Reverse transcriptase homologs" were omitted and orthology assignment was conducted on the remaining genes.

	Annotated genes (GeMoMa)				Orthology assignment (OrthoFinder)	
Species	Total genes	Predicted proteins	Reverse transcriptase homologs	Retained	Genes assigned to an orthogroup	Genes in species-specific orthogroups
Brown antechinus	32,500	7044	11,624	18,668	18,086	199
Ninu	38,417	9948	13,000	22,948	21,129	464
Cow	36,299	7630	11,831	19,461	18,165	819
Human	35,649	9371	11,606	20,977	19,564	797
Opossum	39,011	4737	14,077	18,814	17,552	777
Mouse	38,602	9204	12,992	22,196	20,520	2082
Tammar wallaby	31,166	8587	10,582	19,169	17,502	207
Platypus	28,540	4600	11,946	16,546	16,088	1038
Koala	37,264	7571	13,753	21,324	19,863	240
Tasmanian devil	32,514	5899	11,968	17,867	17,378	67
Total	349,962	74,591	123,379	197,970	185,847	6690

The CAFE analysis successfully analysed 12,653 (74.07%) orthogroups (i.e., the gene family was present at the root of the tree in the most recent common ancestor of all mammals). Of these orthogroups, 1,206 consisted of one or more lineages that were considered significantly fast evolving by CAFE (Extended Data Fig. 3). We found 459 orthogroups that were significantly expanded in Ninu and successfully annotated 369/435 unique genes using GOnet. Missed GO term annotations were either due to genes lacking an annotation in humans (e.g., *CYP2A11* and *CYP2B4* from *Oryctolagus cuniculus*) or absent from UniProt altogether. Gene families putatively expanded in the Ninu were primarily involved in anatomical structure development (GO:0048856, GO:0009790, GO:0048646, GO:0000902), response to stress (GO:0006950, GO:0007165), and a range of metabolic processes (Table S5).

3.7 Olfactory receptor genes

Although adjustment of clustering parameters may impact orthology assignment and the outcome of gene family expansion tests, the Ninu remains with the highest number of annotated ORs[126,127]. In comparison to previously reported OR gene copy numbers of the cow, human, mouse, and platypus; the total number of annotated OR genes here were far smaller. However, the totals here correspond approximately to the number of intact OR genes. Between 17.3% and 59.4% of mammalian OR genes have become pseudogenes. These pseudogenes may have been discarded when "Predicted proteins" and "Reverse transcriptase homologs" were omitted prior to gene family expansion analyses. Assuming that only intact OR genes were annotated,

Ninu have more OR genes than some eutherian mammals such as cows and dogs, but considerably fewer than elephants. Targeted analysis of OR genes is needed to characterise the repertoire of ORs in the Ninu, particularly the classification of both functional genes and pseudogenes.

OR1D2 and *OR1D5* orthogroups are presented in Supplementary Excel. Each orthogroup consists of the estimated likelihood that the orthogroup is 'real', the change of copy number in the Ninu lineage, and whether the change was deemed significant by CAFE. *OR1D2* genes were assigned into 362 orthogroups, with 17 orthogroups deemed significantly expanding by CAFE (Supplementary Excel). *OR1D5* genes were assigned into 165 orthogroups, with 10 significantly expanding (Supplementary Excel). Although adjustment of clustering parameters may affect orthology assignment and the outcome of gene family expansion tests, the Ninu remains with the highest number of annotated ORs. Although the total number of annotated OR genes in our dataset is smaller than those previously reported for cow, human, mouse, and platypus; our totals correspond approximately to the number of intact OR genes[126] and may be the consequence of discarding pseudogenes. Assuming that only intact OR genes were annotated, Ninu have more OR genes than some eutherian mammals, such as cow and mouse, but considerably fewer than elephants (1,948 intact OR genes[128]). Further targeted analysis of OR genes is needed to definitively characterise the repertoire of ORs in the Ninu, particularly the classification of both functional genes and pseudogenes.

3.8 Characterisation of Ninu immune genes

Immune genes were annotated using multiple search strategies. BLAST v.2.2.30[129] was used to search the Ninu reference assembly, associated annotation files and/or transcriptomes using published marsupial, monotreme and eutherian immune gene sequences as queries, with default parameters and an e-value threshold of 10 so as not to exclude any potential gene candidates. HMMER v.3.2[130] was also used to identify putative genes within immune families that are known to contain duplications in other marsupials[131,132], such as NK receptors. Hidden Markov models (HMM) were constructed using ClustalW alignments of published marsupial and eutherian immune gene sequences, which were then used to search all genomes and transcriptomes using HMMER v.3.2 with an e-value threshold of 10. For variable segments of T cell receptor and Immunoglobulin families, recombination signal sequences (RSS) downloaded from the IMGT database [133] and published koala sequences [134], were aligned using ClustalW and used to construct HMM. These RSS HMM were then used to search the Ninu genome (v1.5) using HMMER, to identify conserved RSS which flank each variable segment. For NK receptors, putative NKC and LRC sequences from BLAST and HMMER searches were queried against the Swiss-Prot nonredundant database, and any sequences with top hits to Swiss-Prot NK genes, marsupial-specific NK genes or the PFAM immunoglobulin domain PF00047 or C-type lectin domain PF00059 HMM model were retained. Immunoglobulin superfamily (IGSF) domains within putative NK sequences from each species were identified using the SMART database, and IGSF domains within 5kb were considered exons of a single LRC gene. Putative immune genes were named following the appropriate nomenclature for each family, with duplicated genes named according to their genomic location from the 5' to 3' end of the locus.

MHC Class I and II genes were named based on their evolutionary relationship with other marsupial MHC genes. Phylogenetic trees were constructed using the neighbour-joining method [135] with 1000 bootstraps [136] in MEGA11[137]. Genes with clear homologous relationships to marsupial MHC genes were assigned names based on their marsupial counterparts. Genes with no clear relationship were assigned species-specific names. Ninu MHC Class II genes were classified into three classical clusters; DA, DB and DC and one nonclassical cluster;

DM[138,139] based on their homology to marsupial genes (Fig. S3). For MHC Class I, rapid evolution means that clear orthologous relationships across marsupial genes are rarely observed. Classical class I genes are generally highly polymorphic in comparison to nonclassical class I[140]. Genetic variability within the α1, α2 and α3 domains (exons 2, 3 and 4 respectively) were assessed for classification.

Class II genes were classified into classical and non-classical based on homology to other marsupial class II genes. Two genes were classified as non-classical (*DMA* and *DMB*) class II genes. For the MHC-I genes, clear homology and strong bootstrap support was shown between the UM gene of the Tasmanian devil (*Saha*), opossum and Ninu (*Mala*) (Fig. S3). Two class I genes showed characteristics of classical MHC genes based on genetic variability. Mala-*UA* and -*UB* had between five and 38 variants in each of the α1, α2 and α3 domains, in comparison to the predicted non-classical genes that had between zero and six variants within a domain (Table S19).

Table S19: Genetic variability of the functional α1, α2 and α3 domains of the identified MHC-I genes

Gene	# of SNPs at each domain		
	α1	α2	α3
Mala-*UA*	5	15	15
Mala-*UB*	6	38	18
Mala-*UC*	6	1	0
Mala-*UD*	3	0	2
Mala-*UE*	2	1	2
Mala-*UM*	1	1	2

4 Supplementary References

1. Weisenfeld, N. I., Kumar, V., Shah, P., Church, D. M., & Jaffe, D. B. Direct determination of diploid genome sequences. *Genome Res.* **27**: 757-67 (2017).

2. Wenger, A. M., et al. Accurate circular consensus long-read sequencing improves variant detection and assembly of a human genome. *Nat. Biotechnol.* **37**: 1155-62 (2019).

3. Guan, D., et al. Identifying and removing haplotypic duplication in primary genome assemblies. *Bioinform.* **36**: 2896-98 (2020).

4. Zheng, G. X., et al. Haplotyping germline and cancer genomes with high-throughput linked-read sequencing. *Nat. Biotechnol.* **34**: 303-11 (2016).

5. Li, H. & Durbin, R. Fast and accurate short read alignment with Burrows–Wheeler transform. *Bioinform.* **25**: 1754-60 (2009).

6. Li, H., et al. The sequence alignment/map format and SAMtools. *Bioinform.* **25**: 2078-79 (2009).

7. Yeo, S., Coombe, L., Warren, R. L., Chu, J., & Birol, I. ARCS: Scaffolding genome drafts with linked reads. *Bioinform.* **34**: 725-31 (2018).

8. Warren, R. L., et al. LINKS: Scalable, alignment-free scaffolding of draft genomes with long reads. *GigaScience* **4**: s13742-015-0076-3 (2015).

9. English, A. C., et al. Mind the gap: Upgrading genomes with Pacific Biosciences RS long-read sequencing technology. *PLoS One* **7**: e47768 (2012).

10. Walker, B. J., et al. Pilon: An integrated tool for comprehensive microbial variant detection and genome assembly improvement. *PLoS One* **9**: e112963 (2014).

11. Bushnell, B. *BBTools*. 2014 23 August 2020]; Available from: https://sourceforge.net/projects/bbmap/.

12. Chen, S. H., et al. Chromosome-level de novo genome assembly of *Telopea speciosissima* (New South Wales waratah) using long-reads, linked-reads and Hi-C. *Mol. Ecol. Resour.* **22**: 1836-54 (2022).

13. Edwards, R. J. *GapSpanner: Genome assembly gap long read support and reassembly tool*. 2021.

14. Kolmogorov, M., Yuan, J., Lin, Y., & Pevzner, P. A. Assembly of long, error-prone reads using repeat graphs. *Nat. Biotechnol.* **37**: 540-46 (2019).

15. Kundu, R., Casey, J., & Sung, W.-K. HyPo: Super fast & amp; accurate polisher for long read genome assemblies. *bioRxiv*: 2019.12.19.882506 (2019).

16. Kokot, M., Długosz, M., & Deorowicz, S. KMC 3: Counting and manipulating k-mer statistics. *Bioinform.* **33**: 2759-61 (2017).

17. Li, H. Minimap2: Pairwise alignment for nucleotide sequences. *Bioinform.* **34**: 3094-100 (2018).

18. Putnam, N. H., et al. Chromosome-scale shotgun assembly using an in vitro method for long-range linkage. *Genome Res.* **26**: 342-50 (2016).

19. Li, H. Aligning sequence reads, clone sequences and assembly contigs with BWA-MEM. *arXiv* (2013).

20. Goloborodko, A., Abdennur, N., Venev, S., hbbrandao, & gfudenberg, mirnylab/pairtools: v0. 2.2. 2019, Zenodo.

21. Abdennur, N. & Mirny, L. A. Cooler: scalable storage for Hi-C data and other genomically labeled arrays. *Bioinform.* **36**: 311-16 (2020).

22. Ramírez, F., et al. High-resolution TADs reveal DNA sequences underlying genome organization in flies. *Nat. Comms.* **9**: 1-15 (2018).

23. Kerpedjiev, P., et al. HiGlass: Web-based visual exploration and analysis of genome interaction maps. *Genome Biol.* **19**: 1-12 (2018).

24. Xu, G.-C., et al. LR_Gapcloser: A tiling path-based gap closer that uses long reads to complete genome assembly. *GigaScience* **8** (2018).

25. Edwards, R. J., et al. Chromosome-length genome assembly and structural variations of the primal Basenji dog (*Canis lupus familiaris*) genome. *BMC Genomics* **22**: 188 (2021).
26. Davey, N. E., Shields, D. C., & Edwards, R. J. SLiMDisc: SWhort, linear motif discovery, correcting for common evolutionary descent. *Nucleic Acids Res.* **34**: 3546-54 (2006).
27. Cheng, H., et al. Haplotype-resolved assembly of diploid genomes without parental data. *Nat. Biotechnol.* **40**: 1332-35 (2022).
28. Uliano-Silva, M., et al. MitoHiFi: a python pipeline for mitochondrial genome assembly from PacBio high fidelity reads. *BMC Bioinform.* **24**: 288 (2023).
29. Allio, R., et al. MitoFinder: Efficient automated large-scale extraction of mitogenomic data in target enrichment phylogenomics. *Mol. Ecol. Resour.* **20**: 892-905 (2020).
30. Meng, G., Li, Y., Yang, C., & Liu, S. MitoZ: a toolkit for animal mitochondrial genome assembly, annotation and visualization. *Nucleic Acids Res.* **47**: e63-e63 (2019).
31. Simao, F. A., Waterhouse, R. M., Ioannidis, P., Kriventseva, E. V., & Zdobnov, E. M. BUSCO: Assessing genome assembly and annotation completeness with single-copy orthologs. *Bioinform.* **31**: 3210-12 (2015).
32. Stuart, K. C., et al. Transcript- and annotation-guided genome assembly of the European starling. *Mol. Ecol. Resour.* **22**: 3141-60 (2022).
33. Rhie, A., Walenz, B. P., Koren, S., & Phillippy, A. M. Merqury: Reference-free quality, completeness, and phasing assessment for genome assemblies. *Genome Biol.* **21**: 245 (2020).
34. Patil, I. Visualizations with statistical details: The'ggstatsplot'approach. *J. Open Source Softw.* **6**: 3167 (2021).
35. Fulton, T. L., Wagner, S. M., & Shapiro, B. Case study: Recovery of ancient nuclear DNA from toe pads of the extinct passenger pigeon. *Methods Mol. Biol.* **840**: 29-35 (2012).
36. Meyer, M. & Kircher, M. Illumina sequencing library preparation for highly multiplexed target capture and sequencing. *Cold Spring Harb. Protoc.* **2010**: pdb.prot5448 (2010).
37. Miller, N. A., et al. A 26-hour system of highly sensitive whole genome sequencing for emergency management of genetic diseases. *Genome Med.* **7**: 1-16 (2015).
38. Danecek, P., et al. Twelve years of SAMtools and BCFtools. *GigaScience* **10**: giab008 (2021).
39. Li, H. A statistical framework for SNP calling, mutation discovery, association mapping and population genetical parameter estimation from sequencing data. *Bioinform.* **27**: 2987-93 (2011).
40. Wang, K., Li, M., & Hakonarson, H. ANNOVAR: Functional annotation of genetic variants from high-throughput sequencing data. *Nucleic Acids Res.* **38**: e164-e64 (2010).
41. Purcell, S., et al. PLINK: a tool set for whole-genome association and population-based linkage analyses. *Am. J. Med. Genet.* **81**: 559-75 (2007).
42. Van der Auwera, G. A. & O'Connor, B. D. *Genomics in the cloud: using Docker, GATK, and WDL in Terra (1st Edition).* O'Reilly Media.p. (2020).
43. Stamatakis, A. RAxML version 8: a tool for phylogenetic analysis and post-analysis of large phylogenies. *Bioinform.* **30**: 1312-13 (2014).
44. Ronquist, F., et al. MrBayes 3.2: Efficient Bayesian Phylogenetic Inference and Model Choice Across a Large Model Space. *Syst. Biol.* **61**: 539-42 (2012).
45. Feigin, C. Y., et al. Genome of the Tasmanian tiger provides insights into the evolution and demography of an extinct marsupial carnivore. *Nat. Ecol. Evol.* **2**: 182-92 (2018).
46. Bushnell, B. *BBTools*. 2014; Available from: sourceforge.net/projects/bbmap/.
47. Broad Institute, G. R. *Picard Toolkit*. 2019.
48. McKenna, A., et al. The Genome Analysis Toolkit: a MapReduce framework for analyzing next-generation DNA sequencing data. *Genome Res.* **20**: 1297-303 (2010).
49. Danecek, P., et al. The variant call format and VCFtools. *Bioinform.* **27**: 2156-58 (2011).

50. Batley, K. C., et al. Whole genomes reveal multiple candidate genes and pathways involved in the immune response of dolphins to a highly infectious virus. *Mol. Ecol.* **30**: 6434-48 (2021).

51. Quinlan, A. R. & Hall, I. M. BEDTools: a flexible suite of utilities for comparing genomic features. *Bioinform.* **26**: 841-42 (2010).

52. Pomaznoy, M., Ha, B., & Peters, B. GOnet: a tool for interactive Gene Ontology analysis. *BMC Bioinform.* **19**: 1-8 (2018).

53. Supek, F., Bošnjak, M., Škunca, N., & Šmuc, T. REVIGO summarizes and visualizes long lists of gene ontology terms. *PLoS One* **6**: e21800 (2011).

54. Pedersen, B. S. & Quinlan, A. R. Mosdepth: Quick coverage calculation for genomes and exomes. *Bioinform.* **34**: 867-68 (2018).

55. Schiffels, S. & Wang, K. MSMC and MSMC2: The Multiple Sequentially Markovian Coalescent in *Statistical Population Genomics*, Dutheil, J. Y., Editor., Springer US: New York, NY. p. 147-66 (2020).

56. Lott, M. J., Wright, B. R., Kemp, L. F., Johnson, R. N., & Hogg, C. J. Genetic management of captive and reintroduced bilby populations. *J. Wildl. Manag.* **84**: 20-32 (2020).

57. Miller, E. J., Eldridge, M. D. B., Morris, K., Thomas, N., & Herbert, C. A. Captive management and the maintenance of genetic diversity in a vulnerable marsupial, the greater bilby. *Aust. Mammal.* **37**: 170-81 (2015).

58. Southgate, R. & Possingham, H. P. Modeling the reintroduction of the greater bilby *Macrotis lagotis* using the metapopulation model analysis of the likelihood of extinction (ALEX). *Biol. Conserv.* **73**: 151-60 (1995).

59. Woinarski, J., Burbidge, A., & Harrison, P. A review of the conservation status of Australian mammals. *Therya* **6**: 155-66 (2015).

60. Lynch, M., et al. Genetic drift, selection and the evolution of the mutation rate. *Nat. Rev. Genet.* **17**: 704-14 (2016).

61. Uchimura, A., et al. Germline mutation rates and the long-term phenotypic effects of mutation accumulation in wild-type laboratory mice and mutator mice. *Genome Res.* **25**: 1125-34 (2015).

62. Duntsch, L., Whibley, A., Brekke, P., Ewen, J. G., & Santure, A. W. Genomic data of different resolutions reveal consistent inbreeding estimates but contrasting homozygosity landscapes for the threatened Aotearoa New Zealand hihi. *Mol. Ecol.* (2021).

63. Curik, I., Ferenčaković, M., & Sölkner, J. Inbreeding and runs of homozygosity: a possible solution to an old problem. *Livest. Sci.* **166**: 26-34 (2014).

64. Meyermans, R., Gorssen, W., Buys, N., & Janssens, S. How to study runs of homozygosity using PLINK? A guide for analyzing medium density SNP data in livestock and pet species. *BMC Genomics* **21**: 1-14 (2020).

65. Christie, P. M., The Australasian Species Management Plan for Zoo Populations of the Greater Bilby, *Macrotis lagotis*. 1991, Species Management Co-ordinating Council (SMCC), ARAZPA: Sydney, Australia. p. 40.

66. ZAA, Greater bilby annual report and recommendations. 2016, Zoo and Aquarium Association Australasia: Mosman, NSW.

67. Bradley, K., Lees, C., & Lundie-Jenkins, G. *Greater bilby recovery summit 2015: Report and interim conservation plan*, Apple Valley, USA: IUCN Species Survival Commission (SSC), Conservation Breeding Specialist Group (CBSG).p. 125 (2015).

68. Wright, B., et al. Impact of reduced-representation sequencing protocols on detecting population structure in a threatened marsupial. *Mol. Biol. Rep.* **46**: 5575-80 (2019).

69. Dziminski, M. A., Carpenter, F. M., & Morris, F. Range of the greater bilby (*Macrotis lagotis*) in the Pilbara Region, Western Australia. *J. Royal. Soc. WA* **103**: 97-102 (2020).

70. Wright, B., et al. From reference genomes to population genomics: comparing three reference-aligned reduced-representation sequencing pipelines in two wildlife species. *From reference genomes to population genomics: comparing three reference-aligned reduced-representation sequencing pipelines in two wildlife species* **20**: 1-10 (2019).
71. Catchen, J., Hohenlohe, P. A., Bassham, S., Amores, A., & Cresko, W. A. Stacks: an analysis tool set for population genomics. *Mol. Ecol.* **22**: 3124-40 (2013).
72. Catchen, J. M., Amores, A., Hohenlohe, P., Cresko, W., & Postlethwait, J. H. Stacks: Building and genotyping loci de novo from short-read sequences. *G3* **1**: 171-82 (2011).
73. Bolger, A. M., Lohse, M., & Usadel, B. Trimmomatic: a flexible trimmer for Illumina sequence data. *Bioinform.* **30**: 2114-20 (2014).
74. Langmead, B. & Salzberg, S. L. Fast gapped-read alignment with Bowtie2. *Nat. Methods* **9**: 357-59 (2012).
75. R Core Team, R: a language and environment for statistical computing. 2021, R Foundation for Statistical Computing: Vienna (Austria).
76. Goudet, J. HIERFSTAT, a package for R to compute and test hierarchical F-statistics. *Mol. Ecol. Notes* **5**: 184-86 (2005).
77. Gruber, B., Unmack, P. J., Berry, O. F., & Georges, A. dartr: An r package to facilitate analysis of SNP data generated from reduced representation genome sequencing. *Mol. Ecol. Resour.* **18**: 691-99 (2018).
78. Keenan, K., McGinnity, P., Cross, T. F., Crozier, W. W., & Prodöhl, P. A. diveRsity: An R package for the estimation and exploration of population genetics parameters and their associated errors. *MEE* **4**: 782-88 (2013).
79. Kardos, M., Taylor, H. R., Ellegren, H., Luikart, G., & Allendorf, F. W. Genomics advances the study of inbreeding depression in the wild. *Evol. Appl.* **9**: 1205-18 (2016).
80. Wang, J. Coancestry: A program for simulating, estimating and analysing relatedness and inbreeding coefficients. *Mol. Ecol. Resour.* **11**: 141-45 (2011).
81. Pembleton, L. W., Cogan, N. O., & Forster, J. W. StAMPP: an R package for calculation of genetic differentiation and structure of mixed-ploidy level populations. *Mol. Ecol. Resour.* **13**: 946-52 (2013).
82. Do, C., et al. NeEstimator v2: Re-implementation of software for the estimation of contemporary effective population size (Ne) from genetic data. *Mol. Ecol. Resour.* **14**: 209-14 (2014).
83. Peel, D., Waples, R. S., Macbeth, G. M., Do, C., & Ovenden, J. R. Accounting for missing data in the estimation of contemporary genetic effective population size (Ne). *Mol. Ecol. Resour.* **13**: 243-53 (2013).
84. Waples, R. K., Larson, W. A., & Waples, R. S. Estimating contemporary effective population size in non-model species using linkage disequilibrium across thousands of loci. *Heredity* **117**: 233-40 (2016).
85. Jones, A. T., Ovenden, J. R., & Wang, Y. G. Improved confidence intervals for the linkage disequilibrium method for estimating effective population size. *Heredity* **117**: 217-23 (2016).
86. Raj, A., Stephens, M., & Pritchard, J. K. fastSTRUCTURE: variational inference of population structure in large SNP data sets. *Genet.* **197**: 573-89 (2014).
87. Zheng, X., et al. A high-performance computing toolset for relatedness and principal component analysis of SNP data. *Bioinform.* **28**: 3326-28 (2012).
88. Peakall, R. & Smouse, P. E. GenAlEx 6.5: genetic analysis in Excel. Population genetic software for teaching and research—an update. *Bioinform.* **28**: 2537-39 (2012).
89. Waits, L. P., Luikart, G., & Taberlet, P. Estimating the probability of identity among genotypes in natural populations: Cautions and guidelines. *Mol. Ecol.* **10**: 249-56 (2001).

90. Sunnucks, P. & Hales, D. F. Numerous transposed sequences of mitochondrial cytochrome oxidase I-II in aphids of the genus *Sitobion* (Hemiptera: Aphididae). *MBE* **13**: 510-24 (1996).
91. Piggott, M. P. & Taylor, A. C. Extensive evaluation of faecal preservation and DNA extraction methods in Australian native and introduced species. *Aust. J. Zool.* **51**: 341-55 (2003).
92. Huntley, B., ScatMatch: Functions to aid with processing mass array data. 2022.
93. Mijangos, J. L., Gruber, B., Berry, O., Pacioni, C., & Georges, A. dartR v2: An accessible genetic analysis platform for conservation, ecology and agriculture. *MEE* **13**: 2150-58 (2022).
94. Hogg, C. J., et al. "Devil Tools & Tech": a synergy of conservation research and management practice. *Conserv. Lett.* **10**: 133-38 (2017).
95. Andrews, S. *FastQC A quality control tool for high throughput sequence data*. 2014 21 September 2020]; Available from: http://www.bioinformatics.babraham.ac.uk/projects/fastqc.
96. Kim, D., Paggi, J. M., Park, C., Bennett, C., & Salzberg, S. L. Graph-based genome alignment and genotyping with HISAT2 and HISAT-genotype. *Nat. Biotechnol.* **37**: 907-15 (2019).
97. Pertea, M., et al. StringTie enables improved reconstruction of a transcriptome from RNA-seq reads. *Nat. Biotechnol.* **33**: 290-95 (2015).
98. Kuo, R. I., et al. Illuminating the dark side of the human transcriptome with long read transcript sequencing. *BMC Genomics* **21**: 1-22 (2020).
99. Kang, Y.-J., et al. CPC2: a fast and accurate coding potential calculator based on sequence intrinsic features. *Nucleic Acids Res.* **45**: W12-W16 (2017).
100. Haas, B. J., et al. *De novo* transcript sequence reconstruction from RNA-seq using the Trinity platform for reference generation and analysis. *Nat. Protoc.* **8**: 1494-512 (2013).
101. Keilwagen, J., Hartung, F., & Grau, J. GeMoMa: Homology-based gene prediction utilizing intron position conservation and RNA-seq data. *Methods Mol. Biol.* **1962**: 161-77 (2019).
102. Smit, A., Hubley, R., & Green, P. *RepeatModeler Open-1.0*. 2008-2015 19 December 2019]; Available from: http://www.repeatmasker.org.
103. Smit, A., Hubley, R., & Green, P. *RepeatMasker Open-4.0*. 2013-2015 19 December 2019]; Available from: http://www.repeatmasker.org.
104. Seemann, T. barrnap 0.9: Rapid ribosomal RNA prediction. *barrnap 0.9: Rapid ribosomal RNA prediction* (2013).
105. Nawrocki, E. P. & Eddy, S. R. Infernal 1.1: 100-fold faster RNA homology searches. *Bioinform.* **29**: 2933-35 (2013).
106. Lovell, J. T., et al. GENESPACE tracks regions of interest and gene copy number variation across multiple genomes. *eLife* **11**: e78526 (2022).
107. Tian, R., et al. A chromosome-level genome of *Antechinus flavipes* provides a reference for an Australian marsupial genus with male death after mating. *Mol. Ecol. Resour.* **22**: 740-54 (2022).
108. Mikkelsen, T. S., et al. Genome of the marsupial *Monodelphis domestica* reveals innovation in non-coding sequences. *Nature* **447**: 167-77 (2007).
109. Stammnitz, M. R., et al. The evolution of two transmissible cancers in Tasmanian devils. *Science* **380**: 283-93 (2023).
110. Bond, D. M., et al. The admixed brushtail possum genome reveals invasion history in New Zealand and novel imprinted genes. *Nat. Comms.* **14**: 6364 (2023).
111. Tian, R., et al. A Chromosome-Level Genome of the Agile Gracile Mouse Opossum (*Gracilinanus agilis*). *Genome Biol. Evol.* **13** (2021).
112. Schwalb, B., et al., LSD: Lots of Superior Depictions. 2020.

113. Marín-Gual, L., et al. Strategies for meiotic sex chromosome dynamics and telomeric elongation in Marsupials. *PLos Genet.* **18** (2022).
114. Brandies, P. A., Tang, S., Johnson, R. S., Hogg, C. J., & Belov, K. The first antechinus reference genome provides a resource for investigating the genetic basis of semelparity and age-related neuropathologies. *GigaByte*: 1-22 (2020).
115. Johnson, R. N., et al. Adaptation and conservation insights from the koala genome. *Nat. Genet.* **50**: 1102-11 (2018).
116. Renfree, M. B., et al. Genome sequence of an Australian kangaroo, *Macropus eugenii*, provides insight into the evolution of mammalian reproduction and development. *Genome Biol.* **12**: 1-26 (2011).
117. Zhou, Y., et al. Platypus and echidna genomes reveal mammalian biology and evolution. *Nature* **592**: 756-62 (2021).
118. Yang, Z. PAML 4: Phylogenetic analysis by maximum likelihood. *MBE* **24**: 1586-91 (2007).
119. Jeffares, D. C., Tomiczek, B., Sojo, V., & dos Reis, M. A beginners guide to estimating the non-synonymous to synonymous rate ratio of all protein-coding genes in a genome. *Parasite Gen. Prot.*: 65-90 (2015).
120. Duchêne, D. A., et al. Analysis of phylogenomic tree space resolves relationships among marsupial families. *Syst. Biol.* **67**: 400-12 (2018).
121. Álvarez-Carretero, S., et al. A species-level timeline of mammal evolution integrating phylogenomic data. *Nature* **602**: 263-67 (2022).
122. Katoh, K. & Standley, D. M. MAFFT multiple sequence alignment software version 7: improvements in performance and usability. *MBE* **30**: 772-80 (2013).
123. Capella-Gutiérrez, S., Silla-Martínez, J. M., & Gabaldón, T. trimAl: a tool for automated alignment trimming in large-scale phylogenetic analyses. *Bioinform.* **25**: 1972-73 (2009).
124. Borowiec, M. L. AMAS: a fast tool for alignment manipulation and computing of summary statistics. *PeerJ* **4**: e1660 (2016).
125. Mendes, F. K., Vanderpool, D., Fulton, B., & Hahn, M. W. CAFE 5 models variation in evolutionary rates among gene families. *Bioinform.* **36**: 5516-18 (2020).
126. Niimura, Y. & Nei, M. Extensive gains and losses of olfactory receptor genes in mammalian evolution. *PLoS One* **2**: e708 (2007).
127. Niimura, Y., Matsui, A., & Touhara, K. Extreme expansion of the olfactory receptor gene repertoire in African elephants and evolutionary dynamics of orthologous gene groups in 13 placental mammals. *Genome Res.* **24**: 1485-96 (2014).
128. Niimura, Y., Matsui, A., & Touhara, K. Extreme expansion of the olfactory receptor gene repertoire in African elephants and evolutionary dynamics of orthologous gene groups in 13 placental mammals. *Genome Res.* **24**: 1485-96 (2014).
129. Altschul, S. F., Gish, W., Miller, W., Myers, E. W., & Lipman, D. J. Basic local alignment search tool. *J. Mol. Biol.* **215**: 403-10 (1990).
130. Eddy, S. R. Accelerated profile HMM searches. *PLoS Comput. Biol.* **7**: e1002195 (2011).
131. Morris, K. M., Cheng, Y., Warren, W., Papenfuss, A. T., & Belov, K. Identification and analysis of divergent immune gene families within the Tasmanian devil genome. *BMC Genomics* **16**: 1-12 (2015).
132. Peel, E., et al. Best genome sequencing strategies for annotation of complex immune gene families in wildlife. *GigaScience* **11** (2022).
133. Lefranc, M.-P., et al. IMGT®, the international ImMunoGeneTics information system® 25 years on. *Nucleic Acids Res.* **43**: D413-D22 (2015).
134. Johnson, R. N., et al. Adaptation and conservation insights from the koala genome. *Nat. Genet.* **50**: 1102-11 (2018).

135. Saitou, N. & Nei, M. The neighbor-joining method: a new method for reconstructing phylogenetic trees. *MBE* **4**: 406-25 (1987).
136. Felsenstein, J. Confidence limits on phylogenies: an approach using the bootstrap. *Evol.* **39**: 783-91 (1985).
137. Tamura, K., Stecher, G., & Kumar, S. MEGA11: Molecular evolutionary genetics analysis version 11. *MBE* **38**: 3022-27 (2021).
138. Belov, K., et al. Reconstructing an ancestral mammalian immune supercomplex from a marsupial major histocompatibility complex. *PLoS Bio.* **4**: e46 (2006).
139. Belov, K., Lam, M.-P., & Colgan, D. Marsupial MHC class II β genes are not orthologous to the eutherian β gene families. *J. Hered.* **95**: 338-45 (2004).
140. Braud, V. M., Allan, D. S., & McMichael, A. J. Functions of nonclassical MHC and non-MHC-encoded class I molecules. *Curr.Opin.Immunol.* **11**: 100-08 (1999).
