# Supplementary material for: Extant and extinct bilby genomes combined with Indigenous knowledge improve conservation of a unique Australian marsupial
Source: Nat Ecol Evol. 2024 Jul 1;8(7):1311–26. doi: 10.1038/s41559-024-02436-2 (PMC11239497; doi:10.1038/s41559-024-02436-2)
Supplement: Supplementary file 2 — Reporting Summary [file 41559_2024_2436_MOESM2_ESM.pdf]

nature portfolio

Corresponding author(s): Carolyn Hogg
Last updated by author(s): Mar 10, 2024

Reporting Summary

Nature Portfolio wishes to improve the reproducibility of the work that we publish. This form provides structure for consistency and transparency in reporting. For further information on Nature Portfolio policies, see our Editorial Policies and the Editorial Policy Checklist.

Statistics

For all statistical analyses, confirm that the following items are present in the figure legend, table legend, main text, or Methods section.

n/a	Confirmed	
☐	☒	The exact sample size (*n*) for each experimental group/condition, given as a discrete number and unit of measurement
☐	☒	A statement on whether measurements were taken from distinct samples or whether the same sample was measured repeatedly
☒	☐	The statistical test(s) used AND whether they are one- or two-sided<br>*Only common tests should be described solely by name; describe more complex techniques in the Methods section.*
☒	☐	A description of all covariates tested
☐	☒	A description of any assumptions or corrections, such as tests of normality and adjustment for multiple comparisons
☐	☒	A full description of the statistical parameters including central tendency (e.g. means) or other basic estimates (e.g. regression coefficient) AND variation (e.g. standard deviation) or associated estimates of uncertainty (e.g. confidence intervals)
☒	☐	For null hypothesis testing, the test statistic (e.g. *F*, *t*, *r*) with confidence intervals, effect sizes, degrees of freedom and *P* value noted<br>*Give P values as exact values whenever suitable.*
☒	☐	For Bayesian analysis, information on the choice of priors and Markov chain Monte Carlo settings
☒	☐	For hierarchical and complex designs, identification of the appropriate level for tests and full reporting of outcomes
☒	☐	Estimates of effect sizes (e.g. Cohen's *d*, Pearson's *r*), indicating how they were calculated

Our web collection on statistics for biologists contains articles on many of the points above.

Software and code

Policy information about availability of computer code

Data collection	PacBIO SMRT Link v9.0.0.92188; DRAGEN Germline platform v3.8.4; MassARRAY TyperAnalyzer v4.1
Data analysis	PacBio's Improved Phased Assembler (IPA) v1.1.2; Purge_dups v1.2.3; Long Ranger v2.2.2; Burrows–Wheeler Aligner (BWA) mem v0.7.17-r1188; samtools v1.9; ARCS v1.1.1; LINKS v1.8.7; PBJelly v15.8.24; Pilon v1.20; BBDuk v37.98; HiRise v2.1.6; pairtools v0.3.0; cooler v0.8.10; hicExplorer v3.6; HiGlass v2.1.11; BBTools v38.73; DRAGEN Joint Genotyping v3.8.4. Bcftools v1.11; ANNOVAR v20180416; PLINK v1.90; Picard v2.21.925; GATK v4.2.0.0; VCFtools v0.1.1427; BEDtools v2.29.2; COANCESTRY v1.0; StAMPP package v1.6.3; NeEstimator v2.1; dartR v1.9.6; SNPRelate v0.9.19; HISAT2 v2.1.0; StringTie v2.1.3; TAMA merge v0.0; TransDecoder v2.0.1; StringTie v2.1.3; GeMoMa v1.8; GENESPACE v1.3.1; OrthoFinder v2.4.01; PAML v4.9; CAFE v.5.0; BLASTp v2.2.30; HMMER v3.2; https://github.com/PacificBiosciences/pbipa; https://github.com/wtsi-hpag/PretextMap; https://github.com/wtsi-hpag/PretextView; https://github.com/wtsi-hpag/PretextSnapshot; Stacks v2.61; Trimommatic v0.39; hierfstat package v0.5-10; diveRsity package v1.9.90

For manuscripts utilizing custom algorithms or software that are central to the research but not yet described in published literature, software must be made available to editors and reviewers. We strongly encourage code deposition in a community repository (e.g. GitHub). See the Nature Portfolio guidelines for submitting code & software for further information.

Data

Policy information about availability of data

All manuscripts must include a data availability statement. This statement should provide the following information, where applicable:

- Accession codes, unique identifiers, or web links for publicly available datasets
- A description of any restrictions on data availability
- For clinical datasets or third party data, please ensure that the statement adheres to our policy

Data Availability
Raw and processed data for the reference genome, transcriptomes and resequenced genomes is available via NCBI for the Ninu (PRJNA1049866) and Yallara (PRJNA1049868); in addition to the Australasian Genomes website (https://awgg-lab.github.io/australasiangenomes/genomes.html). The DArTseq SNP genotypes for population genetic analysis and the MassARRAY scat genotyping assay are available at Dryad (https://doi.org/10.5061/dryad.gtht76htz).

Not included in paper but note for Editor & reviewers, the Dryad link will not go live until paper is accepted, in the meantime the following temporary link can be used to provide reviewers/editors access: https://datadryad.org/stash/share/Y8xsPa_64Po7w4bHE_0xfZJoC9VLBIDk0JlKppSL6ZA.

Code availability
The code used to select SNPs to design the custom MassARRAY scat genotyping assay is provided as Supplementary Text File. All other analyses used standard software and scripts as described in the Methods and Supplementary.

Research involving human participants, their data, or biological material

Policy information about studies with human participants or human data. See also policy information about sex, gender (identity/presentation), and sexual orientation and race, ethnicity and racism.

Reporting on sex and gender	Not Applicable
Reporting on race, ethnicity, or other socially relevant groupings	Not Applicable
Population characteristics	Not Applicable
Recruitment	Not Applicable
Ethics oversight	Not Applicable

Note that full information on the approval of the study protocol must also be provided in the manuscript.

Field-specific reporting

Please select the one below that is the best fit for your research. If you are not sure, read the appropriate sections before making your selection.

☐ Life sciences ☐ Behavioural & social sciences ☒ Ecological, evolutionary & environmental sciences

For a reference copy of the document with all sections, see nature.com/documents/nr-reporting-summary-flat.pdf

Ecological, evolutionary & environmental sciences study design

All studies must disclose on these points even when the disclosure is negative.

Study description	This study characterises the genomes of the extant greater bilby and the extinct lesser bilby. We use this genome to undertake a genetic assessment of bilbies in the metapopulation, and develop and test a new scat SNP array for use by Indigenous Rangers.
Research sample	The Greater bilby reference genome was a female individual euthanised for medical reasons - we collected spleen, liver, lymph node, kidney, heart, tongue, ovary, uterus, pouch skin, mammary gland and salivary gland. We also collected testes from a medically euthanised male. The Lesser bilby samples were skin or bone from museums. The metapopulation samples were ear biopsies from 363 individuals; 46 scat samples were collected from a fenced location and wild sites.
Sampling strategy	No sample-size calculation was performed as bilbies are extremely difficult to catch. We asked each location to provide samples for all individuals if less than 20 were housed there, and 20 samples for locations with greater than 20 individuals (this is based on previous work that 20-30 individuals captures at least 95% of diversity within a location).
Data collection	Metadata for all samples were provided by the institution holding the individuals, this included date collected, individual ID and location. The scat samples were collected by the Kiwirrkurra Indigenous rangers.
Timing and spatial scale	The female reference individual was euthanised in 2018; the male was euthanised in 2021. The metapopulation samples were

Timing and spatial scale	collected between 2011 and 2022, dates and samples sizes for each are provided in the supplementary. These samples were collected during surveys and translocation events. The scat samples were collected in 2021 and 2022.
Data exclusions	Samples were only excluded if poor sequencing coverage was obtained.
Reproducibility	Technical replicates were used for both the metapopulation study and the SNP array so we can understand the reproducibility of the sequencing methods and compare results. This data is presented in the supplementary.
Randomization	As this was a genetic diversity survey of the whole metapopulation, and development of a new SNP methodology, no randomization was applied.
Blinding	As this was a genetic diversity survey of the whole metapopulation, and development of a new SNP methodology, no blinding was applied.

Did the study involve field work? ☒ Yes ☐ No

Field work, collection and transport

Field conditions	Samples were collected across Australia, due to the size of the continent it is hard to describe field conditions at each site. Collectively bilbies are located primarily in semi-arid areas, so very dry and hot. Samples were collected during the winter trapping months, and early in the morning. Scat samples were collected in the winter months, early in the morning.
Location	Arid Recovery -30.3777293 136.9255; Kimberley (wild) -17.3492 125.9152; Pilbara (wild) -21.7016 120.2511; Scotia -33.39638 141.31305; Thistle Island -35.0381 136.1805; Venus Bay -38.6763 145.7925; Yookamurra -34.5049 139.475358; ZAA -34.91448 138.60651; ZAA -23.70641 133.832568; ZAA -34.9672892 138.696397; ZAA -28.135129 153.488807; ZAA -27.863336 153.315564; ZAA -32.0203936 116.040308; ZAA -35.0888 139.16183; ZAA -33.839442 151.239365; Currawinya -28.84397 144.49557; Dubbo -32.2818 148.57115; Mallee Cliffs -34.21134 142.624833; Mt Gibson -29.63002 117.23731; Pilliga -30.4966464 148.7266; Kiwirrkurra -22.8161 127.7644
Access & import/export	All samples were collected under permit held by the institutions providing the samples, under their standard operating procedures and shared with us for the purposes of managing their populations. All samples were held at the University of Sydney under NSW Scientific Permit SL101204.
Disturbance	Bilbies were trapped according to organisational standard operating procedures for the capture, handling and movement of this species. Efforts are made to minmise disturbance whilst trapping and individuals are either trapped and handled at night, or very early in the morning to minimise stress.

Reporting for specific materials, systems and methods

We require information from authors about some types of materials, experimental systems and methods used in many studies. Here, indicate whether each material, system or method listed is relevant to your study. If you are not sure if a list item applies to your research, read the appropriate section before selecting a response.

Materials & experimental systems

n/a	Involved in the study	
☒	☐	Antibodies
☒	☐	Eukaryotic cell lines
☒	☐	Palaeontology and archaeology
☐	☒	Animals and other organisms
☒	☐	Clinical data
☒	☐	Dual use research of concern
☒	☐	Plants

Methods

n/a	Involved in the study	
☒	☐	ChIP-seq
☒	☐	Flow cytometry
☒	☐	MRI-based neuroimaging

Animals and other research organisms

Policy information about studies involving animals; ARRIVE guidelines recommended for reporting animal research, and Sex and Gender in Research

Laboratory animals	Not applicable
Wild animals	The metapopulation samples came from individuals housed in fenced sanctuaries, zoos, or islands. Bilbies were trapped as per standard operating procedures using a cage trap covered with a hessian sack and baited with oats and peanut butter, or captured with a hoop net. Sampling was undertaken at night or very early in the morning. Bilbies were released back to their trapping location, unless they were being translocated as part of the metapopulation management. Wild bilby samples were collected from roadkill, or

	individuals that were trapped and released. The reference genome samples came from a female and male that were medically euthanised at their zoo location.
Reporting on sex	All individuals were sexed by the trapping teams by observing their genitalia.
Field-collected samples	No individuals were housed in laboratories.
Ethics oversight	Tissue samples for the reference individual, and the male testis, were collected opportunistically when individuals were euthanised for medical purposes. Ear biopsies are collected as part of the metapopulation routine monitoring programs, or during targeted trapping and capture events, that they conducted in accordance with the standard operating procedures for each organization. These management samples were shared with us as part of a study plan approved by representatives from the participating ZAA facilities, the AWC, the Australian Museum, the University of Sydney, and the Greater Bilby National Recovery Team Metapopulation Committee.

Note that full information on the approval of the study protocol must also be provided in the manuscript.

Plants

Seed stocks	*Report on the source of all seed stocks or other plant material used. If applicable, state the seed stock centre and catalogue number. If plant specimens were collected from the field, describe the collection location, date and sampling procedures.*
Novel plant genotypes	*Describe the methods by which all novel plant genotypes were produced. This includes those generated by transgenic approaches, gene editing, chemical/radiation-based mutagenesis and hybridization. For transgenic lines, describe the transformation method, the number of independent lines analyzed and the generation upon which experiments were performed. For gene-edited lines, describe the editor used, the endogenous sequence targeted for editing, the targeting guide RNA sequence (if applicable) and how the editor was applied.*
Authentication	*Describe any authentication procedures for each seed stock used or novel genotype generated. Describe any experiments used to assess the effect of a mutation and, where applicable, how potential secondary effects (e.g. second site T-DNA insertions, mosiacism, off-target gene editing) were examined.*
